# Novel fast Li-ion conductors for solid-state electrolytes from first-principles

**DOI:** 10.1039/d5ee07336g

**Published:** 2026-04-22

**Authors:** Tushar Singh Thakur, Loris Ercole, Nicola Marzari

**Affiliations:** a Theory and Simulation of Materials (THEOS), and National Centre for Computational Design and Discovery of Novel Materials (MARVEL), École Polytechnique Fédérale de Lausanne 1015 Lausanne Switzerland tushar.thakur@epfl.ch; b PSI Center for Scientific Computing, Theory and Data, Paul Scherrer Institute 5232 Villigen PSI Switzerland; c Theory of Condensed Matter, Cavendish Laboratory, University of Cambridge Cambridge CB3 0US UK

## Abstract

We present a high-throughput computational screening for fast lithium-ion conductors to identify promising materials for application in all solid-state electrolytes. Starting from more than 30 000 Li-containing experimental structures sourced from the Crystallography Open Database, Inorganic Crystal Structure Database and Materials Platform for Data Science, we perform highly automated calculations to identify electronic insulators. On these ∼1000 structures, we use molecular dynamics simulations to estimate Li-ion diffusivities using the pinball model, which describes the potential energy landscape of diffusing lithium with accuracy similar to density functional theory while being 200–500 times faster. Then we study the ∼60 most promising and previously unknown fast conductors using full first-principles molecular dynamics simulations at several temperatures to estimate their activation barriers. The results are discussed in detail for the 9 fastest conductors, including Li_7_NbO_6_, which shows a remarkable ionic conductivity of ∼5 mS cm^−1^ at room temperature. We further present the entire screening protocol, including the workflows where the accuracy of the pinball model is improved self-consistently, necessary for automatically running the required calculations and analysing their results.

## Abstract 2

We present a high-throughput computational screening for fast lithium-ion conductors to identify promising materials for application in all solid-state electrolytes.

## Graphical abstract

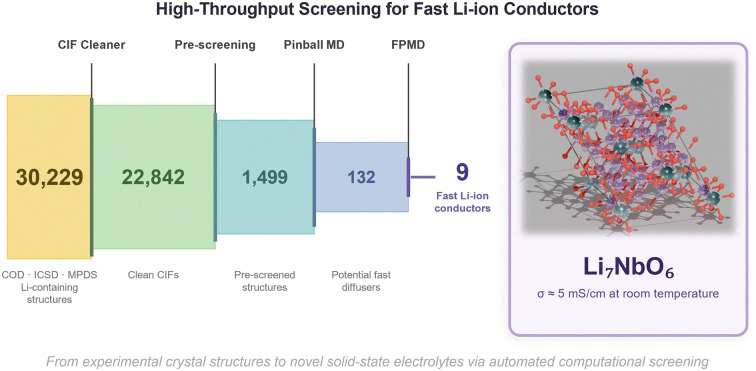

**Broader context**

Solid-state electrolytes have emerged as key components in the development of the next generation of energy storage devices. Their inherent safety and superior performance compared to conventional liquid electrolytes have attracted increased attention in the field of sustainable energy. Despite the tremendous attention, the design and discovery of a novel solid-state electrolyte with high Li-ion conductivity remain a significant challenge. While many structural families have been identified over the years, the progress has been slow and discovering new fast Li-ion conductors for solid-state electrolytes would have a major impact. Unlike all experimental procedures that can be human intensive, computational methods for automated discovery are readily parallelisable and require much fewer resources. Nevertheless, a computational strategy relying on full first-principles methods can be exceptionally expensive, and hence there is a need for methods that are sufficiently inexpensive to be able to run thousands of appropriate calculations while being accurate enough to yield meaningfully predictive results. This screening identifies fast Li-ion conductors by estimating Li-ion diffusivity using molecular dynamics simulations with the pinball model. This approach is typically two orders of magnitude faster than first-principles molecular dynamics simulations while retaining a similar level of accuracy. We emphasise that we exclusively study experimentally known materials, ensuring that the fast ionic conductors we suggest are actually synthesisable and ready for in-depth experimental investigation.

## 1 Introduction

All-solid-state Li-ion batteries (ASSLBs) have been intensively studied^1–3^ particularly for applications in electric vehicles^4,5^ and mobile devices.^6^ This growing interest is primarily attributed to ASSLBs' higher energy densities and enhanced safety profiles compared to their conventional liquid counterparts.^2,5,7^ Besides this, ASSLBs' lightweight nature facilitates improved battery miniaturisation and easier assembly process,^8^ and they exhibit superior mechanical, thermal and electrochemical stability.^9,10^ Despite the significant attention ASSLBs have received, no known solid-state material satisfies all of the desirable requirements needed for their application, including high ionic conductivity.^9,11^ While many structural families have been identified, progress remains slow, underscoring the importance of searching new materials for ASSLBs.^12,13^

In the past, materials discovery has relied on experimental approaches guided by chemical intuition.^14^ As a first example, phosphate based Li-containing materials were derived from NASICONs (Na Super Ionic CONductors)^15,16^ with the structural formula LiM_2_(PO_4_)_3_ (M = Ti, Zr).^17,18^ These so-called Li-NASICONs exhibit high Li-ion conductivity^19^ and continue to be the subject of ongoing research.^20,21^ Further examples include the gradual and systematic exploration of various inorganic families such as nitrides,^22,23^ halides,^24,25^ hydrides,^26,27^ perovskites with the general formula La_3*x*_La_2/3−*x*_TiO_3_^28,29^ and Li-argyrodites with the formula Li_6_PS_5_X (X = Cl, Br, I).^30^ A final example is the development of Li-containing garnet structures, with chemical composition Li_5_La_3_M_2_O_12_ (M = Ta, Nb), which were identified to be promising conductors, albeit with limited ionic conductivity.^31^ However, the chemical substitution with aliovalent ions led to the discovery of Li_7_La_3_Zr_2_O_12_, commonly known as LLZO, that demonstrates significantly higher ionic conductivity.^32^

The development of LLZO also serves as an example of chemical substitution in well-known ionic conductor families to explore the vast chemical space and identify new ionic conductors. Another example is the extensively studied family of LISICONs (Li-superionic conductors) with the formula Li_14_Zn(GeO_4_)_4_.^33^ Over time, numerous new LISICON-type materials were discovered,^34–37^ which can be represented by a more general formula of Li_4_XO_4_−Li_3_YO_4_ (X = Si, Ge, Ti; Y = P, As, V).^14^ LISICONs also serve as the precursors to the thio-LISICON family,^38^ which consists of a more polarisable sulphide anionic framework rather than an oxide sublattice, thereby enhancing their ionic conductivity.^39^ Further substitution of the cations led to the discovery of tetragonal-Li_10_GeP_2_S_12_ (LGPS),^40^ which is widely regarded as one of the best solid-state ionic conductors^11^ and has motivated the development of numerous promising derivative structures.^41^ To summarise, significant breakthroughs have primarily resulted from chemical intuition or by systematic substitution in known materials, motivated by the keen understanding of the underlying chemistry. Besides this, combinatorial methods^42^ and straightforward high-throughput experimental approaches^43,44^ have also contributed to the discovery of new superionic conductors, albeit with mixed success.

**Fig. 1:**
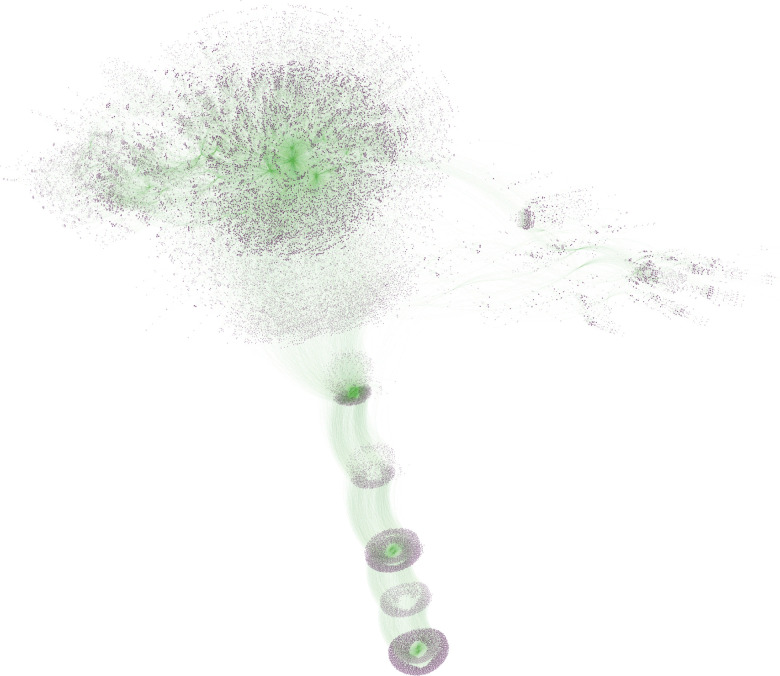
A segment of the AiiDA database spanning this screening is depicted, showcasing a small subset pertaining to single-point calculations performed on approximately 1500 structures at the level of DFT. Purple nodes represent either data instances (*i.e.*, inputs and outputs of calculations) or the calculations themselves, while green links illustrate the logical provenance connecting these nodes.

However, these experimental approaches do not scale as effectively as computational methods, which can be highly efficient in materials discovery by allowing for the exploration of a vast number of structural families within a short time frame (Fig. 1).^45–47^ Furthermore, computer simulations have primarily been limited to understanding the underlying diffusion mechanism, which in turns contributes to developing deeper chemical intuition. As a result, many computational screenings are typically motivated by established chemical knowledge, focusing on specific ion-conduction mechanisms or space-groups to propose new materials.^48^ For instance, Xiao *et al.*^49^ performed a computational screening motivated by the diffusion network in garnets and NASICON type conductors; Muy *et al.*^50^ explored all the possible doping strategies within the argyrodite family. In contrast, a screening approach that is agnostic to the underlying chemistry of structures can probe a much more expansive chemical space and potentially identify novel materials that have no apparent connection to the existing materials.

Consequently, it is essential to establish robust screening criteria motivated by physical properties to effectively identify the most suitable candidates for solid-state electrolytes. To prevent self-discharge in a battery, an SSE ought to exhibit low electron mobility, which is determined by the material's electronic band gap. The most accessible first-principles method for estimating band gaps is Kohn–Sham density functional theory (DFT).^51,52^ Although more advanced approaches, such as GW,^53^ Koopmans-compliant functionals,^54^ hybrid functionals,^55^ Hubbard-corrected DFT^56^ and many others,^57^ can yield band gap values that are predictive, these methods are significantly more computationally demanding compared to single point DFT calculation. Thus, for screening purposes, DFT offers a satisfactory balance between computational efficiency and accuracy for band gap estimates, despite its tendency to underestimate band gaps.^58^ This was also utilised by the screening studies of Muy *et al.*^59^ and Sendek *et al.*,^60^ who calculated band gaps at the level of DFT-PBE^61^ and applied a filtering criterion treating any material with a band gap greater than 1 eV as an insulator.

Electrochemical stability can be estimated using first-principles calculations as well,^62^ and it can be estimated in a high-throughput mode.^63^ While a broad electrochemical stability window is desirable for SSEs, many currently utilised electrolytes exhibit narrow stability windows.^14^ A notable example is LGPS which is stabilised with interphases and protective coatings.^64,65^ In the same vein, although low interfacial resistance and high interfacial compatibility between electrolytes and electrodes are important for optimum performance, higher resistance (and lower compatibility) can be mitigated by incorporating appropriate interfacial materials.^65,66^ Therefore, we emphasise that while electrochemical stability and interfacial compatibility are important considerations, they are not essential for a screening process, and thus, we have opted not to calculate these in this study.

Mechanical properties such as bulk and shear moduli can be readily obtained from simulations.^67,68^ However, the relevance of this information remains somewhat ambiguous. For instance, preventing or retarding the unwanted growth of Li-dendrites is achieved not merely through the use of a high-modulus material, but rather through defect engineering.^69,70^ Consequently, while bulk properties can be calculated easily, their utility as screening criteria is not well understood and as such we have chosen not to incorporate them in our study.

In summary, many challenges persist that limit the selection of materials for use as SSEs;^71,72^ still, achieving high ionic conductivity remains the most critical criterion.^73,74^ Ionic diffusion can be estimated from atomistic simulations directly through MD,^75,76^ with the accuracy dependent on the underlying potential energy surface (PES), which can be computed using empirical or machine-learned force-fields or using first-principles methods.^77–79^ While empirical force-fields may be sufficiently accurate to model Li-diffusion,^37,80^ they require precise fitting of the parameters to the specific system under consideration, which limits their applicability in exploring a vast chemical space. DFT can provide highly accurate and general PES applicable to a wide variety of chemical compositions. However, first-principles molecular dynamics (FPMD) in the Born–Oppenheimer^81^ approximation relies on performing single point DFT calculations at every MD step, rendering it prohibitively expensive.^82^ Another variant of FPMD, Car–Parrinello molecular dynamics,^83^ is computationally more efficient, but requires careful tuning to the system being studied. While this method can be highly useful for investigating diffusion mechanisms within a single system,^84^ it is non-trivial to calibrate its parameters across a multitude of systems. In addition to MD, ionic conductivity can be estimated in the simplest Arrhenius picture by calculating migration barriers for Li-diffusion, which can be obtained from inexpensive static calculations.^85,86^ However, identifying barriers is a highly complex task that often requires human intervention and is thus challenging to automate.^87–89^ Other methods attempt to link diffusion to more easily accessible properties: for example, the bond-valence method^90^ has been used to inexpensively calculate Li-ion conductivity in several independent screenings,^91–93^ though with limited accuracy due to the limitations of the method.^94,95^ Another approach involved deriving diffusion coefficients using specific phonon frequencies.^96,97^ In all cases, the aim to reduce computational costs goes directly against the requirement of reliable predictions across a broad range of materials.

In the past few years, universal machine learning interatomic potentials (MLIPs) have also emerged as an one-stop solution for running cheap and accurate MD simulations, including MACE-MP0,^98,99^ M3Gnet,^100^ CHGnet^101^ and the proprietary GNOME.^102^ These universal MLIPs are intended to be systems agnostic, can supposedly model most elements in the periodic table, and most importantly work out-of-the-box. Before deployment, their suitability needs to be thoroughly tested. Besides the initial applications, a few independent performance assessments of the universality have been performed.^103–106^ Both Yu *et al.*^104^ and Focassio *et al.*^105^ concluded that universal MLIPs are not yet accurate enough to reproduce first-principles results and show significant error in the estimation of properties under consideration. Both suggested that the current best use case is as a foundation onto which a more appropriate model can be trained. These shortcomings are also noted by the original authors.^99^ Nevertheless, these universal MLIPs promise a most promising way forward and are starting to be employed in high-throughput screenings.^107,108^

Besides universal MLIPs, several other powerful predictive models exist.^109^ The most common approach is to use descriptors to directly predict properties, like ionic conductivity, from the structures and or chemical phase space,^59,110,111^ by unsupervised or semi-supervised learning due to the lack of labelled data,^112^ or atypically by training directly on experimental data.^113^ Another approach that has garnered significant attention in the past year is inverse modelling, facilitated by artificial intelligence for materials discovery.^102,114^ These methods involve proposing hypothetical materials that may not necessarily be experimentally synthesisable.^100,101^ Nonetheless, predicting materials that are not merely synthesisable but also technologically relevant is highly non-trivial,^115^ which suggests that the underlying premise may require further examination.^116^ This stands in direct contrast to the present work, where we screen experimentally known materials whose synthesis recipes are known. It is important to note that several well-regarded screenings in the past few years^59,110,117,118^ also utilised structures from the same repositories as ours. However, our workflow was able to identify promising conductors that were not highlighted in those earlier efforts, underscoring the effectiveness of our approach.

We conclude this brief review of computational methods for modelling ionic diffusion by noting that screening fast Li-ion conductors remains a challenging undertaking. This difficulty arises either from the limited transferability and/or accuracy of descriptors, force-fields and universal MLIPs or due to the cost of first-principles approaches. Thus, accurately modelling the diffusion of Li-ions in a large-scale screening with MD simulations necessitates a computational approach that combines the low computational cost of force-fields with the precision and generality of DFT. In this study, this is achieved using the pinball model, which describes the potential energy surface of lithium diffusing in an SSE and is on average about 200–500 times faster than DFT, while offering often comparable accuracy.^119^ It is based on two key assumptions: (1) all Li atoms are completely ionised and are referred to as pinballs and (2) the host lattice (all non-Li atoms along with the valence electrons of Li atoms) is fixed at the equilibrium positions, and the charge density is frozen. The pinball model forms the backbone of our screening, as detailed in Section 2.3.1, enabling the identification of promising Li-ion conductors for further investigation using full first-principles simulations.

As a final note, we highlight a previous screening^117^ conducted using a similar framework based on the pinball model. The critical distinctions are as follows: (1) the present study utilises a more expansive database, encompassing over twice the number of structures; (2) we include non-local interactions within the pinball model; (3) we have implemented a self-consistent workflow that iteratively enhances the accuracy of the pinball model; (4) we apply more stringent criteria across all filtering parameters, for instance by tightening the tolerances used to compare crystal structures, we classify nearly 30% more structures as duplicates in this screening; (5) we use different functionals and pseudopotentials along with different sets of input parameters for all electronic structure calculations. These differences and the advantages they offer are described in more detail along with methods in Section 2, followed by a discussion of results in Section 3. Last, we summarise this screening and present our conclusions, followed by an outlook on the development of a universal machine learning potential to model Li-ion diffusion in Section 4.

## 2 Methods

**Fig. 2:**
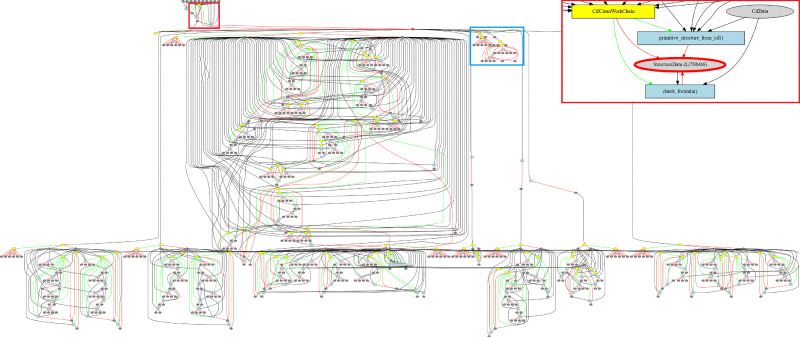
The provenance graph for one material, Li_7_NbO_6_, illustrates AiiDA's meticulously tracking of each instance of input and output, along with all intermediate data and steps, as a directed acyclic graph. Nodes in the graph are colour-coded to denote different elements: workflows are highlighted in yellow, calculations in blue, and data instances in grey. Data instances, which can represent either inputs or outputs of calculations, are connected by black lines. Red lines signify logical provenance, *i.e.* a workflow outputting a data instance, while green lines denote operational provenance, illustrating the invocation of one workflow or calculation by another. The highlighted sub-graph provides a detailed view of the structure ingestion shown in a red box, the band gap calculation and variable-cell relaxation are given within the blue box, and the remaining graph corresponds to the self-consistent pinball MD simulations.

Any computational screening of this magnitude requires a robust framework to automatically launch and monitor calculations, handle errors on-the-fly, and link data generated during calculations.^120,121^ Furthermore, it is necessary that this infrastructure explicitly preserves the provenance for easy reproducibility, queryability, and shareability of the results.^122,123^ To achieve this twofold goal of automating and managing complex workflows and storing full provenance of all related data, we used the Automated Interactive Infrastructure and Database for Computational Science (AiiDA), which is a Python-based infrastructure and workflow manager.^46,124,125^ The key advantage of AiiDA over other workflow managers lies in its ability to preserve the provenance of a calculation in its entirety. This includes storing the complete history of a calculation along with an exhaustive list of all inputs that led to the creation of that piece of data, as a directed acyclic graph within a relational database. This feature allows one to query any data point as a graph node in an easy to navigate fashion and assess the causal relationship between nodes. Fig. 2 illustrates this capability in an acyclic graph, taken from this work, that illustrates the entire screening path for one structure. This approach not only supports Open Science but goes beyond the well-known FAIR principle.^126^ Additionally, AiiDA facilitates a high degree of automation and parallelisation to easily run calculations on high-performance computing platforms, and every calculation in this screening is run using AiiDA.

### 2.1 Preliminary filters

**Fig. 3:**
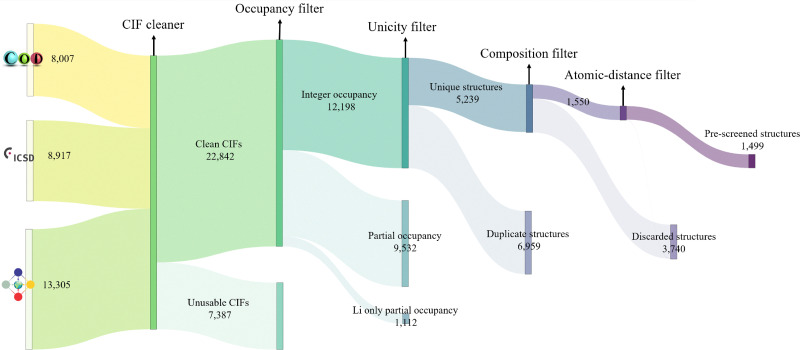
Flowchart illustrating the pre-screening workflow, beginning with all Li-containing structures sourced from COD, ICSD and MPDS, and culminating with *ab initio* calculations. Each node represents a filter that eliminates undesirable structures (indicated by lighter shaded links), while potentially suitable structures advance to the next filter (indicated by darker shaded links). The link thickness corresponds to the number of structures passing through each filter. Beginning with over 30 000 experimental structures, the pre-screening narrows the selection down to 1499 structures for subsequent electronic structure calculations.

Starting from experimental structures sourced from the Crystallography Open Database (COD),^127^ Inorganic Crystal Structure Database (ICSD)^128^ and Materials Platform for Data Science (MPDS)^129^ repositories, we identify more than 30 000 lithium containing structures, which are imported as CIF files using AiiDA. These files sometimes contain syntax errors or extraneous information that require correction before they can be used. The issues and their corresponding solutions are comprehensively described in the work by Mounet *et al.*^47^ We follow that protocol to clean, parse and standardise CIF files using COD-tools.^130–132^ Finally, on the cleaned CIF files, we apply a sequence of filters to systematically narrow down the list of promising structures, as illustrated in Fig. 3.

#### 2.1.1 Occupancy filter

We remove structures with partial occupancies *i.e.* those whose stoichiometry doesn't align with the reported atomic positions, as generating and modelling derivative configurations necessitate sampling strategies that can be highly non-trivial.^133–135^

#### 2.1.2 Unicity filter

Subsequently, we use the CMPZ algorithm^136^ implemented within the structure matcher function of pymatgen^137^ to compare crystal structures with the same stoichiometry, to eliminate equivalent structures and retain only unique ones.

#### 2.1.3 Composition filter

Additionally, we exclude structures containing certain elements. Specifically, we filter out those containing hydrogen, as elements lighter than lithium cannot be correctly modelled by the pinball approximation; 3d-transition elements, due to their potential to change oxidation states during simulations and become electronically conducting; noble gas atoms and elements heavier than polonium. Furthermore, we apply additional filtering criteria to ensure that each structure contains a specific selection of anions from the pnictogen, chalcogen and halogen families.

#### 2.1.4 Atomic distance filter

For each structure, we calculate the bond distances between every atom pair that is compatible with inorganic materials to filter out structures with bond lengths typically associated with organic molecules such as double bonds with O or triple bonds with N.

We note that thus far we have conducted data analysis. The subsequent sections describe the final two filters wherein we perform electronic-structure calculations.

### 2.2 Electronic filters

To classify the filtered structures as electronic insulators, we calculate the band gap at the level of DFT. As a rule of thumb, we categorise structures with a band gap greater than 1 eV as electronically insulating. Generally, DFT underestimates the band gap for most materials.^58^ All DFT calculations are performed using the pw.x code from the Quantum ESPRESSO distribution,^138,139^ using experimental geometry, and with the PBEsol^61,140^ exchange–correlation functional. Pseudopotentials and their corresponding cut-offs are sourced from the Standard Solid-State Pseudopotential (SSSP) Efficiency 1.2.1 library,^141^ which provides comprehensive validation of pseudopotentials across various libraries and methods.^142–147^ For each SCF calculation, we use Marzari-Vanderbilt cold smearing^148^ and increase the number of bands by 20%, while the Brillouin zone is sampled with a Monkhorst–Pack grid of density 0.15 Å^−1^.

Besides this, we perform variable-cell relaxation on about 20% of the structures to investigate the effects of geometry optimisation on band gap estimation.

### 2.3 Diffusivity filter

To run MD simulations, we generate supercells based on experimental geometries, ensuring a minimum separation of 8 Å between opposite faces, using the supercellor package.^150^ We run MD simulations with Born–Oppenheimer approximation^81^ in the canonical ensemble. Temperature is controlled with the stochastic velocity rescaling thermostat.^151^

From the Einstein relation^78^ we can write the tracer diffusion coefficient *D*_tr_ as:1
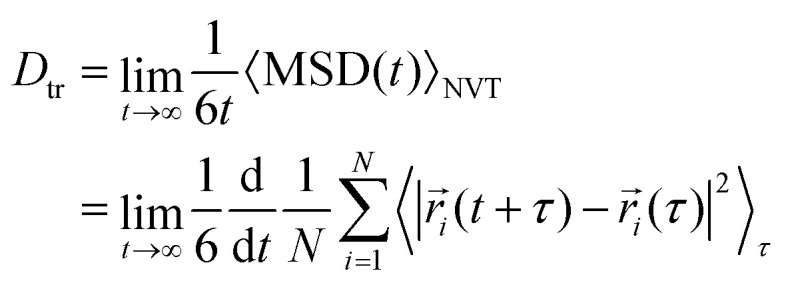
which is a derivative of the average mean-square displacement of particles with respect to time. In this context, we are essentially substituting the ensemble average with a time average. By performing a linear regression of the mean square displacement MSD(*t*) with time we can accurately estimate the diffusion coefficient from the slope of the MSD, ensuring sufficient statistical precision.

The tracer diffusion coefficient is related to the charge diffusion coefficient with Haven's ratio as *H* = *D*_tr_/*D*_*σ*_, which is a measure of correlated motion of the particles.^152^ In the dilute limit, we assume it to be 1, though in practice it is often less than 1, implying that correlated motion can enhance conductivity.^87^ Consequently, we do not overestimate conductivities. And from the Nernst–Einstein equation,^153^ we can calculate the ionic conductivity *σ* as:2
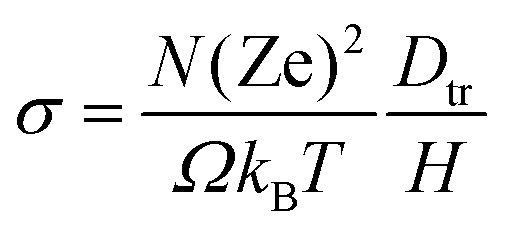
where *Ω* is the system volume, *T* is the temperature, and Ze is an integer multiple of the elementary charge.

All analysis of trajectories including the calculation of MSD was done using the open-source tool Suite for analysis of molecular simulations (SAMOS).^154^

#### 2.3.1 Self-consistent pinball MD

Based on the two assumptions of the pinball model,^119^ the Hamiltonian reads as:3
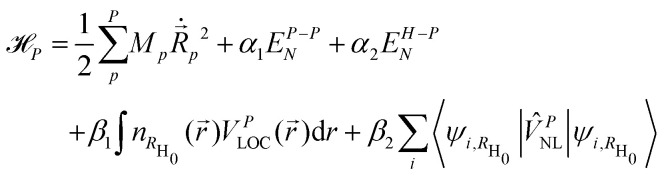
where *R⃑* and 
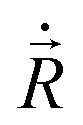
 are respectively the positions and velocities of the pinballs *i.e.* the Li-ions, *E*^A−B^_N_ is the electrostatic interaction between the frozen core electrons of species A and B, *V*^P^_LOC/NL_ are the local and non-local external pseudopotential components of pinballs, which act on the charge density 
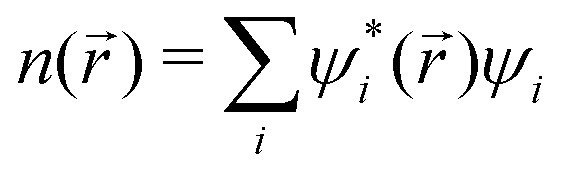
, which is frozen for the host lattice *H*_0_. The final term is responsible for non-local interactions, which further improves the accuracy of the model with additional computational cost. *α*_1_, *α*_2_, *β*_1_ and *β*_2_ are phenomenological coefficients (referred to as pinball coefficients) introduced to further improve the accuracy that can be computed by fitting the pinball forces with DFT forces.

**Fig. 4:**
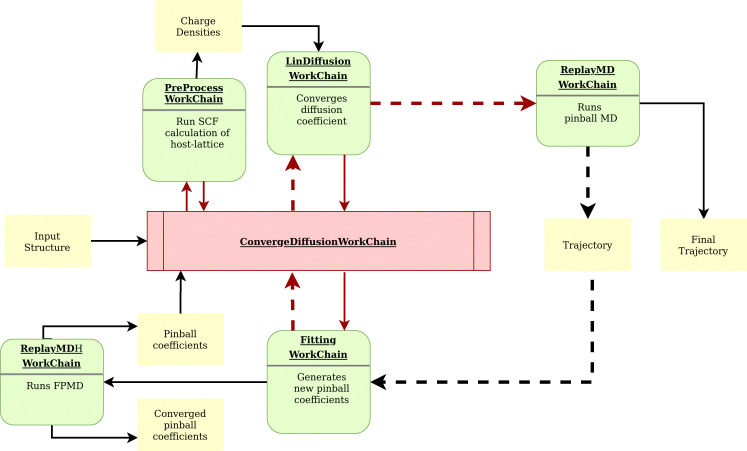
A schematic representation of the self-consistent workflow of AiiDA-flipper,^149^ the python package employed to run MD simulations using the pinball model^119^ and compute ionic conductivity of lithium. The nomenclature depicted corresponds exactly to the Python classes within the plugin. The ConvergeDiffusion workchain initiates the process by launching the PreProcess workchain, which runs a single point calculation and stores the charge densities of the host lattice to be used in all subsequent pinball MD simulations. Next, the Fitting workchain is launched, generating sufficient snapshots with random displacement of Li-ions in the supercell to fit 10 000 force components through calculations at both the pinball and DFT levels. This initial estimate of pinball coefficients is then used to initiate the LinDiffusion workchain, which runs a long MD simulation at the pinball level to converge the diffusion coefficient to a predetermined threshold. From the trajectory of this MD run, uncorrelated configurations are extracted, and a new set of pinball coefficients are derived through linear regression of the DFT and pinball forces. This iterative cycle continues self-consistently until the pinball coefficients converge. Once convergence is achieved, a final MD simulation is performed using the converged pinball coefficients, and the final MD trajectory is used to compute the diffusion coefficient. This workflow ensures accurate and reliable computation of ionic conductivity, leveraging the self-consistent refinement of pinball coefficients through iterative MD simulations and force component fitting.

For this screening, we designed and implemented a highly automated and powerful workflow in AiiDA as a plugin called aiida-flipper.^149^ All supercells are passed to the diffusion workflow, which iteratively runs MD simulations with the pinball Hamiltonian and self-consistently refines the pinball coefficients, thereby progressively enhancing the accuracy in determining Li-ion conductivity. Fig. 4 illustrates the details of the workflow. This workflow represents a significant improvement with respect to the previous pinball screening,^117^ as it ensures the convergence of the pinball coefficients and thus improves the quality of the forces.

#### 2.3.2 First-principles MD

**Fig. 5:**
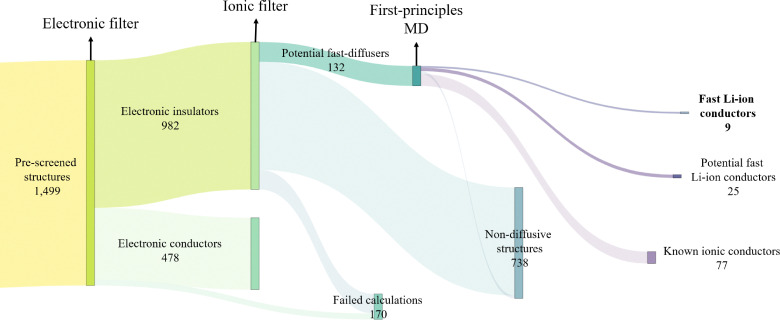
Flowchart of the remaining workflow that only shows electronic structure calculations. Beginning with 1499 pre-screened structures, 9 most promising candidates are identified. Each node represents a filter based on *ab initio* methods that eliminates undesirable structures (indicated by lighter shaded links), while potentially suitable structures advance to the next filter (indicated by darker shaded links). The link thickness corresponds to the number of structures passing through each filter.

As illustrated in Fig. 5, the structures that exhibit high Li-ion diffusivity at 1000 K with the pinball model are subsequently studied with FPMD at the same temperature for 100 ps. However, structures already recognised in the literature as fast ionic conductors, detailed in Section 3.2.1, are excluded to prioritise the discovery of new Li-ion conductors. The structures validated by FPMD as fast ionic conductors are then studied at three lower temperatures: 750 K, 600 K and 500 K for 125 ps, 150 ps and 180 ps respectively. Longer simulation times are chosen to account for comparatively slower equilibration at lower temperatures. These temperatures are selected to be equidistant on the inverse temperature scale. Based on eqn (1), we determine the diffusion coefficient and quantify the statistical variance in diffusivity.^155^ The activation barrier for these structures is estimated from a linear fit of the Arrhenius behaviour^79^ and the error is obtained with Bayesian propagation.^156^ For the most promising structures, we plot Li-ion probability density to better illustrate the Li-ion diffusion channels.

We perform both pinball MD and FPMD on the experimentally reported crystal structures available in crystallographic databases, *i.e.*, idealised ordered structures derived from CIF files. However, real materials often deviate from these ideal structures through point defects, non-stoichiometry, partial occupancies, disorder, grain boundaries, or local structural distortions, all of which can affect ion conduction.^157–160^ In many cases, such deviations may enhance Li mobility, for example, the introduction of Li vacancies or aliovalent substitutions can create additional diffusion pathways or lower migration barriers, potentially transforming a poor conductor in its ideal stoichiometric form into a significantly better conductor in practice.^161^ However, a systematic treatment of such effects in a screening study of this scale is highly non-trivial,^50^ and as such we consider this a promising avenue for future screenings, but ultimately outside the scope of present screening.

## 3 Results and discussion

The pre-screening phase, which does not involve any electronic structure calculations, is illustrated in Fig. 3. Starting with approximately 8000, 9000, and 13 000 experimental structures sourced from COD,^127^ ICSD,^128^ and MPDS^129^ respectively, we extract nearly 23 000 clean CIF files, discarding the unsalvageable ones. All subsequent filters are applied to structures derived from these clean CIF files. We eliminate approximately 10 600 structures with partial occupancies, and from the remaining 12 000 structures with integer atomic occupancies, 5200 are identified as unique using the structure matcher algorithm of pymatgen.^137^ Further filtering removes structures containing unwanted elements and those with unwanted bond lengths, resulting in 1499 structures that advance to the next phase of the screening.

**Fig. 6:**
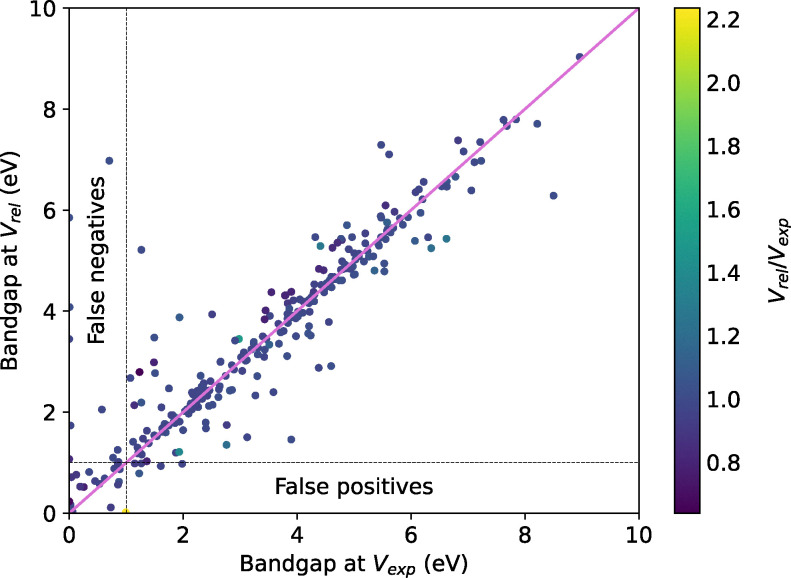
Comparison of band gaps at optimised geometry (*V*_rel_) and experimental geometry (*V*_exp_). For the majority of the structures, the classification as insulators remains unchanged upon relaxation.

We perform single-point calculations on these structures at the level of DFT-PBEsol.^140^ Out of these, 251 calculations fail to converge due to issues in the self-consistent electronic cycle. These are subsequently rerun using the non-linear conjugate gradient method within SIRIUS^162^ enabled Quantum ESPRESSO. Following this, we calculate the band gap for all structures and classify a structure as an electronic insulator if its band gap exceeds 1 eV. Out of the 1499 unique structures, 982 are identified as electronic insulators, and 39 calculations failed, representing the first filter illustrated in Fig. 5. To assess the impact of geometry optimisation on our filtering criterion, we performed additional variable-cell relaxation on 25% of these 1499 structures, of which 316 finished successfully. Fig. 6 compares the band gaps between relaxed and experimental geometries. Our findings indicate that only 5 out of the 316 structures, or less than 2%, are identified as insulators when calculated using experimental geometry instead of performing full variable-cell relaxation, representing false positive results. The reverse scenario, where metallic structures turn into insulators upon relaxation (false negatives), is slightly more common with 10 out of 316 structures. Given that all MD simulations are performed at experimental geometries, we opt not to relax any other structures, considering a less than 2% false positive rate acceptable given the significantly higher computational cost of variable-cell relaxation and the additional failure due to issues in the ionic convergence cycle.

**Fig. 7:**
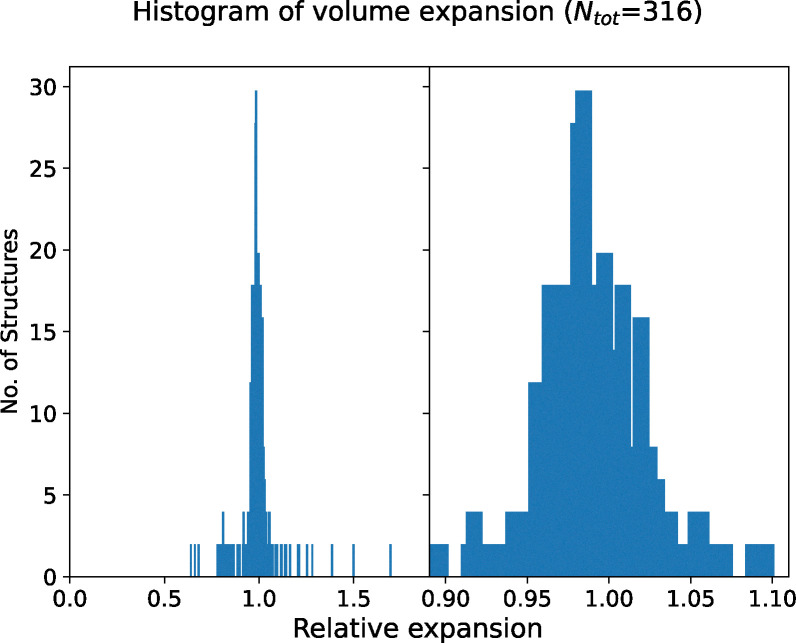
Histogram of relative volume expansion between optimised and experimental geometry at the level of DFT-PBEsol. The left panel displays the complete histogram while the right panel provides a zoomed in view of the range from 0.9 to 1.1.

Fig. 7 presents a histogram of the relative volume change upon geometry optimisation, defined as the optimised volume divided by the experimental volume. Utilising the PBEsol functional, we achieve a narrow and uniform distribution of volume changes, maintaining lattice parameters that more closely match experimental values. This contrasts with the standard PBE functional,^61^ where structures are more likely to exhibit expansion,^163^ as observed by Kahle *et al.*^117^

### 3.1 Pinball MD

**Fig. 8:**
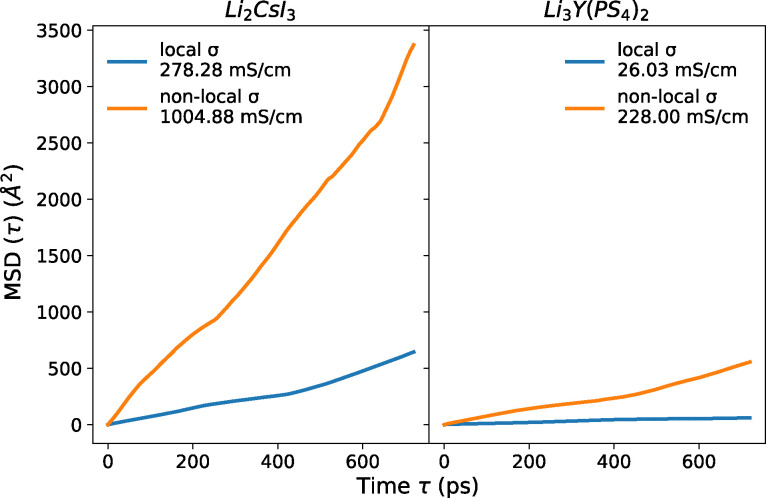
MSD plot of two materials comparing Li-diffusion with and without non-local interactions within the pinball model at 1000 K. Using only local projectors typically leads to an underestimation of Li-ion diffusion. The *r*^2^ coefficient of determination for the pinball forces improves from 0.83 to 0.99 for Li_3_Y(PS_4_)_2_ and from 0.91 to 0.99 for Li_2_CsI_3_ when non-local interactions are included. Based on first-principles simulations Li_3_Y(PS_4_)_2_ and Li_2_CsI_3_ show ionic conductivities of 2.16 mS cm^−1^ at 300 K^164^ and 0.22 mS cm^−1^ at 500 K^117^ respectively.

All the MD simulations are performed on the supercells generated from the 982 insulators identified in the previous step. To evaluate the significance of including non-local interactions within the pinball model, we conducted tests on a few systems both with and without non-local interactions. The MSD plots of this comparison, shown in Fig. 8, reveal that using only local projectors typically leads to an underestimation of Li-ion diffusion. Consequently, we opt to include it in our screening, despite the higher computational cost.

**Fig. 9:**
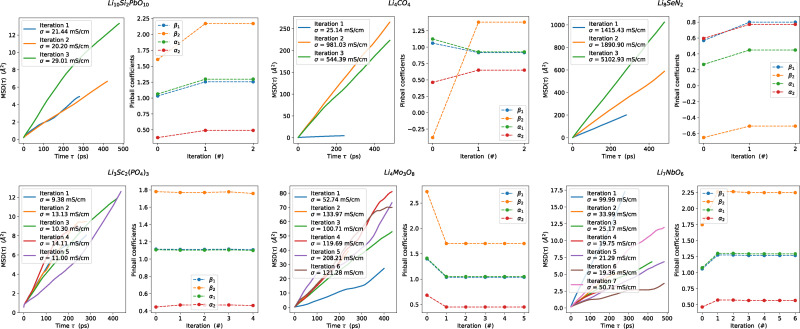
Self-consistent iterations for the MSD plots of Li, along with the convergence of pinball coefficients for several fast Li-ion conductors. The zeroth pinball coefficients are derived by fitting DFT and pinball forces on randomly rattled structures, which are then used to perform the first MD iteration. Force fitting is subsequently performed on configurations obtained from the first MD iteration to obtain the first pinball coefficients, which are then used in the second MD iteration. For most structures pinball coefficients converge after two of these self-consistent iterations, with the estimate of Li-diffusion remaining largely unchanged, illustrating the need for a self-consistent cycle. For a select few structures, additional iterations are performed after the convergence of the pinball parameters to verify that no divergence occurred in subsequent steps, ensuring the robustness of the workflow. We attribute the slight change in dynamics in some of the MD simulations to the inherent stochasticity of the thermostat that is used.^151^

Next, we derive an initial estimate of the pinball coefficients through the linear regression of forces calculated at both DFT and pinball levels for all supercells. The quality of these coefficients is evaluated using the *r*^2^ correlation between DFT and pinball forces. We ensure that the *r*^2^ correlation for the converged pinball coefficients exceeds 0.95, with the majority of cases exceeding 0.99. If this criterion is not met, additional self-consistent pinball MD iterations are performed, allowing for the extraction of further uncorrelated configurations from these extended MD simulations. These serve as additional data points for improving the fit until full convergence is achieved, as indicated by stable pinball coefficients and an *r*^2^ value approaching 1. Out of 982 structures, we achieve convergence for 914, with failures occurring due to issues in the self-consistent electronic cycle when calculating DFT forces. An additional 63 structures failed the pinball MD simulations due to drift in the constant of motion, leading to 851 structures with a final iteration of the pinball MD run with converged coefficients and a total simulation time of 22.1 µs. As illustrated in Fig. 9, the pinball coefficients readily converge for most structures. Based on the slope of the MSD plot from the final MD iteration and eqn (2), we estimate Li-ion conductivity. An ionic conductivity of 1 mS cm^−1^ at 1000 K is chosen as the threshold to categorise potential fast ionic conductors at the pinball level.

At the end of this process, 132 structures are identified for further studies using first-principles calculations. Of these 132 structures, 49 originate from the newly considered MPDS repository, while the remaining structures were present in the overlapping datasets in the previous screening.^117^ We attribute the inclusion of non-local interactions within the pinball model along with the self-consistent fitting of the pinball coefficients as the main reasons for the increased predictive power of current workflow over the previous screening.

### 3.2 First-principles MD

We classify the 132 structures obtained from the self-consistent pinball workflow into four categories: (1) structures already identified in the literature as Li-ion conductors, (2) structures that do not exhibit diffusion within FPMD, (3) structures that show negligible diffusion at lower temperatures but may still be of interest, and (4) fast Li-ion conductors.

#### 3.2.1 Known Li-ion conductors

For all the structures that are identified as fast Li-ion conductors using the pinball model, we conduct an extensive literature review to assess those that have already been studied and reported as fast ion conductors. Out of these 132 structures, we rediscover 77 known Li-ion conductors and as such we exclude them from FPMD investigations.

In the following short review, we report these 77 structures and their current use case if applicable. Li_2_Ti_6_O_13_ is a known ionic conductor^165^ and was recently proposed as a cathode material,^166^ while sodium substituted Li_2_Ti_6_O_13_ is used as an intercalation anode.^167^ Li_7_P_3_S_11_ is a well-known superionic conductor.^168,169^ Li_2_TeO_4_ is a known superionic conductor^170^ and was proposed as an electrode material.^171^ Li_6_NBr_3_ was experimentally shown to be a fast ionic conductor,^172^ but worse than the well known Li_3_N,^173,174^ which we also identified. LiI is a well known ionic conductor,^175^ while LiBr and LiCl show negligible Li-ion conduction without doping.^176^ Li_2_Se is used as a cathode material^177^ and also as an interface material.^178^ Li_3_BN_2_ is a well known fast ionic conductor.^179^ Li_3_BS_3_ was reported in the computational screening by Laskowski *et al.*,^180^ despite an earlier computational study^181^ that proposed it as a potential ionic conductor. Li_4_SnSe_4_ is a known ionic conductor.^182^ Li_2_SiN_2_ is used as an anode material^183^ and Li anode coatings are used to increase electrochemical stability.^184^ Li_2_SiP_2_ is a known ionic conductor^185^ and was proposed as a potential solid-state electrolyte material.^186^ Li_2_SiS_3_ is a known ionic conductor.^187,188^ LiBF_4_ has been used as non-aqueous electrolyte for two decades.^189^ Li_3_BrO is a known superionic conductor.^190,191^ Li_3_Y(PS_4_)_2_ was proposed in a computational study with very high ionic conductivity.^164^ Li_3_PS_4_ is a known ionic conductor^192^ and has been engineered with much better properties in the past decade.^193^ Li_4_PN_3_ was recently discovered using first principles simulations.^194,195^ Li_5_AlS_4_ was experimentally reported to have low ionic conductivity at room temperature,^196^ but is otherwise known in the argyrodite family.^197^ Li_5_NCl_2_ has been known to be an ionic conductor for a long time,^198^ but was recently studied in greater detail by Landgraf *et al.*^199^ Li_7_BiO_6_ has been known for a long time to be an ionic conductor.^200^ LiGa(SeO_3_)_2_ was proposed recently by Jun *et al.*^118^ LiHf_2_(PO_4_)_3_ is a known ionic conductor,^201,202^ but Al substituted Li_1+*x*_Al_*x*_Hf_2−*x*_(PO_4_)_3_ showed more promise.^203^ LiInS_2_ is a known ionic conductor^204^ and was recently studied within the LiXS_2_ family as a cathode material.^205^ LiS^206^ is a part of the Li–S battery system, while Li_2_S is used as a cathode material.^207^ Li_3_InO_3_ is a known ionic conductor.^208^ LiZnPS_4_ is a poor ionic conductor, but with defect engineering shows more promise.^209,210^ LiTi_2_(PO_4_)_3_ is used as a cathode material in aqueous batteries especially when doped as LiMn_*x*_Ti_2−*x*_(PO_4_)_3_;^211,212^ further doping has yielded promising results as an SSE.^213^ LiNbO_3_ is used as a coating on cathode materials^214^ and also as an anode material in Li-ion capacitors.^215^ LiAlCl_4_ is not well studied, despite being a known ionic conductor for a long time.^216^ Li_2_O is a well known ionic conductor.^73,217^ Li_2_Mo_4_O_13_ was recently proposed as an anode material.^218^ LiSn_2_(PO_4_)_3_ is a well known anode material with various different synthesis methods.^219–221^ Li_4_SnS_4_ is a known ionic conductor.^222^ Li_9_S_3_N is a known ionic conductor^223^ and was proposed as a barrier coating between electrolyte and the Li metal anode.^224^ Li_4_GeS_4_ is a well known ionic conductor.^225^ LiCF_3_SO_3_ is a known ionic conductor along with sodium, caesium and rubidium substitutes.^226,227^ Li_5_NBr_2_ and Li_10_N_3_Br were investigated recently in the halogen-nitride system Li_3*a*+*b*_N_*a*_X_*b*_, with Li_10_N_3_Br found to be an excellent ionic conductor.^228^ Li_3_In_2_(PO_4_)_3_ is a known superionic conductor.^229^ Li_2_B_6_O_9_F_2_ is a known ionic conductor.^230^ Li_2_SrTa_2_O_7_ is a known ionic conductor but other substitution compounds are more promising.^231^ Li_7_SbO_6_ is a known ionic conductor.^232^ Besides this, Kahle *et al.*^117^ proposed the following as fast Li-ion conductors: Li_5_Cl_3_O, Li_7_TaO_6_, LiGaI_4_, LiGaBr_3_, and Li_3_CsCl_4_ and Li_2_CsI_3_, which are theoretical structures,^233^ Li_2_WO_4_, which is used to improve conductivity of other materials either as a solid mixture^234^ or in solid solution,^235^ and LiAlSiO_4_, whose suitability was systematically studied with Al doping by Ryu *et al.*^236^ Last, FPMD simulations performed by Kahle *et al.*^117^ showed insignificant diffusion in the following structures at lower temperatures: LiAlSe_2_, Li_4_Re_6_S_11_, LiPO_3_, Li_3_Sc_2_(PO_4_)_3_, Li_4_P_2_O_7_, (LiI)_2_Li_3_SbS_3_, Li_6_PS_5_I, Li_5_P(S_2_Cl)_2_, Li_3_P_7_, Li_3_SbS_3_, Li_2_B_3_O_4_F_3_, Li_2_Mg_2_(SO_4_)_3_, Li_3_AsS_3_, Li_2_Si_2_O_5_, Li_2_NaB(PO_4_)_2_, Li_6_Y(BO_3_)_3_, and LiAuF_4_.

#### 3.2.2 Non-diffusive structures

We find 18 materials that do not exhibit Li-ion diffusion in our FPMD simulations at 1000 K. This absence of diffusion suggests that they are unlikely to demonstrate Li-ion conductivity in experiments unless significantly doped. The materials in question include oxides, halides, sulphides and selenides, all of which are detailed in Table 1 along with their respective experimental references. The MSD plots of these materials are provided in the SI, Section S3.

**Table 1:** The structures that were found to be conducting at the level of pinballs, but show insignificant diffusion with FPMD at 1000 K. Consequently, these were not studied at lower temperatures. We report their stoichiometry, the repository and identifier from where they originated along with the corresponding experimental reference. Refer to Section S2 of the SI for the MSD plots at 1000 K

Structure	Database	Database-id
Li_2_Te_2_O_5_	ICSD	26451, 26452^237^
Li_2_CsCl_3_	MPDS	S1022277^238^
LiKSe	ICSD	67277^239^
LiYS_2_	MPDS	S537670^240^
LiInSe_2_	MPDS	S1214509^241^
LiAlS_2_	ICSD	608360^242^
LiLuS_2_	MPDS	S307222^243^
Li_7_Te_3_O_9_F	MPDS	S1533619^244^
Li_5_SiP_3_	MPDS	S1145472^245^
Li_6_RbBiO_6_	MPDS	S1408313^246^
LiAuF_6_	MPDS	S1904723^247^
Li_3_Na_3_Ga_2_F_12_	MPDS	S1836948^248^
LiZrS_2_	MPDS	S301115^249^
Li_2_CdSnSe_4_	MPDS	S1952801^250^
LiBa_4_Ga_5_Se_12_	MPDS	S1021504^251^
Li_3_Na_3_Rh_2_F_12_	MPDS	S307582^252^
Li_2_HgO_2_	MPDS	S1702887^253^
Li_2_Ca_2_Ta_3_O_10_	ICSD	88497^254^

#### 3.2.3 Potential fast Li-ion conductors

We have identified 25 structures that exhibit significant diffusion at 1000 K in our FPMD simulations, but do not display the same behaviour at lower temperatures. Of these 25, 7 originate from the newly considered MPDS dataset with Li_10_BrN_3_ present in both MPDS and ICSD. These structures are listed in Table 2, ranked according to their likelihood of exhibiting diffusion at lower temperatures. Their MSD plots at all four temperatures are provided in the SI, Section S2. It is important to emphasise that these structures may indeed show significant diffusion at lower temperatures under experimental conditions. The inability of our simulations to detect diffusion at these temperatures is likely due to the prohibitively long simulation times required to observe Li-ion hoping at lower temperatures. For instance, Materzanini *et al.* reported ionic conductivities of 28 mS cm^−1^ and 6 mS cm^−1^ for tetragonal-LGPO at 500 K and orthorhombic-LGPO at 600 K,^84^ respectively, corresponding to MSDs of approximately 0.04 Å^2^ ps^−1^ and 0.005 Å^2^ ps^−1^. This indicates that in the tetragonal phase, a Li-ion travels an average distance of 1 Å within 25 ps, whereas in the orthorhombic phase, it would require 200 ps to cover the same distance. Similarly, cubic-LLZO exhibits an ionic conductivity of 20 mS cm^−1^, which despite being 10 000 times greater than that of the tetragonal phase,^255^ corresponding to an MSD of 0.01 Å^2^ ps^−1^. Therefore, simulations with durations of 100–200 ps are insufficient to accurately resolve diffusion in such systems. Consequently, the activation barriers that we report may not be entirely accurate due to lack of sufficient statistics at lower temperatures. Similar to the previous section, we observe materials that include oxides, halides, phosphides and additional nitrides.

**Table 2:** The structures that show significant diffusion at 1000 K but not at lower temperatures with FPMD. We report their stoichiometry, the repository and identifier from where they originated along with the experimental reference, band gap at the level of DFT-PBEsol, and ionic conductivity at 1000 K with pinball MD and FPMD. Refer to Section S2 of the SI for the MSD plots at all four temperatures

Structure	Database	Database-id	Bandgap	Ionic conductivity	Ionic conductivity
			DFT-PBEsol (eV)	Pinball (mS cm^−1^)	FPMD (mS cm^−1^)
Li_2_BeF_4_	MPDS	S1935520^256,257^	7.49	10	1822
Li_2_Ti_4_O_9_	MPDS	S559372^258^	3.2	1042	1348
LiY_2_Ti_2_S_2_O_5_	COD	4124533^259^	1.25	74	1251
Li_10_BrN_3_	MPDS	S1614518^260^	1.79	4247	886
Li_2_Cs_3_Br_5_	ICSD	245978^233^	3.79	106	594
Li_8_SeN_2_	MPDS	S1931016^261^	1.88	467	588
Li_8_TeN_2_	MPDS	S1931019^261^	2.28	214	446
LiCF_3_SO_3_	ICSD	110018^262^	6.78	40	384
Li_2_ZnBr_4_	COD	1517836^263^	3.75	8	357
LiBeP	ICSD	670551,^264^ 42037^265^	2.75	18	356
Li_5_Br_2_N	ICSD	78836^260^	2.29	1351	344
Li_10_Si_2_PbO_10_	ICSD	78326^266^	2.86	29	342
Li_2_ZnGeSe_4_	COD	7031897^267^	1.89	63	291
LiCs_2_I_3_	ICSD	245984^233^	3.41	1410	280
LiSr_2_Br_5_	MPDS	S1941469^268^	3.53	176	263
LiGaSe_2_	COD	1531591^269^	2.23	5	173
LiP_7_	ICSD	23621^270^	1.56	11	133
LiMoPO_6_	COD	7701361^271^	2.52	8	132
LiY(MoO_4_)_2_	COD	1008103^272,273^	3.22	660	68
Li_10_B_14_Cl_2_O_25_	MPDS	S1803375^274^	6.30	13	65
Li_2_P_2_PdO_7_	COD	1000333^275^	1.39	513	48^5^
Li_2_B_3_PO_8_	MPDS	S1614518^276^	5.49	45	41
Li_2_B_2_Se_5_	COD	1510746^277^	1.84	84	20
Li_8_Bi_2_(MoO_4_)_7_	ICSD	54021^278^	2.95	7	12
Li_3_AuS_2_	COD	4319430^274^	1.86	895	8

##### 3.2.3.1 Li_10_Si_2_PbO_10_

**Fig. 10:**
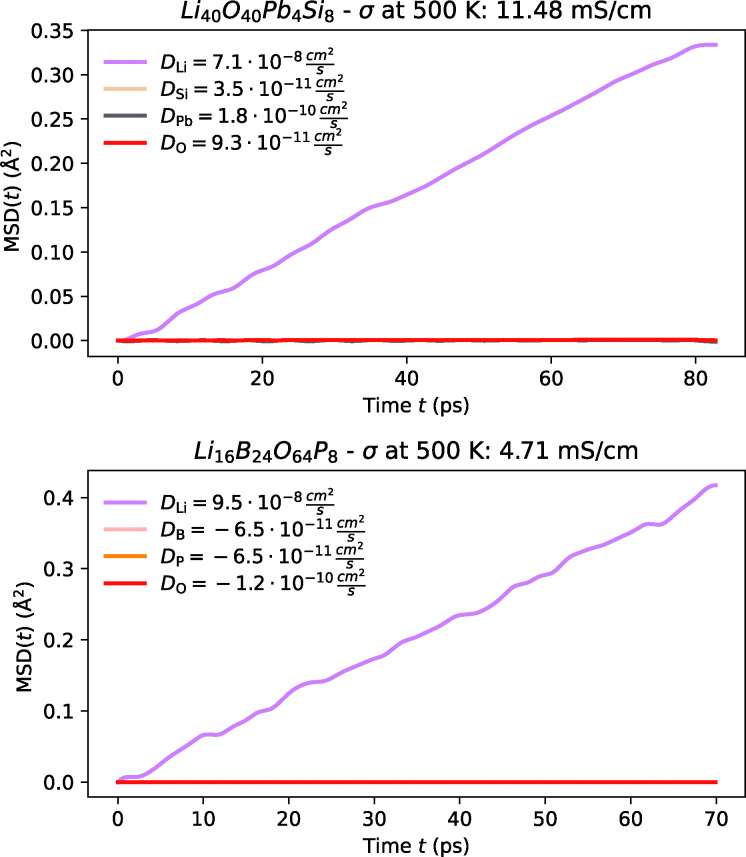
MSD plot of Li along with host-lattice species of Li_10_Si_2_PbO_10_ and Li_2_B_3_PO_8_ at 500 K from FPMD.

Originally synthesised in 1994 by Brandes *et al.*,^266^ this material has received limited attention, particularly in the context of fast Li-ion conduction. Lead-silicate glasses, including this compound, are known for diverse optical properties, such as transparency, refractive index, colouration, electrical conductivity, and chemical durability^279^ and have found application in areas such as the monitoring of radioactive materials.^280^ However, their potential as solid-state electrolytes remains largely unexplored. In our simulations, Li_10_Si_2_PbO_10_ displays Li-ion diffusion at lower temperatures, as shown in Fig. 10; yet, its relatively high activation energy of 0.35 eV results in an estimated room-temperature ionic conductivity of less than 0.1 mS cm^−1^, limiting its viability as a potential electrolyte material.

##### 3.2.3.2 Li_2_B_3_PO_8_

Synthesised relatively recently in 2014 by Hasegawa *et al.*,^276^ this material has yet to receive significant attention, particularly in the context of Li-ion batteries. Borophosphates of this kind have primarily been investigated in the semiconductor industry for their magnetic coupling mechanisms, optical characteristics, and catalytic behaviour.^281,282^ Similar to Li_10_Si_2_PbO_10_, our simulations are able to resolve Li-ion diffusion at lower temperatures for Li_2_B_3_PO_8_, as shown in Fig. 10; the relatively high activation energy of 0.28 eV renders it less suitable for room-temperature applications, where the estimated ionic conductivity falls well below 0.1 mS cm^−1^.

##### 3.2.3.3 Li_2_BeF_4_

This material was first synthesised in 1952 by Novoselova *et al.*^256^ and exhibits one of the highest Li-diffusion at elevated temperatures. However, due to the inability to resolve diffusion at lower temperatures, accurately quantifying its activation barrier remains challenging. We anticipate that with sufficiently long simulations, on the order of several nanoseconds, it would be possible to quantify diffusion at lower temperatures as well, making this material an excellent candidate for further investigation using machine learning techniques. Furthermore, it has notably been used as a coolant in nuclear reactors,^283^ highlighting the established interest in its synthesisability within experimental settings. Given these factors and the toxic nature of beryllium, it remains an interesting case study.

##### 3.2.3.4 Li_8_SeN_2_ and Li_8_TeN_2_

Both selenium and tellurium nitrides, which demonstrate excellent Li-ion diffusion at higher temperatures, were first synthesised in 2010 by Bräuling *et al.*^261^ They are three-dimensional diffusers, but at lower temperatures they do not show high diffusion. Furthermore, nitrides are generally among the most challenging materials to process due to relatively high-temperature synthesis routes;^284^ we refrain from classifying them as the most promising candidates within this screening.

#### 3.2.4 Fast Li-ion conductors

**Fig. 11:**
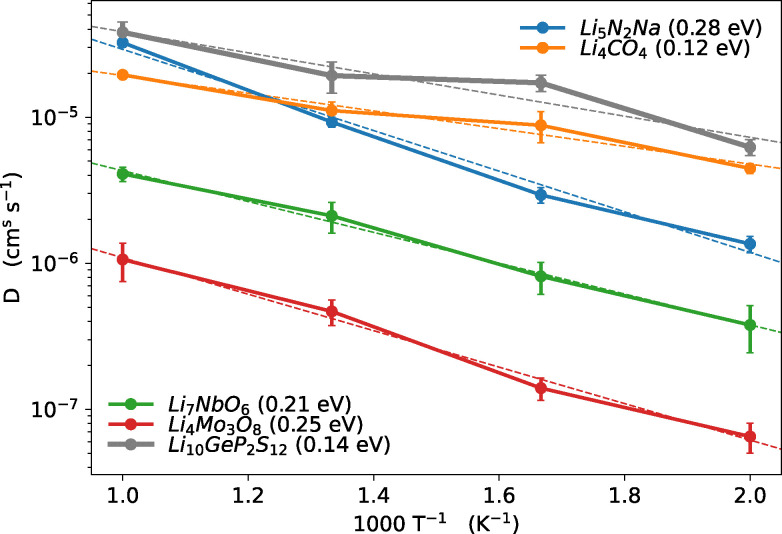
Diffusion coefficients derived from our FPMD simulations for the most promising oxides and nitrides. The dashed line represents the best-fit line, with the slope corresponding to the activation barriers, indicated in brackets (eV). We additionally show LGPS for comparison, with data taken from the work of Kahle *et al.*^117^

**Fig. 12:**
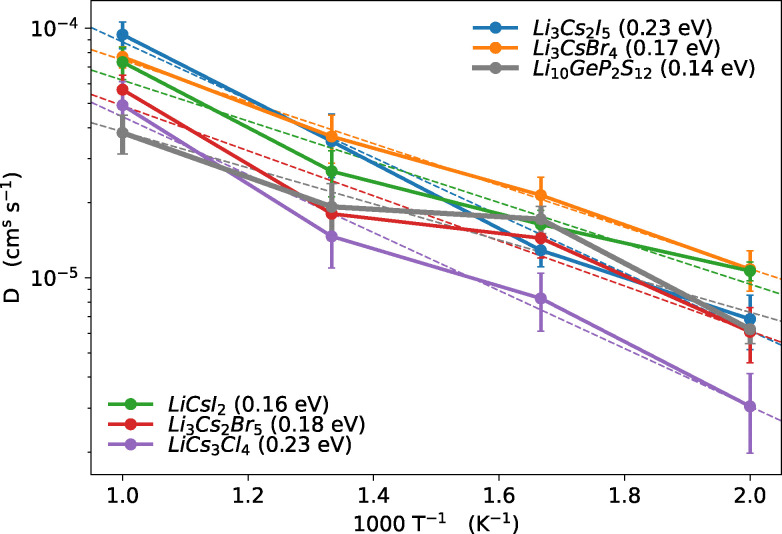
Diffusion coefficients derived from our FPMD simulations for the most promising halides. The dashed line represents the best-fit line, with the slope corresponding to the activation barriers, indicated in brackets (eV). We additionally show LGPS for comparison, with data taken from the work of Kahle *et al.*^117^

In this section we discuss the most promising materials identified as candidates for solid-state electrolytes. These materials are of particular interest due to their potential applications, characterised by their fast ionic conduction, which allows us to resolve Li-ion diffusion even at low temperatures and estimate activation barriers, as illustrated in Fig. 11 and 12. For comparison, we also include tetragonal-LGPS, with data taken from the work of Kahle *et al.*^117^ Although the materials identified in our screening, with the exception of the Cs-containing halides, do not exhibit ionic conductivities as high as LGPS, their excellent activation energies suggest that they could perform very well as conductors at room temperature. We list these 9 materials in Table 3, along with details of their provenance, band gap, ionic conductivity at 1000 K and projected conductivity at room temperature, and activation barrier. Of these 9, Li_4_Mo_3_O_8_ originates solely from the newly considered MPDS dataset while Li_7_NbO_6_ is also present in ICSD.

**Table 3:** The most promising structures that were found to be conducting with FPMD at lower temperatures. We report their stoichiometry, the repository and identifier from where they originated along with the experimental reference, band gap at the level of DFT-PBEsol, ionic conductivity at 500 K, 750 K and 1000 K, estimated activation energy using the Arrhenius plot. As a comparison LGPS and LLZO have an ionic conductivity of 1101 mS cm^−1^ (ref. 117) and 295 mS cm^−1^ (ref. 285) respectively at 1000 K

Structure	Database	Database-id	Bandgap	Ionic conductivity	Ionic conductivity	Ionic conductivity	Activation energy
			DFT-PBEsol (eV)	at 500 K (mS cm^−1^)	at 750 K (mS cm^−1^)	at 1000 K (mS cm^−1^)	(eV)
Li_4_CO_4_	ICSD	245389^286^	5.26	235	551	726	0.15
LiCsI_2_	ICSD	245986^287,288^	3.32	203	340	698	0.16
Li_3_CsBr_4_	ICSD	245982^233^	3.73	456	1035	1616	0.17
Li_3_Cs_2_Br_5_	ICSD	245980^233^	3.90	181	358	844	0.18
Li_7_NbO_6_	MPDS	S1818764^289^	3.58	77	288	418	0.21
Li_3_Cs_2_I_5_	ICSD	245987^290,291^	3.43	161	554	1112	0.23
LiCs_3_Cl_4_	ICSD	245969^233,292^	4.38	35	112	283	0.23
Li_4_Mo_3_O_8_	MPDS	S1614518^293^	1.17	6	32	54	0.25
Li_5_NaN_2_	ICSD	92313^294^	1.49	279	1268	3609	0.28

##### 3.2.4.1 Li_7_NbO_6_

**Fig. 13:**
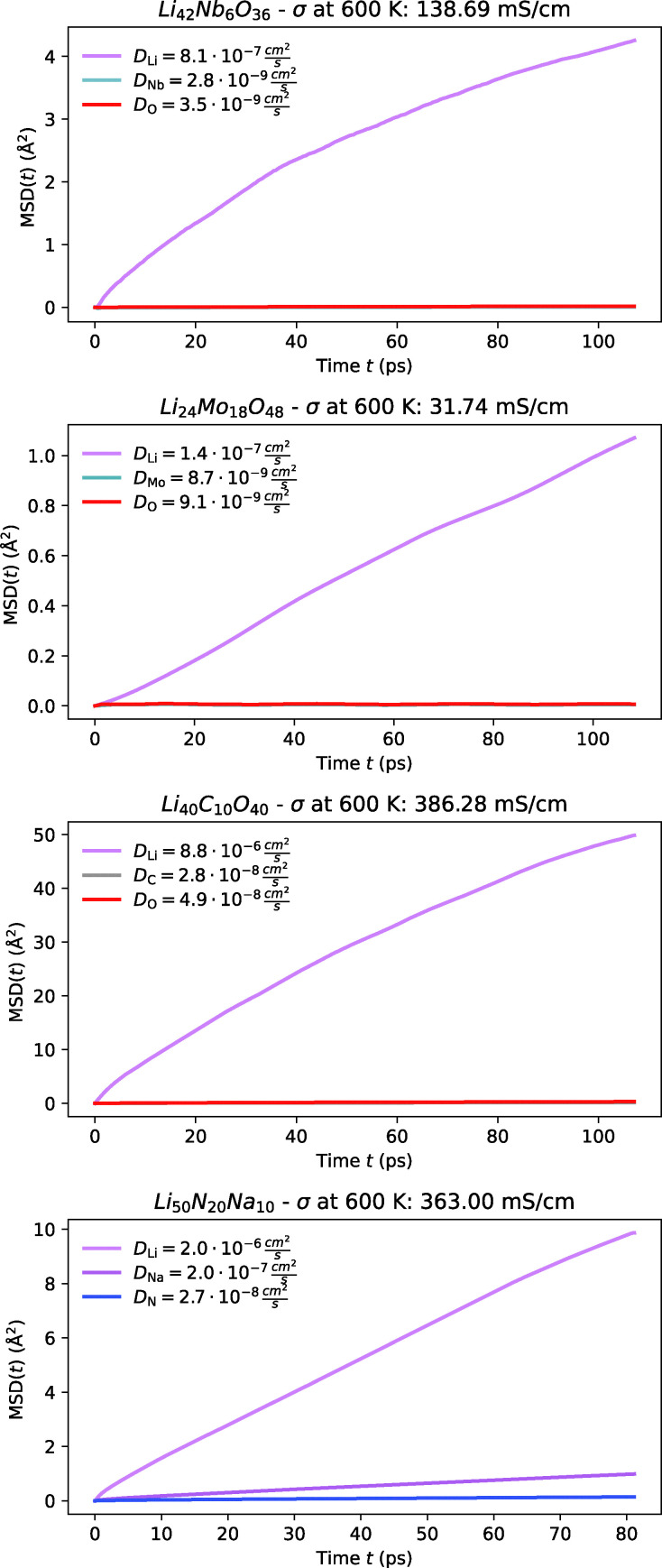
MSD plot of Li along with host-lattice species of the oxides Li_7_NbO_6_, Li_4_Mo_3_O_8_, and Li_4_CO_4_, and the nitride Li_5_NaN_2_ at 600 K from FPMD.

First synthesised in 1969,^289^ this material has been the subject of multiple experimental studies;^232,295^ however it has never been investigated as a Li-ion conductor until He *et al.* proposed Li_7_NbO_6_ as a potential ionic conductor.^296^ Subsequent computational work by Feng *et al.* reported a low ionic conductivity of 0.008 mS cm^−1^ along with a significantly higher activation barrier and lower diffusion than our predictions,^297^ as illustrated in Fig. 13. We note, however, that our study and that of Feng *et al.* address two distinct structural phases of the same composition. Our calculations indicate that the phase considered in the present work is higher in total energy by approximately 1 eV per formula unit relative to the phase reported by Feng *et al.*, suggesting that it is metastable within our DFT framework, despite having been reported experimentally. This makes the Li_7_NbO_6_ system particularly interesting from a materials-design perspective, since it raises the possibility that the high-conductivity phase identified here could be stabilised under suitable synthesis conditions or through chemical substitution. In this context, Feng *et al.* also investigated tungsten doping and found an enhancement of the room temperature conductivity to 0.28 mS cm^−1^, which remains an order of magnitude lower than our estimated value of 5 mS cm^−1^. Nevertheless, these findings support doping as an effective strategy to further improve the ionic conductivity. Given these promising properties and the substantial experimental background already established, we propose that Li_7_NbO_6_ holds significant potential as an excellent electrolyte for future applications.

##### 3.2.4.2 Li_4_Mo_3_O_8_

This molybdenum oxide exhibits high Li-ion conductivity, as illustrated in Fig. 13. The yttrium-doped variant was first synthesised in 1980, while the undoped form was synthesised in 1999. Our FPMD simulations indicate that both materials possess low activation barriers, with the yttrium-doped version performing slightly better. Based on the activation energies, we estimate the ionic conductivities at room temperature to be 0.2 mS cm^−1^. We strongly recommend further experimental studies to validate these findings and confirm the potential as solid-state electrolytes.

##### 3.2.4.3 Li_5_NaN_2_

The well known Li_3_N was first proposed in 1935^298^ and has since spawned a broad class of Li-ion conductors that continue to attract attention today.^299^ While studying Li_3_N in 2000, Schön *et al.* proposed the metastable Li_5_NaN_2_, as a derivative of Li_3_N, with relatively low formation energy.^294^ Our calculations are able to resolve Li-ion diffusion at lower temperatures as shown in Fig. 13 and indicate a relatively higher activation barrier of 0.28 eV, which corresponds to an estimated room-temperature ionic conductivity of 4 mS cm^−1^. Given the success of doping in enhancing the conductivity of Li_3_N,^300,301^ we are optimistic that similar strategies could improve the performance of Li_5_NaN_2_, making it a promising candidate. However, a notable challenge is the concurrent diffusion of Na-ions, highlighting the need for targeted compositional or structural engineering to restrict Na-ion mobility.

##### 3.2.4.4 Li_4_CO_4_

We examined this material in four distinct crystal structures with the same stoichiometry, all of which exhibited excellent Li-ion diffusion. However, this material remains a theoretical structure that exists at high pressure and appears to simply be a variant of Li-doped carbonates, which may decompose at ambient temperature and pressure.^286^ Given these uncertainties, we are cautious about its potential as an electrolyte. Despite its low activation barrier, we have opted not to list it as a promising candidate until further validation can be conducted, and it is established that these materials can exist at normal temperature and pressure without decomposing into simple carbonates. Based on the activation energy, we estimate the ionic conductivity at room temperature to be 37 mS cm^−1^.

##### 3.2.4.5 Cs-containing halides

**Fig. 14:**
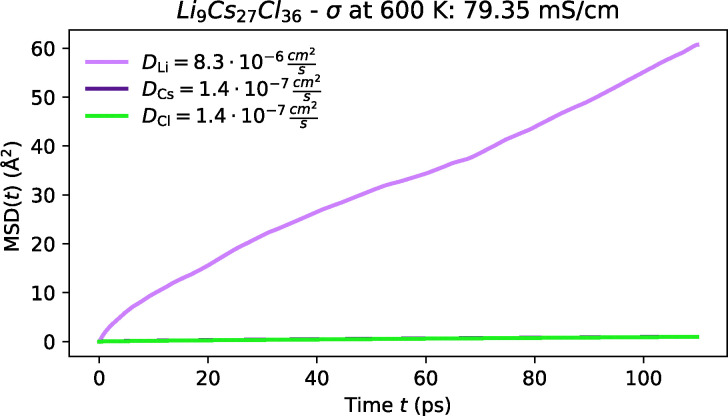
MSD plot of Li along with host-lattice species in Cs-containing chloride at 600 K from FPMD.

**Fig. 15:**
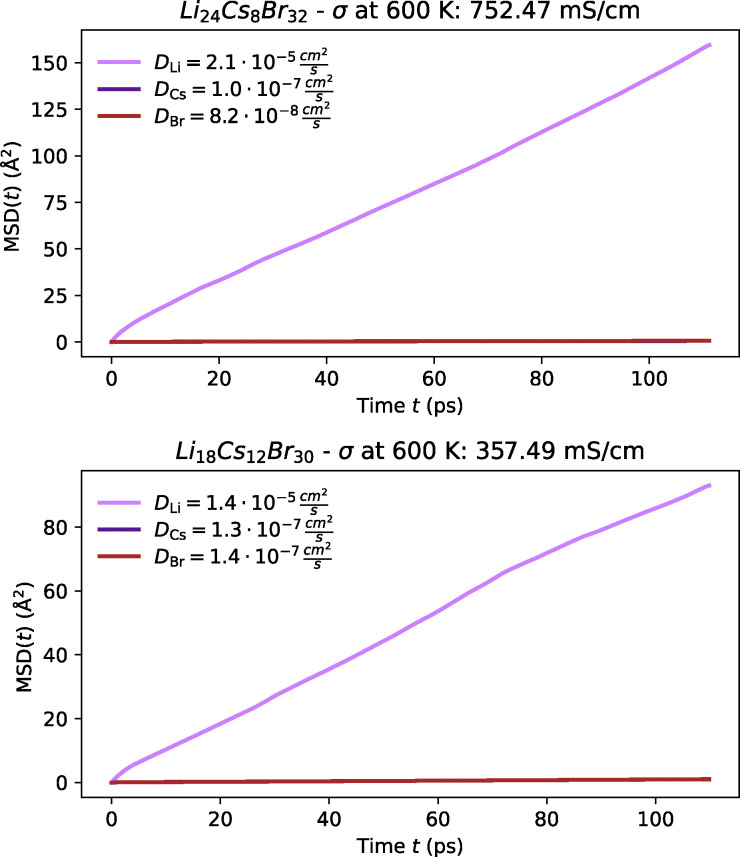
MSD plot of Li along with host-lattice species in Cs-containing bromides at 600 K from FPMD.

**Fig. 16:**
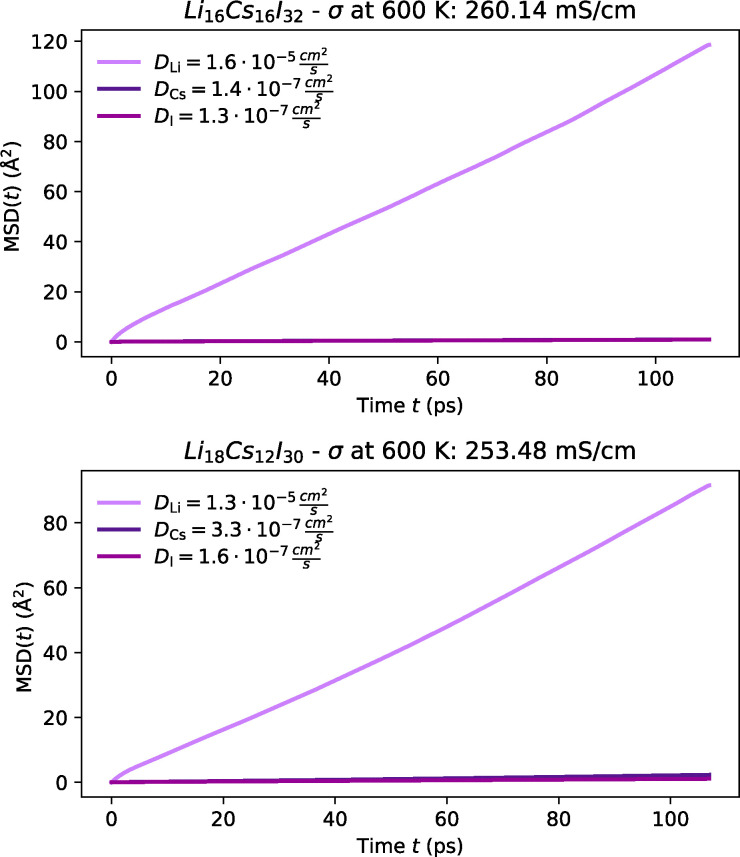
MSD plot of Li along with host-lattice species in Cs-containing iodides at 600 K from FPMD.

Amongst the most promising materials we identify are Li_3_CsBr_4_, Li_3_Cs_2_Br_5_, LiCs_3_Cl_4_, LiCsI_2_ and Li_3_Cs_2_I_5_, some of which were first proposed by Pentin *et al.* using *ab initio* methods.^233^ Each of these materials demonstrates high ionic conductivity as shown in Fig. 14–16; and low activation barrier ranging from 0.15 to 0.25 eV as illustrated in Fig. 12. Although experimental validation for these materials is still pending, their synthesis appears to be feasible. Most notably, LiCsI_2_,^288^ Li_3_Cs_2_I_5_^291^ and Li_2_CsI_3_^290^ (which was also proposed by Kahle *et al.*^117^) have all been successfully synthesised, and Li_3_Cs_2_Br_5_ may also be synthesised following a similar approach to Li_3_Cs_2_I_5_. While these compounds have not yet been explored as ionic conductors, our screening suggests significant potential for future experimental validation. Though the synthesisability of the other Cs–Li–halides remains uncertain, they may depend on methodologies similar to those used for this ternary system.^290^ Given these considerations, we hesitate from designating these materials as the top candidates within this screening, pending further experimental investigations. However, it is important to emphasise that despite these uncertainties, this system represents a promising avenue for further exploration and warrants both experimental and theoretical pursuits.

**Fig. 17:**
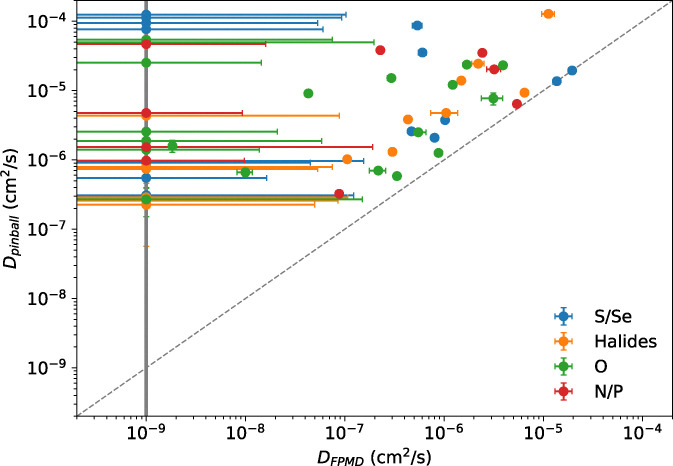
Comparison of diffusion coefficients at 1000 K, obtained with the pinball model and FPMD, categorised by the predominant anion. The bold-grey line represents the threshold below which MSD convergence cannot be achieved with FPMD, serving as the lower bound for diffusion. The dashed-grey line denotes the identity line, with all of the structures lying on or above it, suggesting that the pinball model typically overestimates the actual ionic conductivity.

As a final validation of the pinball model, we compare the diffusion coefficients obtained from FPMD and pinball MD for the structures discussed in Sections 3.2.2–3.2.4, as shown in Fig. 17. At first glance, first-principles diffusion is not quantitatively reproduced by the pinball model, which generally tends to overestimate the diffusion coefficient. This systematic bias is consistent with the underlying approximations of the method, in particular the frozen host-lattice treatment, which neglects lattice dynamics and the feedback of the mobile Li ions on the host sublattice.^119^ While the empirically observed false-positive rate among the top candidates (that were subsequently validated by FPMD) is low, the same cannot be said about the false-negatives, since there are examples of materials that are classified as non-conducting at the level of pinball MD that might be conducting,^302^ which underscores the difficulty in quantifying the true predictive power of the pinball model. A more thorough discussion of false positives and negatives can be found in ref. 117. Considering that the pinball workflow successfully rediscovered 77 known ionic conductors, the number of false positives appears to be low, setting an upper bound of ∼85% on the overall predictive rate of our workflow.

## 4 Conclusions and outlook

We conducted a high-throughput computational screening of over 30 000 lithium containing experimental structures sourced from the MPDS, ICSD, and COD repositories. Through the application of several structural filters, we identified approximately 1500 unique crystal structures suitable for electronic structure calculations. We determined the band gaps for these structures at the level of DFT with the PBEsol functional and identified nearly 1000 as electronic insulators. To investigate Li-ion diffusion, we implemented a self-consistent MD workflow in AiiDA, utilising the computationally efficient and highly accurate pinball model. From these simulations, we identified 132 fast Li-ion diffusers, 77 of which were previously recognised in the literature as Li-ion conductors. The remaining 55 materials were further examined using full first-principles MD simulations, leading to the discovery of seven promising materials, including the oxides LiY(MoO_4_)_2_, Li_4_Mo_3_O_8_ and Li_7_NbO_6_, the nitrides Li_8_SeN_2_ and Li_8_TeN_2_, and Cs-containing iodides LiCsI_2_ and Li_3_Cs_2_I_5_. These materials demonstrated excellent activation barriers and Li-ion diffusion near room temperature comparable to or exceeding that of LGPS, a well-known Li-ion superconductor. However, it is important to note that this estimation is based on the extrapolation of the Arrhenius plot, where a change of slope is possible. Additionally, we identified five other materials with similar levels of ionic conductivity, although their synthesisability remains uncertain. Furthermore, we identified 25 potential fast Li-ion conductors, including Li_2_BeF_4_ and Li_8_SeN_2_, that exhibit high Li-ion diffusion at elevated temperatures. However, due to the limited timescales accessible to first-principles MD simulations, we were unable to resolve their diffusion behaviour at lower temperatures. These materials may be promising candidates for further study using machine learning techniques, which could enable more extended simulations at lower temperatures.

Finally, we expect that the extensive first-principles data generated through this study will play a crucial role in training the next generation of machine learning interatomic potentials (MLIPs). To facilitate this, we have made all our first-principles data, along with comprehensive provenance, publicly available on the open-source Materials Cloud archive platform.^303^ This dataset could be particularly instrumental in developing a “universal-Li” MLIP, which has the potential to unlock new and intriguing systems in the future and serve as a foundational tool for the study of next-generation solid-state Li-ion batteries.

## Author contributions

T. S. T.: conceptualisation, data curation, formal analysis, methodology, software, validation, visualisation, writing – original draft; L. E.: methodology, software; N. M.: funding acquisition, project administration, supervision; all authors: writing – review and editing.

## Conflicts of interest

There are no conflicts to declare.

## Supplementary Material

EE-019-D5EE07336G-s001

## Data Availability

Data for this article, including first-principles molecular dynamics trajectories and input crystal structures are available at Materials Cloud Archive at: https://doi.org/10.24435/materialscloud:xm-46. The code for running all the calculations can be found at: https://github.com/epfl-theos/aiida-flipper. The version of the code employed for this study is version 1.4. Supplementary information (SI) provides MSD plots for all materials studied using FPMD simulations, and is available. See DOI: https://doi.org/10.1039/d5ee07336g.

## References

[cit1] Armand M., Tarascon J.-M. (2008). Nature.

[cit2] Janek J., Zeier W. G. (2016). Nat. Energy.

[cit3] Yao X., Huang B., Yin J., Peng G., Huang Z., Gao C., Liu D., Xu X. (2015). Chin. Phys. B.

[cit4] Schmuch R., Wagner R., Hörpel G., Placke T., Winter M. (2018). Nat. Energy.

[cit5] Scrosati B., Garche J. (2010). J. Power Sources.

[cit6] Bates J., Dudney N., Neudecker B., Ueda A., Evans C. (2000). Solid State Ionics.

[cit7] Nishi Y. (2001). J. Power Sources.

[cit8] Liu H., Zhang G., Zheng X., Chen F., Duan H. (2020). Int. J. Extreme Manuf..

[cit9] Bachman J. C., Muy S., Grimaud A., Chang H.-H., Pour N., Lux S. F., Paschos O., Maglia F., Lupart S., Lamp P. (2016). et al.. Chem. Rev..

[cit10] Lim H.-D., Park J.-H., Shin H.-J., Jeong J., Kim J. T., Nam K.-W., Jung H.-G., Chung K. Y. (2020). Energy Storage Mater..

[cit11] Ohno S., Banik A., Dewald G. F., Kraft M. A., Krauskopf T., Minafra N., Till P., Weiss M., Zeier W. G. (2020). Prog. Energy.

[cit12] Zheng F., Kotobuki M., Song S., Lai M. O., Lu L. (2018). J. Power Sources.

[cit13] Meesala Y., Jena A., Chang H., Liu R.-S. (2017). ACS Energy Lett..

[cit14] Zheng F., Kotobuki M., Song S., Lai M. O., Lu L. (2018). J. Power Sources.

[cit15] Hagman L.-O., Kierkegaard P., Karvonen P., Virtanen A. I., Paasivirta J. (1968). Acta Chem. Scand..

[cit16] Guin M., Tietz F. (2015). J. Power Sources.

[cit17] Sudreau F., Petit D., Boilot J. (1989). J. Solid State Chem..

[cit18] Jian Z., Hu Y.-S., Ji X., Chen W. (2017). Adv. Mater..

[cit19] Aono H., Sugimoto E., Sadaoka Y., Imanaka N., Adachi G.-Y. (1990). J. Electrochem. Soc..

[cit20] Loutati A., Guillon O., Tietz F., Fattakhova-Rohlfing D. (2022). Open Ceram..

[cit21] Kaur G., Sivasubramanian S. C., Dalvi A. (2022). Electrochim. Acta.

[cit22] Alpen U. v, Rabenau A., Talat G. (1977). Appl. Phys. Lett..

[cit23] Adalati R., Sharma M., Sharma S., Kumar A., Malik G., Boukherroub R., Chandra R. (2022). J. Energy Storage.

[cit24] Li X., Liang J., Chen N., Luo J., Adair K. R., Wang C., Banis M. N., Sham T.-K., Zhang L., Zhao S. (2019). et al.. Angew. Chem..

[cit25] Nie X., Hu J., Li C. (2023). Interdiscip. Mater..

[cit26] Matsuo M., Orimo S.-i (2011). Adv. Energy Mater..

[cit27] Mohtadi R., Orimo S.-i (2016). Nat. Rev. Mater..

[cit28] Inaguma Y., Liquan C., Itoh M., Nakamura T., Uchida T., Ikuta H., Wakihara M. (1993). Solid State Commun..

[cit29] Stramare S., Thangadurai V., Weppner W. (2003). Chem. Mater..

[cit30] Deiseroth H.-J., Kong S.-T., Eckert H., Vannahme J., Reiner C., Zaiß T., Schlosser M. (2008). Angew. Chem..

[cit31] Thangadurai V., Kaack H., Weppner W. J. (2003). J. Am. Ceram. Soc..

[cit32] Murugan R., Thangadurai V., Weppner W. (2007). et al.. Angew. Chem., Int. Ed..

[cit33] Hong H.-P. (1978). Mater. Res. Bull..

[cit34] Hu Y.-W., Raistrick I., Huggins R. A. (1977). J. Electrochem. Soc..

[cit35] Khorassani A., West A. (1982). Solid State Ionics.

[cit36] Rodger A., Kuwano J., West A. (1985). Solid State Ionics.

[cit37] Deng Y., Eames C., Chotard J.-N., Lalère F., Seznec V., Emge S., Pecher O., Grey C. P., Masquelier C., Islam M. S. (2015). J. Am. Chem. Soc..

[cit38] Kanno R., Murayama M. (2001). J. Electrochem. Soc..

[cit39] Tao B., Ren C., Li H., Liu B., Jia X., Dong X., Zhang S., Chang H. (2022). Adv. Funct. Mater..

[cit40] Kamaya N., Homma K., Yamakawa Y., Hirayama M., Kanno R., Yonemura M., Kamiyama T., Kato Y., Hama S., Kawamoto K. (2011). et al.. Nat. Mater..

[cit41] Liang F., Sun Y., Yuan Y., Huang J., Hou M., Lu J. (2021). Mater. Today.

[cit42] Koinuma H., Takeuchi I. (2004). Nat. Mater..

[cit43] Xiang X.-D., Sun X., Briceno G., Lou Y., Wang K.-A., Chang H., Wallace-Freedman W. G., Chen S.-W., Schultz P. G. (1995). Science.

[cit44] Takeuchi I., Famodu O., Read J., Aronova M., Chang K.-S., Craciunescu C., Lofland S., Wuttig M., Wellstood F., Knauss L. (2003). et al.. Nat. Mater..

[cit45] Castelli I. E., Olsen T., Datta S., Landis D. D., Dahl S., Thygesen K. S., Jacobsen K. W. (2012). Energy Environ. Sci..

[cit46] Pizzi G., Cepellotti A., Sabatini R., Marzari N., Kozinsky B. (2016). Comput. Mater. Sci..

[cit47] Mounet N., Gibertini M., Schwaller P., Campi D., Merkys A., Marrazzo A., Sohier T., Castelli I. E., Cepellotti A., Pizzi G. (2018). et al.. Nat. Nanotechnol..

[cit48] Tufail M. K., Zhai P., Jia M., Zhao N., Guo X. (2023). Energy Mater. Adv..

[cit49] Xiao Y., Jun K., Wang Y., Miara L. J., Tu Q., Ceder G. (2021). Adv. Energy Mater..

[cit50] Muy S., Le Mercier T., Dufour M., Braida M.-D., Emery A. A., Marzari N. (2025). Chem. Mater..

[cit51] Hohenberg P., Kohn W. (1964). Phys. Rev..

[cit52] Kohn W., Sham L. J. (1965). Phys. Rev..

[cit53] Shishkin M., Marsman M., Kresse G. (2007). Phys. Rev. Lett..

[cit54] De Gennaro R., Colonna N., Linscott E., Marzari N. (2022). Phys. Rev. B.

[cit55] Miceli G., Chen W., Reshetnyak I., Pasquarello A. (2018). Phys. Rev. B.

[cit56] Himmetoglu B., Floris A., De Gironcoli S., Cococcioni M. (2014). Int. J. Quantum Chem..

[cit57] Marzari N., Ferretti A., Wolverton C. (2021). Nat. Mater..

[cit58] Cohen A. J., Mori-Sánchez P., Yang W. (2012). Chem. Rev..

[cit59] Muy S., Voss J., Schlem R., Koerver R., Sedlmaier S. J., Maglia F., Lamp P., Zeier W. G., Shao-Horn Y. (2019). iScience.

[cit60] Sendek A. D., Yang Q., Cubuk E. D., Duerloo K.-A. N., Cui Y., Reed E. J. (2017). Energy Environ. Sci..

[cit61] Perdew J. P., Burke K., Ernzerhof M. (1996). Phys. Rev. Lett..

[cit62] Richards W. D., Miara L. J., Wang Y., Kim J. C., Ceder G. (2016). Chem. Mater..

[cit63] Borodin O., Olguin M., Spear C. E., Leiter K. W., Knap J. (2015). Nanotechnology.

[cit64] Sun Y., Yan W., An L., Wu B., Zhong K., Yang R. (2017). Solid State Ionics.

[cit65] Jian S., Li H., Jia X., Zhong D., Tao B., He X., Wang G., Chang H. (2024). FlatChem.

[cit66] Park K. H., Bai Q., Kim D. H., Oh D. Y., Zhu Y., Mo Y., Jung Y. S. (2018). Adv. Energy Mater..

[cit67] Pokluda J., Černy M., Šob M., Umeno Y. (2015). Prog. Mater. Sci..

[cit68] Materzanini G., Chiarotti T., Marzari N. (2023). npj Comput. Mater..

[cit69] Ren Y., Shen Y., Lin Y., Nan C.-W. (2015). Electrochem. Commun..

[cit70] Porz L., Swamy T., Sheldon B. W., Rettenwander D., Frömling T., Thaman H. L., Berendts S., Uecker R., Carter W. C., Chiang Y.-M. (2017). Adv. Energy Mater..

[cit71] Kerman K., Luntz A., Viswanathan V., Chiang Y.-M., Chen Z. (2017). J. Electrochem. Soc..

[cit72] Gao Z., Sun H., Fu L., Ye F., Zhang Y., Luo W., Huang Y. (2018). Adv. Mater..

[cit73] Hull S. (2004). Rep. Prog. Phys..

[cit74] Knauth P., Tuller H. L. (2002). J. Am. Ceram. Soc..

[cit75] Alder B. J., Wainwright T. E. (1959). J. Chem. Phys..

[cit76] Rahman A. (1964). Phys. Rev..

[cit77] Frenkel D. and Smit B., Understanding molecular simulation: from algorithms to applications, Elsevier, 2023

[cit78] Tuckerman M. , Statistical Mechanics: Theory and Molecular Simulation, OUP, Oxford, 2010

[cit79] Allen M. P. and Tildesley D. J., Computer simulation of liquids, Oxford University Press, 2017

[cit80] Adams S., Rao R. P. (2012). J. Mater. Chem..

[cit81] Born M. and Heisenberg W., Original Scientific Papers Wissenschaftliche Originalarbeiten, 1985, pp. 216–246

[cit82] Tuckerman M. E. (2002). J. Phys.: Condens. Matter.

[cit83] Car R., Parrinello M. (1985). Phys. Rev. Lett..

[cit84] Materzanini G., Kahle L., Marcolongo A., Marzari N. (2021). Phys. Rev. Mater..

[cit85] Du Y. A., Holzwarth N. (2007). J. Electrochem. Soc..

[cit86] Lepley N., Holzwarth N., Du Y. A. (2013). Phys. Rev. B: Condens. Matter Mater. Phys..

[cit87] Marcolongo A., Marzari N. (2017). Phys. Rev. Mater..

[cit88] He X., Zhu Y., Mo Y. (2017). Nat. Commun..

[cit89] Morgan B. J. (2017). R. Soc. Open Sci..

[cit90] Brown I. (1992). Acta Crystallogr., Sect. B: Struct. Sci..

[cit91] Anurova N., Blatov V., Ilyushin G., Blatova O., Ivanov-Schitz A., Dem'Yanets L. (2008). Solid State Ionics.

[cit92] Avdeev M., Sale M., Adams S., Rao R. P. (2012). Solid State Ionics.

[cit93] Xiao R., Li H., Chen L. (2015). J. Materiomics.

[cit94] Swenson J. (2000). et al.. Phys. Rev. Lett..

[cit95] Müller C., Zienicke E., Adams S., Habasaki J., Maass P. (2007). Phys. Rev. B: Condens. Matter Mater. Phys..

[cit96] Aniya M., Wakamura K. (1996). Phys. B.

[cit97] Wakamura K. (1997). Phys. Rev. B: Condens. Matter Mater. Phys..

[cit98] Batatia I., Kovacs D. P., Simm G., Ortner C., Csányi G. (2022). Adv. Neural Inf. Process. Syst..

[cit99] Batatia I., Benner P., Chiang Y., Elena A. M., Kovács D. P., Riebesell J., Advincula X. R., Asta M., Avaylon M., Baldwin W. J., Berger F., Bernstein N., Bhowmik A., Bigi F., Blau S. M., Carare V., Ceriotti M., Chong S., Darby J. P., De S., Pia F. D., Deringer V. L., ElijoÅ¡ius R., El-Machachi Z., Fako E., Falcioni F., Ferrari A. C., Gardner J. L. A., Gawkowski M. J., Genreith-Schriever A., George J., Goodall R. E. A., Grandel J., Grey C. P., Grigorev P., Han S., Handley W., Heenen H. H., Hermansson K., Ho C. H., Hofmann S., Holm C., Jaafar J., Jakob K. S., Jung H., Kapil V., Kaplan A. D., Karimitari N., Kermode J. R., Kourtis P., Kroupa N., Kullgren J., Kuner M. C., Kuryla D., Liepuoniute G., Lin C., Margraf J. T., Magdau I.-B., Michaelides A., Moore J. H., Naik A. A., Niblett S. P., Norwood S. W., O’Neill N., Ortner C., Persson K. A., Reuter K., Rosen A. S., Rosset L. A. M., Schaaf L. L., Schran C., Shi B. X., Sivonxay E., Stenczel T. K., Sutton C., Svahn V., Swinburne T. D., Tilly J., van der Oord C., Vargas S., Varga-Umbrich E., Vegge T., Vondrák M., Wang Y., Witt W. C., Wolf T., Zills F., Csányi G. (2025). J. Chem. Phys..

[cit100] Chen C., Ong S. P. (2022). Nat. Comput. Sci..

[cit101] Deng B., Zhong P., Jun K., Riebesell J., Han K., Bartel C. J., Ceder G. (2023). Nat. Mach. Intell..

[cit102] Merchant A., Batzner S., Schoenholz S. S., Aykol M., Cheon G., Cubuk E. D. (2023). Nature.

[cit103] Riebesell J. , Goodall R. E., Jain A., Benner P., Persson K. A. and Lee A. A., *arXiv*, 2023, preprint, arXiv:2308.14920 10.48550/arXiv.2308.14920, https://arxiv.org/abs/2308.14920

[cit104] Yu H. , Giantomassi M., Materzanini G. and Rignanese G.-M., *arXiv*, 2024, preprint, arXiv:2403.05729 10.48550/arXiv.2403.05729, https://arxiv.org/abs/2403.05729

[cit105] Focassio B. , Freitas L. P. M. and Schleder G. R., *arXiv*, 2024, preprint, arXiv:2403.04217 10.48550/arXiv.2403.04217, https://arxiv.org/abs/2403.04217

[cit106] Wines D. and Choudhary K., *arXiv*, 2024, preprint, arXiv:2412.10516 10.48550/arXiv.2412.10516, https://arxiv.org/abs/2412.10516

[cit107] Li Z. , Jun K., Deng B. and Ceder G., *arXiv*, 2025, preprint, arXiv:2511.20964 10.48550/arXiv.2511.20964, https://arxiv.org/abs/2511.20964

[cit108] Lian J. , Fu X., Gong X., Xiao R. and Li H., *arXiv*, 2025, preprint, arXiv:2507.02334 10.48550/arXiv.2507.02334, https://arxiv.org/abs/2507.02334

[cit109] Agrawal A., Choudhary A. (2016). APL Mater..

[cit110] Laskowski F. A. L., McHaffie D. B., See K. A. (2023). Energy Environ. Sci..

[cit111] Sendek A. D., Yang Q., Cubuk E. D., Duerloo K.-A. N., Cui Y., Reed E. J. (2017). Energy Environ. Sci..

[cit112] Zhang Y., He X., Chen Z., Bai Q., Nolan A. M., Roberts C. A., Banerjee D., Matsunaga T., Mo Y., Ling C. (2019). Nat. Commun..

[cit113] Hargreaves C. J., Gaultois M. W., Daniels L. M., Watts E. J., Kurlin V. A., Moran M., Dang Y., Morris R., Morscher A., Thompson K. (2023). npj Comput. Mater..

[cit114] Chen C. , Nguyen D. T., Lee S. J., Baker N. A., Karakoti A. S., Lauw L., Owen C., Mueller K. T., Bilodeau B. A. and Murugesan V. et al., *arXiv*, 2024, preprint, arXiv:2401.04070 10.48550/arXiv.2401.04070, https://arxiv.org/abs/2401.04070 38980280

[cit115] Zunger A. (2018). Nat. Rev. Chem..

[cit116] Cheetham A. K., Seshadri R. (2024). Chem. Mater..

[cit117] Kahle L., Marcolongo A., Marzari N. (2020). Energy Environ. Sci..

[cit118] Jun K., Sun Y., Xiao Y., Zeng Y., Kim R., Kim H., Miara L. J., Im D., Wang Y., Ceder G. (2022). Nat. Mater..

[cit119] Kahle L., Marcolongo A., Marzari N. (2018). Phys. Rev. Mater..

[cit120] Curtarolo S., Hart G. L., Nardelli M. B., Mingo N., Sanvito S., Levy O. (2013). Nat. Mater..

[cit121] Alberi K., Nardelli M. B., Zakutayev A., Mitas L., Curtarolo S., Jain A., Fornari M., Marzari N., Takeuchi I., Green M. L. (2018). et al.. J. Phys. D: Appl. Phys..

[cit122] Buneman P. , Khanna S. and Wang-Chiew T., Database Theory—ICDT 2001: 8th International Conference London, UK, January 4-6, 2001 Proceedings 8, 2001, pp. 316-330

[cit123] Moreau L. and Groth P., Provenance: an introduction to PROV, Springer Nature, 2022

[cit124] Huber S. P., Zoupanos S., Uhrin M., Talirz L., Kahle L., Häuselmann R., Gresch D., Müller T., Yakutovich A. V., Andersen C. W. (2020). et al.. Sci. Data.

[cit125] Uhrin M., Huber S. P., Yu J., Marzari N., Pizzi G. (2021). Comput. Mater. Sci..

[cit126] Wilkinson M. D., Dumontier M., Aalbersberg I. J., Appleton G., Axton M., Baak A., Blomberg N., Boiten J.-W., da Silva Santos L. B., Bourne P. E. (2016). et al.. Sci. Data.

[cit127] Gražulis S., Daškevič A., Merkys A., Chateigner D., Lutterotti L., Quirós M., Serebryanaya N. R., Moeck P., Downs R. T., Le Bail A. (2011). Nucleic Acids Res..

[cit128] Belsky A., Hellenbrandt M., Karen V. L., Luksch P. (2002). Acta Crystallogr., Sect. B: Struct. Sci..

[cit129] Blokhin E. and Villars P., The PAULING FILE Project and Materials Platform for Data Science: From Big Data Toward Materials Genome, Springer International Publishing, Cham, 2018, pp. 1–26

[cit130] Vaitkus A., Merkys A., Gražulis S. (2021). J. Appl. Crystallogr..

[cit131] Merkys A., Vaitkus A., Butkus J., Okulič-Kazarinas M., Kairys V., Gražulis S. (2016). J. Appl. Crystallogr..

[cit132] Gražulis S., Merkys A., Vaitkus A., Okulič-Kazarinas M. (2015). J. Appl. Crystallogr..

[cit133] Hart G. L., Forcade R. W. (2008). Phys. Rev. B: Condens. Matter Mater. Phys..

[cit134] Grau-Crespo R., Hamad S., Catlow C. R. A., De Leeuw N. (2007). J. Phys.: Condens. Matter.

[cit135] Mustapha S., D’Arco P., De La Pierre M., Noël Y., Ferrabone M., Dovesi R. (2013). J. Phys.: Condens. Matter.

[cit136] Hundt R., Schön J., Jansen M. (2006). J. Appl. Crystallogr..

[cit137] Ong S. P., Richards W. D., Jain A., Hautier G., Kocher M., Cholia S., Gunter D., Chevrier V. L., Persson K. A., Ceder G. (2013). Comput. Mater. Sci..

[cit138] Giannozzi P., Baroni S., Bonini N., Calandra M., Car R., Cavazzoni C., Ceresoli D., Chiarotti G. L., Cococcioni M., Dabo I., Dal Corso A., de Gironcoli S., Fabris S., Fratesi G., Gebauer R., Gerstmann U., Gougoussis C., Kokalj A., Lazzeri M., Martin-Samos L., Marzari N., Mauri F., Mazzarello R., Paolini S., Pasquarello A., Paulatto L., Sbraccia C., Scandolo S., Sclauzero G., Seitsonen A. P., Smogunov A., Umari P., Wentzcovitch R. M. (2009). J. Phys.: Condens. Matter.

[cit139] Giannozzi P., Andreussi O., Brumme T., Bunau O., Nardelli M. B., Calandra M., Car R., Cavazzoni C., Ceresoli D., Cococcioni M., Colonna N., Carnimeo I., Corso A. D., de Gironcoli S., Delugas P., Jr R. A. D., Ferretti A., Floris A., Fratesi G., Fugallo G., Gebauer R., Gerstmann U., Giustino F., Gorni T., Jia J., Kawamura M., Ko H.-Y., Kokalj A., Küçükbenli E., Lazzeri M., Marsili M., Marzari N., Mauri F., Nguyen N. L., Nguyen H.-V., de-la Roza A. O., Paulatto L., Poncé S., Rocca D., Sabatini R., Santra B., Schlipf M., Seitsonen A. P., Smogunov A., Timrov I., Thonhauser T., Umari P., Vast N., Wu X., Baroni S. (2017). J. Phys.: Condens. Matter.

[cit140] Perdew J. P., Ruzsinszky A., Csonka G. I., Vydrov O. A., Scuseria G. E., Constantin L. A., Zhou X., Burke K. (2008). Phys. Rev. Lett..

[cit141] Prandini G., Marrazzo A., Castelli I. E., Mounet N., Marzari N. (2018). npj Comput. Mater..

[cit142] Willand A., Kvashnin Y. O., Genovese L., Vázquez-Mayagoitia Á., Deb A. K., Sadeghi A., Deutsch T., Goedecker S. (2013). J. Chem. Phys..

[cit143] Dal Corso A. (2014). Comput. Mater. Sci..

[cit144] Garrity K. F., Bennett J. W., Rabe K. M., Vanderbilt D. (2014). Comput. Mater. Sci..

[cit145] Topsakal M., Wentzcovitch R. (2014). Comput. Mater. Sci..

[cit146] Schlipf M., Gygi F. (2015). Comput. Phys. Commun..

[cit147] Van Setten M. J., Giantomassi M., Bousquet E., Verstraete M. J., Hamann D. R., Gonze X., Rignanese G.-M. (2018). Comput. Phys. Commun..

[cit148] Marzari N., Vanderbilt D., De Vita A., Payne M. (1999). Phys. Rev. Lett..

[cit149] Thakur T. , AiiDA-flipper, 2024, https://github.com/epfl-theos/aiida-flipper

[cit150] Kahle L. , The supercell creator, 2019, https://github.com/lekah/supercellor

[cit151] Bussi G., Donadio D., Parrinello M. (2007). J. Chem. Phys..

[cit152] Haven Y. (1965). Phys. Chem. Glasses.

[cit153] Einstein A. (1905). Ann. Phys..

[cit154] Kahle L. , Suite for Analysis of Molecular Simulations (SAMOS), 2024, https://github.com/lekah/samos

[cit155] He X., Zhu Y., Epstein A., Mo Y. (2018). npj Comput. Mater..

[cit156] Sivia D. and Skilling J., Data analysis: a Bayesian tutorial, OUP, Oxford, 2006

[cit157] Wang Y., Richards W. D., Ong S. P., Miara L. J., Kim J. C., Mo Y., Ceder G. (2015). Nat. Mater..

[cit158] Dathar G. K. P., Balachandran J., Kent P. R., Rondinone A. J., Ganesh P. (2017). J. Mater. Chem. A.

[cit159] Zhou L., Minafra N., Zeier W. G., Nazar L. F. (2021). Acc. Chem. Res..

[cit160] Yang H., Wu N. (2022). Energy Sci. Eng..

[cit161] Wang D., Shi H., Wang S., Wu X., Jiang W., Liang S., Xu Z. (2024). Coord. Chem. Rev..

[cit162] Kozhevnikov A. and Pintarelli S., SIRIUS, 2023, https://github.com/electronic-structure/SIRIUS

[cit163] De La Pierre M., Orlando R., Maschio L., Doll K., Ugliengo P., Dovesi R. (2011). J. Comput. Chem..

[cit164] Zhu Z., Chu I.-H., Ong S. P. (2017). Chem. Mater..

[cit165] Kataoka K., Awaka J., Kijima N., Hayakawa H., Ohshima K., Akimoto J. (2011). Chem. Mater..

[cit166] Fernández-Gamboa J., Tielens F., Zulueta Y. (2022). Mater. Sci. Semicond. Process..

[cit167] Kuhn A. (2018). J. Mater. Chem. A.

[cit168] Chu I.-H., Nguyen H., Hy S., Lin Y.-C., Wang Z., Xu Z., Deng Z., Meng Y. S., Ong S. P. (2016). ACS Appl. Mater. Interfaces.

[cit169] Yamane H., Shibata M., Shimane Y., Junke T., Seino Y., Adams S., Minami K., Hayashi A., Tatsumisago M. (2007). Solid State Ionics.

[cit170] Xiao Y., Jun K., Wang Y., Miara L., Tu Q., Ceder G. (2021). Adv. Energy Mater..

[cit171] Kirklin S., Chan M., Trahey L., Thackeray M., Wolverton C. (2014). Phys. Chem. Chem. Phys..

[cit172] Howard M., Clemens O., Slater P., Anderson P. (2015). J. Alloys Compd..

[cit173] Boukamp B., Huggins R. (1976). Phys. Lett. A.

[cit174] Boukamp B., Huggins R. (1978). Mater. Res. Bull..

[cit175] Shannon R. , Taylor B., English A. and Berzins T., International Symposium on Solid Ionic and Ionic-Electronic Conductors, 1977, pp. 783–796

[cit176] Adelstein N., Wood B. (2016). Chem. Mater..

[cit177] Wu F., Lee J., Xiao Y., Yushin G. (2016). Nano Energy.

[cit178] Yang W. (2022). ACS Appl. Mater. Interfaces.

[cit179] Yamane H., Kikkawa S., Koizumi M. (1987). J. Solid State Chem..

[cit180] Laskowski F., McHaffie D., See K. (2023). Energy Environ. Sci..

[cit181] Bianchini F., Fjellvåg H., Vajeeston P. (2018). Mater. Lett..

[cit182] Kaib T., Bron P., Haddadpour S., Mayrhofer L., Pastewka L., Järvi T. T., Moseler M., Roling B., Dehnen S. (2013). Chem. Mater..

[cit183] Ulvestad A., Mæhlen J., Kirkengen M. (2018). J. Power Sources.

[cit184] Snydacker D., Hegde V., Wolverton C. (2017). J. Electrochem. Soc..

[cit185] Toffoletti L. (2016). Chem. – Eur. J..

[cit186] Yeandel S., Scanlon D., Goddard P. (2019). J. Mater. Chem. A.

[cit187] Hang B., Ohnishi T., Osada M., Xu X., Takada K., Sasaki T. (2010). J. Power Sources.

[cit188] Huang W. (2022). J. Am. Chem. Soc..

[cit189] Xu K. (2004). Chem. Rev..

[cit190] Emly A., Kioupakis E., Ven A. (2013). Chem. Mater..

[cit191] Zhao Y., Daemen L. (2012). J. Am. Chem. Soc..

[cit192] Tachez M., Malugani J.-P., Mercier R., Robert G. (1984). Solid State Ionics.

[cit193] Liu Z. (2013). J. Am. Chem. Soc..

[cit194] Al-Qawasmeh A., Obeidat A., Shukri A., Holzwarth N. (2020). J. Electrochem. Soc..

[cit195] Obeidat A., Al-Qawasmeh A., Shukri A. (2022). J. Electrochem. Soc..

[cit196] Lim H., Kim S.-C., Kim J., Kim Y.-I., Kim S.-J. (2018). J. Solid State Chem..

[cit197] Huang W. (2019). J. Solid State Chem..

[cit198] Weppner W., Hartwig P., Rabenau A. (1981). J. Power Sources.

[cit199] Landgraf V., Famprikis T., Leeuw J., Bannenberg L., Ganapathy S., Wagemaker M. (2023). ACS Appl. Energy Mater..

[cit200] Nomura E., Greenblatt M. (1984). J. Solid State Chem..

[cit201] Aono H., Sugimoto E., Sadaoka Y., Imanaka N., Adachi G.-Y. (1993). Solid State Ionics.

[cit202] Martínez-Juárez A., Iglesias J., Rojo J. (1996). Solid State Ionics.

[cit203] Chang C.-M., Hong S.-H., Park H.-M. (2005). Solid State Ionics.

[cit204] Tell B., Wagner S., Kasper H. (1977). J. Electrochem. Soc..

[cit205] Rao F.-Y. (2016). Chin. Phys. B.

[cit206] Rosenman A., Markevich E., Salitra G., Aurbach D., Garsuch A., Chesneau F. (2015). Adv. Energy Mater..

[cit207] Su D., Zhou D., Wang C., Wang G. (2018). Adv. Funct. Mater..

[cit208] Eichinger G. (1981). Solid State Ionics.

[cit209] Richards W. D., Wang Y., Miara L. J., Kim J. C., Ceder G. (2016). Energy Environ. Sci..

[cit210] Kaup K., Lalère F., Huq A., Shyamsunder A., Adermann T., Hartmann P., Nazar L. F. (2018). Chem. Mater..

[cit211] Jiang X., Xu H., Mao H., Yang J., Qian Y. (2016). J. Power Sources.

[cit212] Wang Y., Xiao X., Li Q., Pang H. (2018). Small.

[cit213] Huang Y., Jiang Y., Zhou Y., Hu Z., Zhu X. (2019). ChemElectroChem.

[cit214] Walther F., Strauss F., Wu X., Mogwitz B., Hertle J., Sann J., Rohnke M., Brezesinski T., Janek J. (2021). Chem. Mater..

[cit215] Jiang H., Wang S., Zhang B., Shao Y., Wu Y., Zhao H., Lei Y., Hao X. (2020). Chem. Eng. J..

[cit216] Spiesser M., Palvadeau P., Guillot C., Cerisier J. (1983). Solid State Ionics.

[cit217] Chadwick A. V. , Defect and Diffusion Forum, 1993, pp. 1015–1040

[cit218] Verma R., Park C.-J., Kothandaraman R., Varadaraju U. (2017). Electrochim. Acta.

[cit219] Burba C. M., Frech R. (2005). J. Electrochem. Soc..

[cit220] Cui W.-J., Yi J., Chen L., Wang C.-X., Xia Y.-Y. (2012). J. Power Sources.

[cit221] Tian J., Wang D., Shan Z. (2017). et al.. J. Power Sources.

[cit222] Kaib T., Haddadpour S., Kapitein M., Bron P., Schröder C., Eckert H., Roling B., Dehnen S. (2012). Chem. Mater..

[cit223] Marx R., Lissner F., Schleid T. (2006). Z. Anorg. Allg. Chem..

[cit224] Miara L. J., Suzuki N., Richards W. D., Wang Y., Kim J. C., Ceder G. (2015). J. Mater. Chem. A.

[cit225] Murayama M., Kanno R., Kawamoto Y., Kamiyama T. (2002). Solid State Ionics.

[cit226] Pompetzki M., van Wüllen L., Jansen M. (2004). Z. Anorg. Allg. Chem..

[cit227] van Wüllen L., Hildebrandt L., Jansen M. (2005). Solid State Ionics.

[cit228] Sang J., Yu Y., Wang Z., Shao G. (2020). Phys. Chem. Chem. Phys..

[cit229] Kravchenko V., Sigaryov S. (1994). J. Mater. Sci..

[cit230] Pilz T., Jansen M. (2011). Z. Anorg. Allg. Chem..

[cit231] Fanah S. J., Ramezanipour F. (2019). Solid State Sci..

[cit232] Muehle C., Dinnebier R. E., van Wüllen L., Schwering G., Jansen M. (2004). Inorg. Chem..

[cit233] Pentin I., Schön J., Jansen M. (2008). Solid State Sci..

[cit234] Ahmad A., Arof A. (2002). Ionics.

[cit235] Dissanayake M., Careem M., Bandaranayake P., Wijayasekera C. (1991). Solid State Ionics.

[cit236] Ryu J.-G., Balasubramaniam R., Aravindan V., Park S., Cho S. J., Lee Y.-S. (2023). ACS Appl. Mater. Interfaces.

[cit237] Cachau-Herreillat D., Norbert A., Maurin M., Philippot E. (1981). J. Solid State Chem..

[cit238] Pentin I. V., Saltykov V., Nuss J., Schön J. C., Jansen M. (2012). Chem. – Eur. J..

[cit239] Sabrowsky H. (1989). Z. Naturforsch..

[cit240] Ohtani T., Honjo H., Wada H. (1987). Mater. Res. Bull..

[cit241] Greuling B. (1987). Cryst. Res. Technol..

[cit242] Hellstrom E., Huggins R. (1979). Mater. Res. Bull..

[cit243] Tromme M. (1971). CR Seances Acad. Sci., Ser. C.

[cit244] Feng J.-H., Hu C.-L., Xia H.-P., Kong F., Mao J.-G. (2017). Inorg. Chem..

[cit245] Eickhoff H., Toffoletti L., Klein W., Raudaschl-Sieber G., Fässler T. F. (2017). Inorg. Chem..

[cit246] Carlson V. A., Stacy A. M. (1992). J. Solid State Chem..

[cit247] Graudejus O., Wilkinson A. P., Chacón L. C., Bartlett N. (2000). Inorg. Chem..

[cit248] Chassaing J. , PhD thesis, Gauthier-Villars, 1968

[cit249] Whittingham M. S., Gamble Jr F. R. (1975). Mater. Res. Bull..

[cit250] Zhang J.-H., Clark D. J., Weiland A., Stoyko S. S., Kim Y. S., Jang J. I., Aitken J. A. (2017). Inorg. Chem. Front..

[cit251] Yin W., Feng K., Mei D., Yao J., Fu P., Wu Y. (2012). Dalton Trans..

[cit252] De Pape R., Portier J., Grannec J., Gauthier G., Hagenmuller P. (1969). CR Acad. Sc. Paris, Sér. C.

[cit253] Hoppe R., Röhrborn H.-J. (1964). Z. Anorg. Allg. Chem..

[cit254] Toda K., Takahashi M., Teranishi T., Ye Z.-G., Sato M., Hinatsu Y. (1999). J. Mater. Chem..

[cit255] Wolfenstine J., Rangasamy E., Allen J. L., Sakamoto J. (2012). J. Power Sources.

[cit256] Novoselova A. and Simanov Y. P., Thermal and X-ray analysis of the system LiF-BeF2, MAIK Nauka/Interperiodica Publishers, 1952

[cit257] Roy D., Roy R., Osborn E. (1954). J. Am. Ceram. Soc..

[cit258] Dion M., Piffard Y., Tournoux M. (1978). J. Inorg. Nucl. Chem..

[cit259] Hyett G., Rutt O. J., Gál Z. A., Denis S. G., Hayward M. A., Clarke S. J. (2004). J. Am. Chem. Soc..

[cit260] Marx R. (1995). Z. Naturforsch. B.

[cit261] Bräunling D. , Pecher O., Trots D. M., Senyshyn A., Zherebtsov D. A., Haarmann F. and Niewa R., Synthesis, crystal structure and lithium motion of Li_*8*_SeN_*2*_ and Li_*8*_TeN_*2*_, 2010

[cit262] Bolte M., Lerner H.-W. (2001). Acta Crystallogr., Sect. E: Crystallogr. Commun..

[cit263] Pfitzner A., Cockcroft J., Solinas I., Litz H. (1993). Z. Anorg. Allg. Chem..

[cit264] Yadav M. K., Sanyal B. (2015). J. Alloys Compd..

[cit265] El Maslout A., Motte J.-P., Courtois A., Gleitzer C. (1975). J. Solid State Chem..

[cit266] Brandes R., Hoppe R. (1994). Z. Anorg. Allg. Chem..

[cit267] Zhang J.-H., Clark D. J., Brant J. A., Sinagra C. W., Kim Y. S., Jang J. I., Aitken J. A. (2015). Dalton Trans..

[cit268] Mahendran K., Sujatha K., Sridharan R., Gnanasekaran T. (2003). J. Alloys Compd..

[cit269] Isaenko L., Yelisseyev A., Lobanov S., Titov A., Petrov V., Zondy J.-J., Krinitsin P., Merkulov A., Vedenyapin V., Smirnova J. (2003). Cryst. Res. Technol..

[cit270] Schnering H. v, Wichelhaus W. (1972). Naturwissenschaften.

[cit271] Liu H., Wu H., Yu H., Hu Z., Wu Y. (2019). Dalton Trans..

[cit272] Le Page Y., Strobel P. (1980). Acta Crystallogr., Sect. B: Struct. Sci..

[cit273] Peng N., Cheng X., Yu H., Zhu H., Liu T., Zheng R., Shui M., Xie Y., Shu J. (2019). Energy Storage Mater..

[cit274] Huang F. Q., Yang Y., Flaschenriem C., Ibers J. A. (2001). Inorg. Chem..

[cit275] Laligant Y. (1992). Eur. J. Solid State Inorg. Chem..

[cit276] Hasegawa T., Yamane H. (2014). Dalton Trans..

[cit277] Lindemann A., Kuchinke J., Köster C., Hammerschmidt A., Döch M., Pruss T., Krebs B. (2001). Phosphorus, Sulfur Silicon Relat. Elem..

[cit278] Klevtsova R., Solodovnikov S., Glinskaya L., Alekseev V., Khalbaeva K., Khaikina E. (1997). J. Struct. Chem..

[cit279] Caurant D. , Wallez G., Majérus O., Roisine G. and Charpentier T., Lead in Glassy Materials in Cultural Heritage, 2024, pp. 37–92

[cit280] Kacem I. B., Gautron L., Coillot D., Neuville D. R. (2017). Chem. Geol..

[cit281] Tomashyk V. , Quaternary alloys based on II-VI semiconductors, CRC Press, 2014

[cit282] Li M., Verena-Mudring A. (2016). Cryst. Growth Des..

[cit283] Kelleher B., Dolan K., Anderson M., Sridharan K. (2016). Nucl. Technol..

[cit284] Xie J., Xie Y. (2016). Chem. – Eur. J..

[cit285] Jalem R., Yamamoto Y., Shiiba H., Nakayama M., Munakata H., Kasuga T., Kanamura K. (2013). Chem. Mater..

[cit286] Čančarević Ž. P., Schön J. C., Jansen M. (2007). Chem. – Eur. J..

[cit287] Dvoryanova E., Kondratyuk I., Garkushin I. (2010). Russ. J. Inorg. Chem..

[cit288] Manyakova A., Egorova E., Garkushin I. (2018). Russ. J. Inorg. Chem..

[cit289] Hauck J. (1969). Z. Naturforsch. B.

[cit290] Meyer G., Gaebell H.-C. (1983). Mater. Res. Bull..

[cit291] Nagarkar V., Ovechkina E., Bhandari H., Soundara-Pandian L., More M., Riedel R., Miller S. (2015). Phys. Proc..

[cit292] Ortner T. S., Scheifers J. P., Flores J., Zhang Y., Iyer A. K., Gesing T. M., Fokwa B. P. (2019). Eur. J. Inorg. Chem..

[cit293] Hibble S., Fawcett I., Hannon A. (1997). Acta Crystallogr., Sect. B: Struct. Sci..

[cit294] Schön J., Wevers M., Jansen M. (2000). Solid State Sci..

[cit295] Sorescu M., Knobbe E., Martin J., Barrie J., Barb D. (1995). J. Mater. Sci..

[cit296] He X., Bai Q., Liu Y., Nolan A. M., Ling C., Mo Y. (2019). Adv. Energy Mater..

[cit297] Feng S., Wang Z., Zhang G., Yue P., Pan W., Lu Q., Guo H., Li X., Yan G., Wang J. (2024). Phys. Chem. Chem. Phys..

[cit298] Zintl E., Brauer G. (1935). Z. Elektrochem. Angew. Phys. Chem..

[cit299] Li W., Li M., Ren H., Kim J. T., Li R., Sham T.-K., Sun X. (2025). Energy Environ. Sci..

[cit300] Lapp T., Skaarup S., Hooper A. (1983). Solid State Ionics.

[cit301] Tapia-Ruiz N., Segalés M., Gregory D. H. (2013). Coord. Chem. Rev..

[cit302] Xie S. R., Honrao S. J., Lawson J. W. (2024). Chem. Mater..

[cit303] Thakur T. S., Ercole L., Marzari N. (2026). Energy Environ. Sci..

