# Supplementary material for: Novel fast Li-ion conductors for solid-state electrolytes from first-principles
Source: Energy Environ Sci. 2026 Apr 22;19(10):3214–35. doi: 10.1039/d5ee07336g (PMC13147500; doi:10.1039/d5ee07336g)
Supplement: EE-019-D5EE07336G-s001 [file EE-019-D5EE07336G-s001.pdf]

Supplementary Information (SI) for Energy & Environmental Science.
This journal is © The Royal Society of Chemistry 2026

Supplementary information†

Novel fast Li-ion conductors for solid-state electrolytes from first-principles

Tushar Singh Thakur,[*a] Loris Ercole,[a] and Nicola Marzari[a,b,c]

The following sections exhaustively illustrate the MSD plots derived from FPMD simulations of all the structures discussed in the main text.

S1 Fast Li-ion conductors

We discover 9 novel fast Li-ion conductors that exhibit significant diffusion at low temperatures along with desirable activation energy. We show the MSD plots at 1000 K, 750 K, 600 K and 500 K.

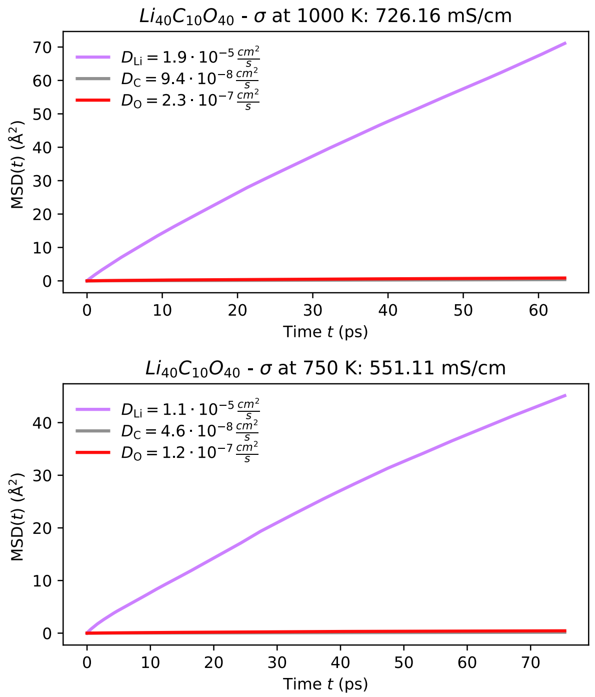

Fig. S1 MSD(t) plot of Li along with host-lattice species of Li_4CO_4 at all temperatures studied with FPMD.

We show the iso-surface plots at 600 K for the oxides and nitride.

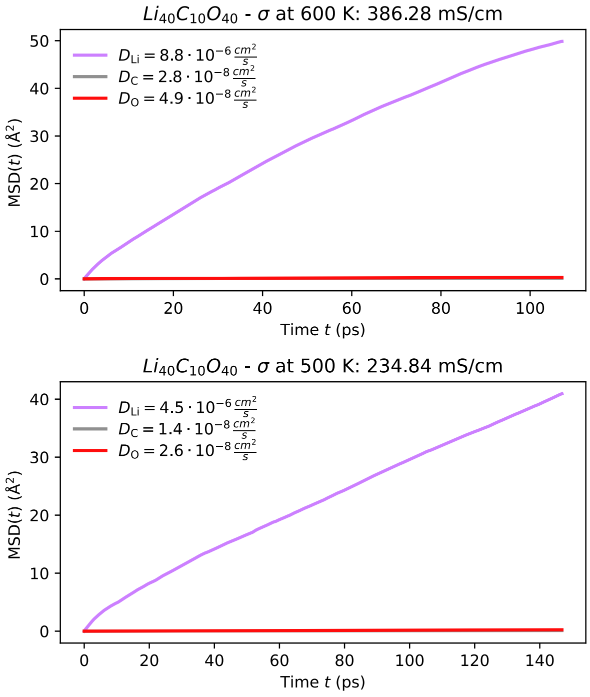

Fig. S2 MSD(t) plot of Li along with host-lattice species of Li_4CO_4 at all temperatures studied with FPMD.

* E-mail: tushar.thakur@epfl.ch
[a] Theory and Simulation of Materials (THEOS), and National Centre for Computational Design and Discovery of Novel Materials (MARVEL), École Polytechnique Fédérale de Lausanne, CH-1015 Lausanne, Switzerland
[b] PSI Center for Scientific Computing, Theory and Data, Paul Scherrer Institute, 5232 Villigen PSI, Switzerland
[c] Theory of Condensed Matter, Cavendish Laboratory, University of Cambridge, Cambridge CB3 0US, United Kingdom

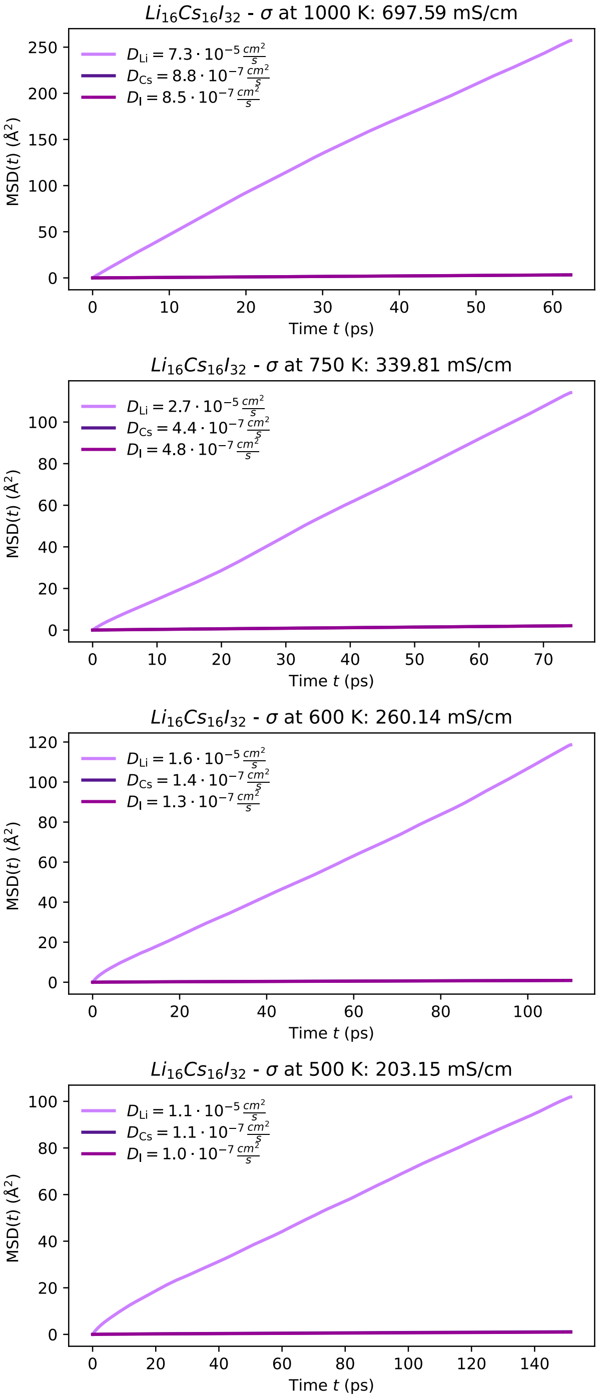

Fig. S3 MSD(t) plot of Li along with host-lattice species of $LiCsI_2$ at all temperatures studied with FPMD.

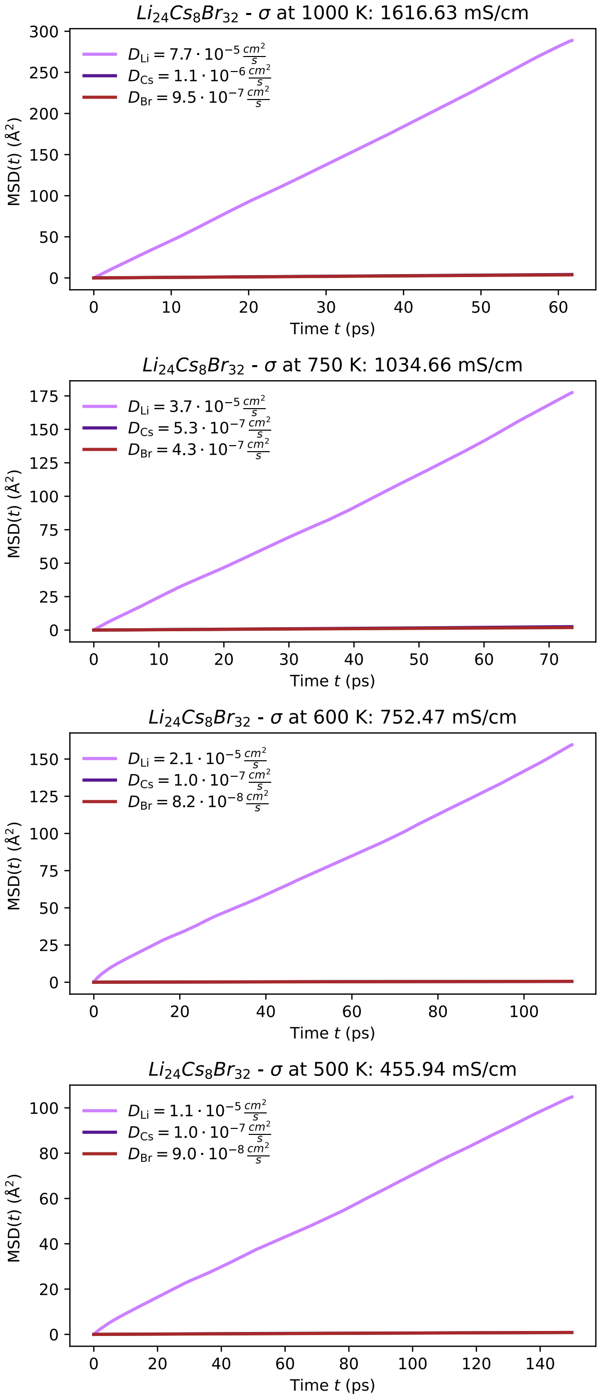

Fig. S4 MSD(t) plot of Li along with host-lattice species of Li_3CsBr_4 at all temperatures studied with FPMD.

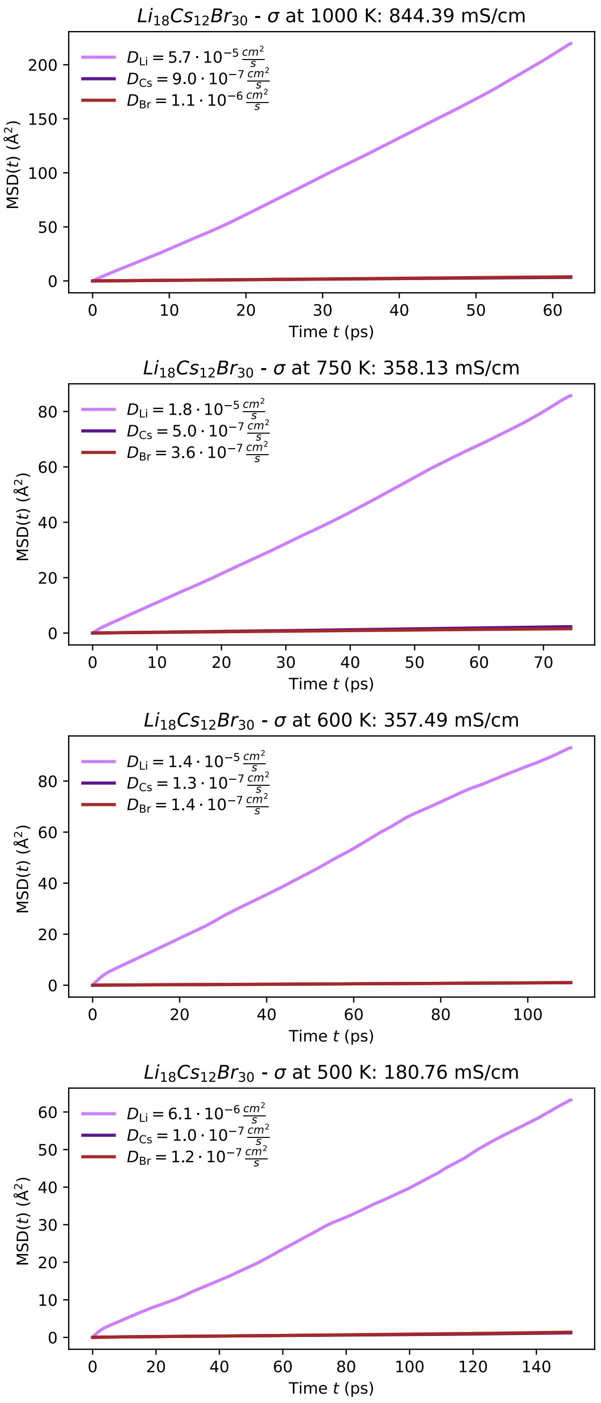

Fig. S5 MSD(t) plot of Li along with host-lattice species of $Li_3Cs_2Br_5$ at all temperatures studied with FPMD.

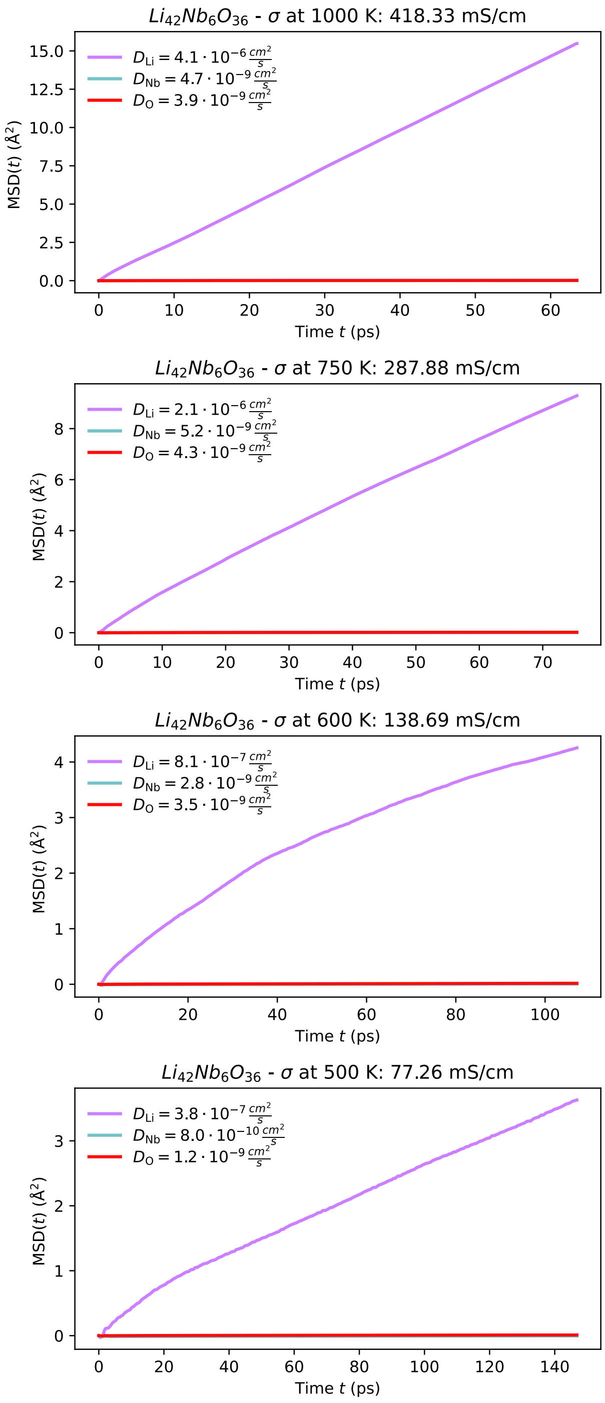

Fig. S6 MSD plot of Li along with host-lattice species of Li_7NbO_6 at all temperatures studied with FPMD

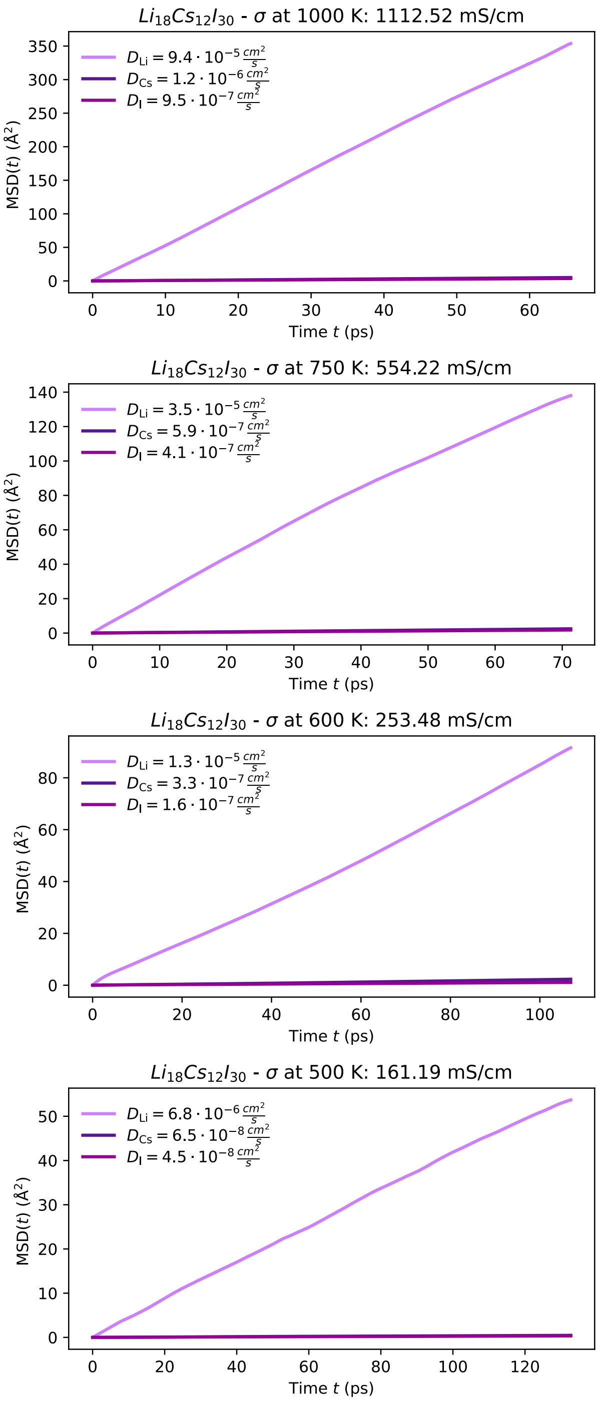

Fig. S7 MSD plot of Li along with host-lattice species of $Li_3Cs_2I_5$ at all temperatures studied with FPMD

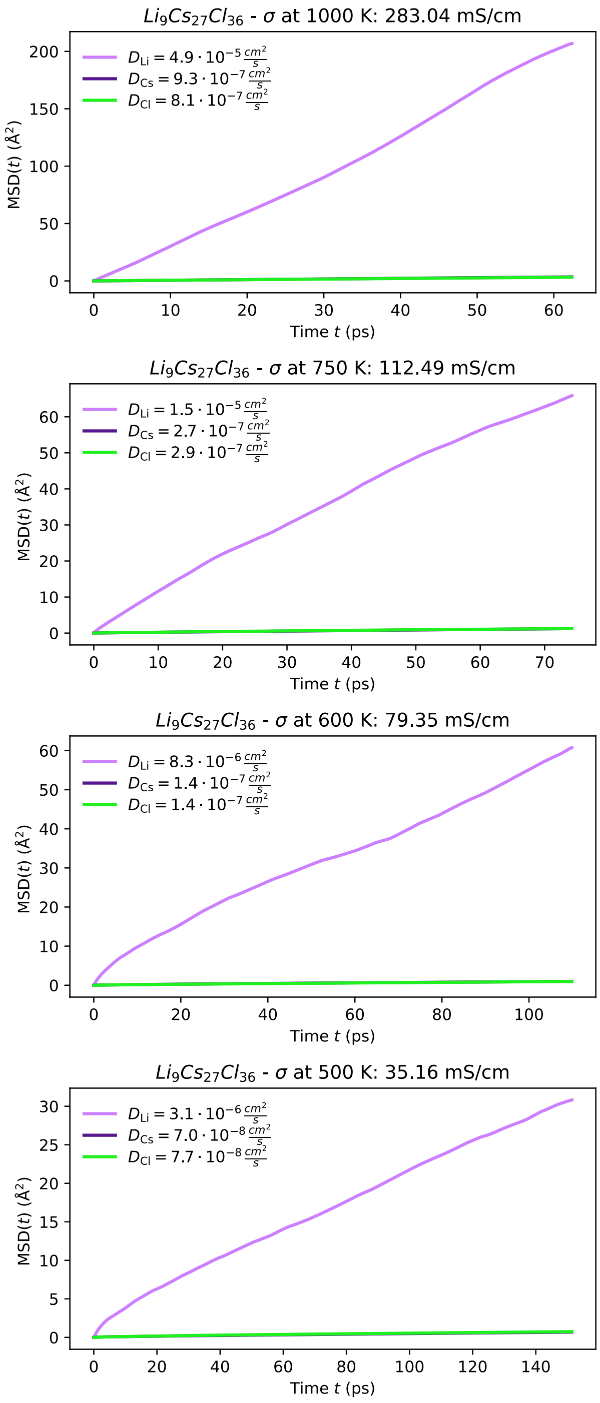

Fig. S8 MSD plot of Li along with host-lattice species of $LiCs_3Cl_4$ at all temperatures studied with FPMD

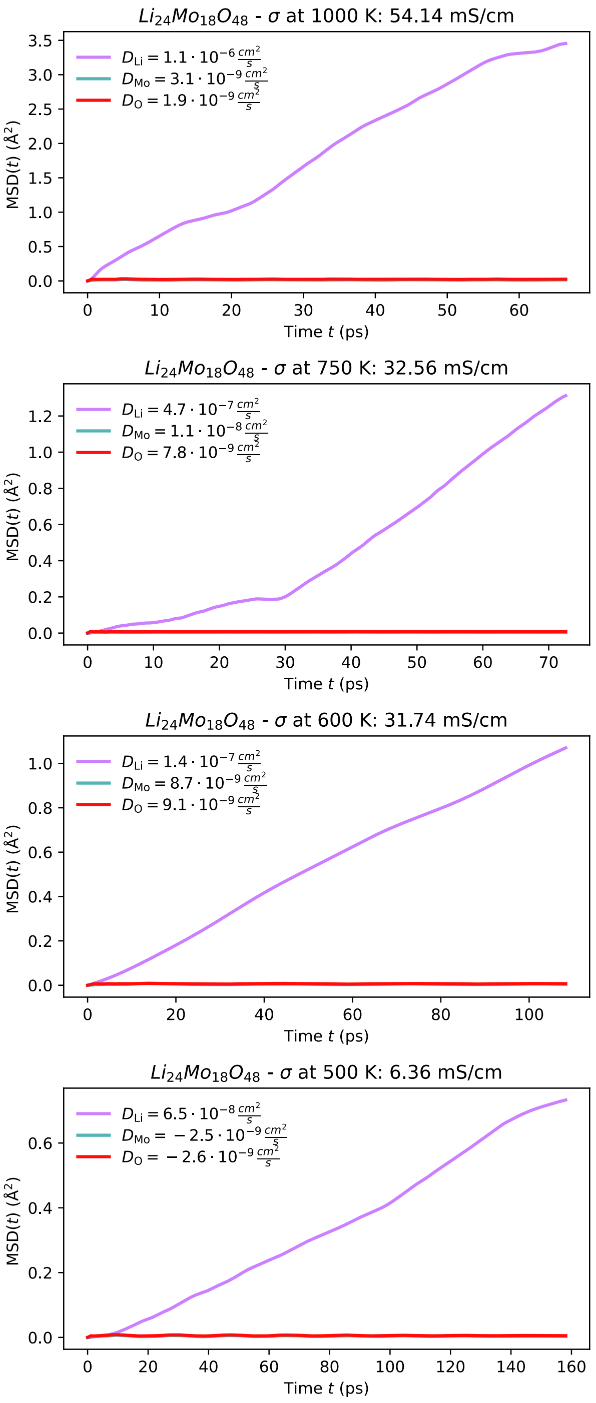

Fig. S9 MSD plot of Li along with host-lattice species of $Li_4Mo_3O_8$ at all temperatures studied with FPMD

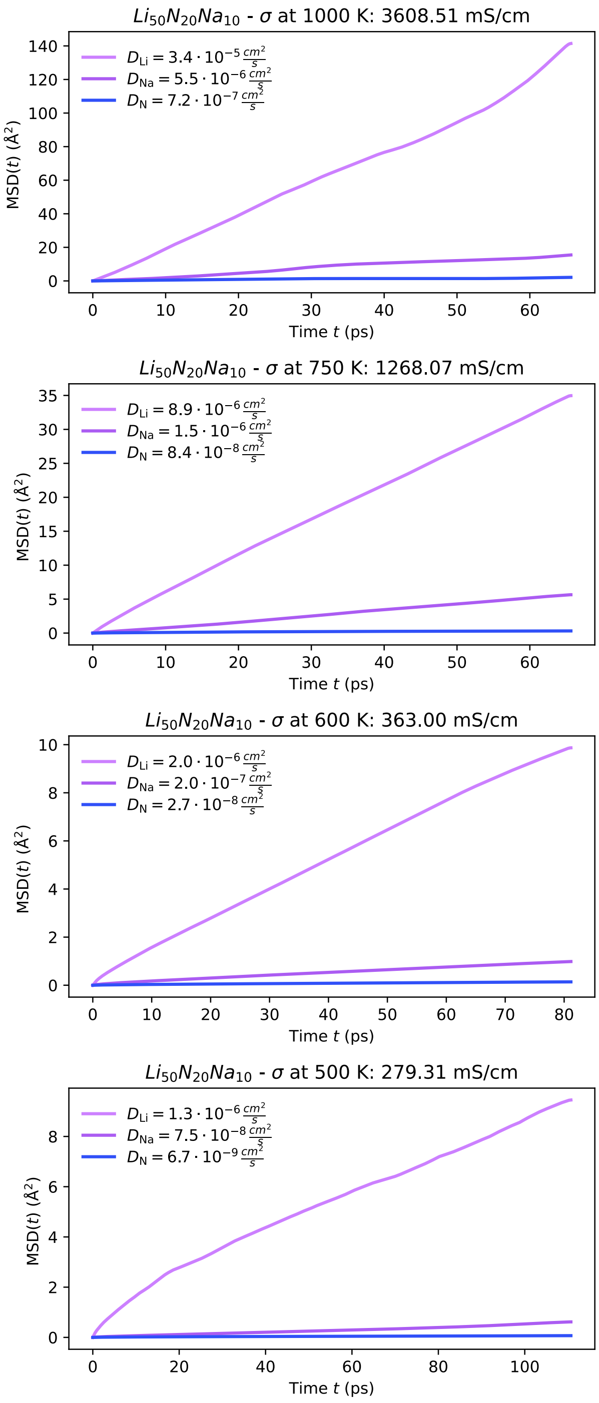

Fig. S10 MSD plot of Li along with host-lattice species of Li_5NaN_2 at all temperatures studied with FPMD

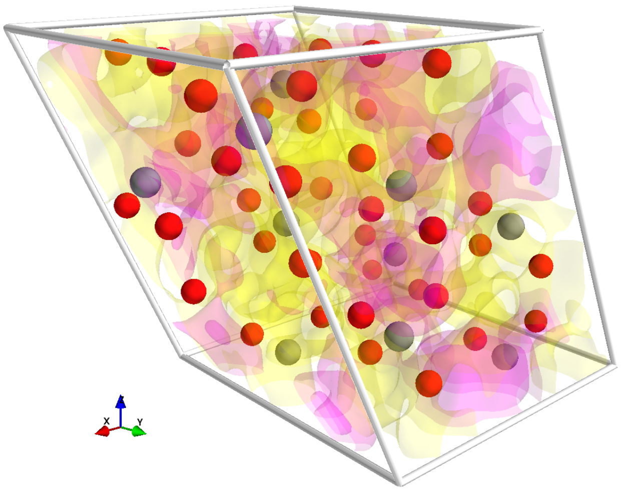

Fig. S11 Li-ion density of Li_4CO_4 at 600 K from FPMD. The pink and yellow channels clearly illustrate Li-ion diffusion in this material, establishing this materials as a 3-dimensional Li-ion conductor even at lower temperatures.

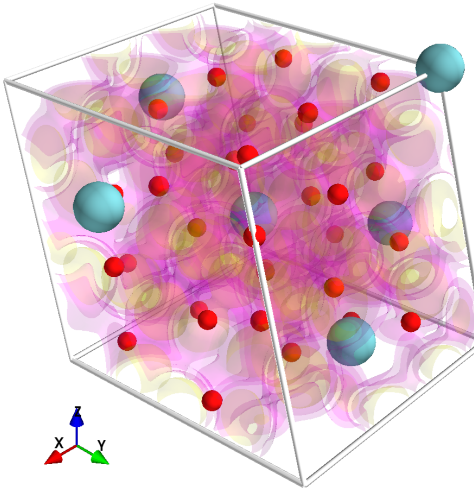

Fig. S12 Li-ion density of Li_7NbO_6 at 600 K from FPMD. The pink and yellow clouds depict Li-ion diffusion in a dispersed manner, establishing this materials as a robust 3-dimensional ionic conductor at lower temperatures.

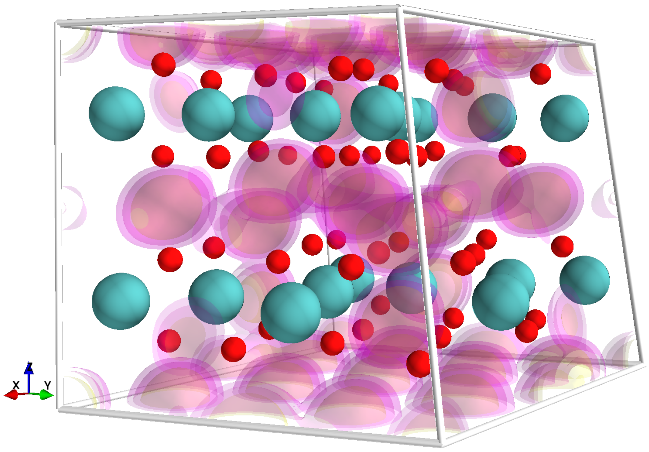

Fig. S13 Li-ion density of $Li_4Mo_3O_8$ at 600 K from FPMD. The pink clouds illustrate Li-ion diffusion in this material in a layered fashion, establishing this materials as a 3-dimensional Li-ion conductor even at lower temperatures.

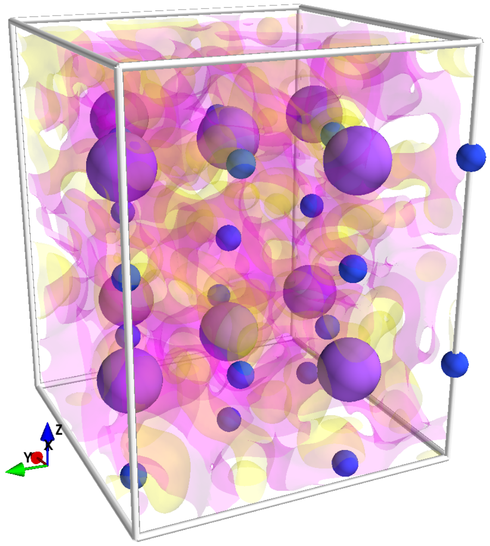

Fig. S14 Li-ion density of Li_5NaN_2 at 600 K from FPMD. The pink and yellow clouds illustrate Li-ion diffusion in a dispersed fashion, establishing this materials as a promising 3-dimensional ionic conductor at lower temperatures.

S2 Potential fast Li-ion conductors

We identify 25 structures that exhibit significant diffusion at 1000 K in our FPMD simulations, but do not display the same behaviour at lower temperatures. We show the MSD plots at 1000 K, 750 K, 600 K and 500 K.

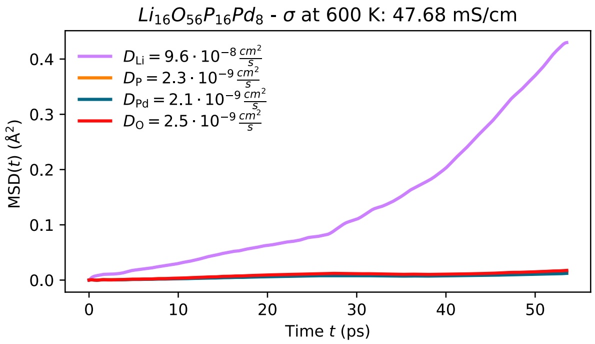

Fig. S15 MSD plot of Li along with host-lattice species of $Li_2P_2PdO_7$ at 600 K. This structure was too expensive to simulate with full-first principles so we studied it at only 600 K with FPMD.

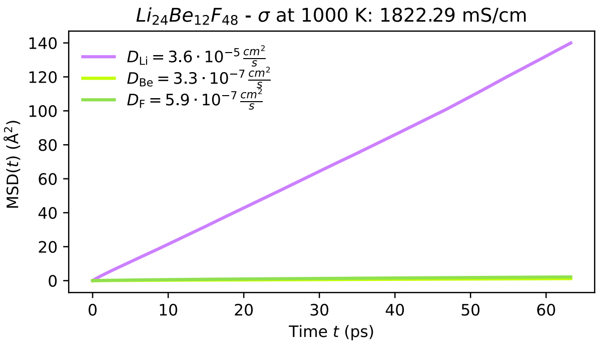

Fig. S16 MSD(t) plot of Li along with host-lattice species of Li_2BeF_4 at 1000 K, studied with FPMD.

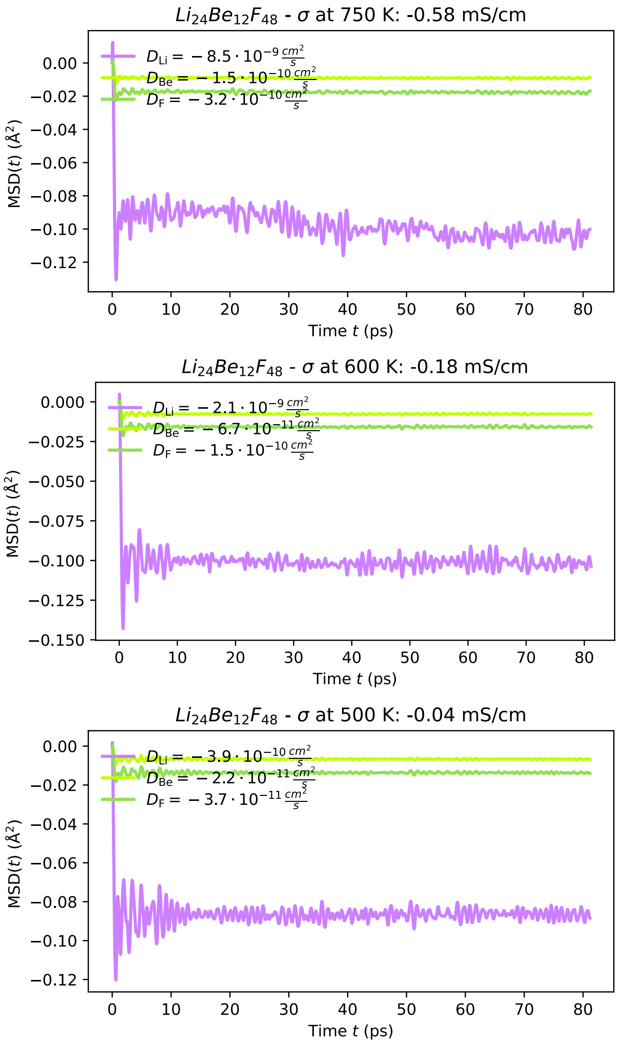

Fig. S17 MSD plot of Li along with host-lattice species of Li_2BeF_4 at 750 K, 600 K, and 500 K, studied with FPMD

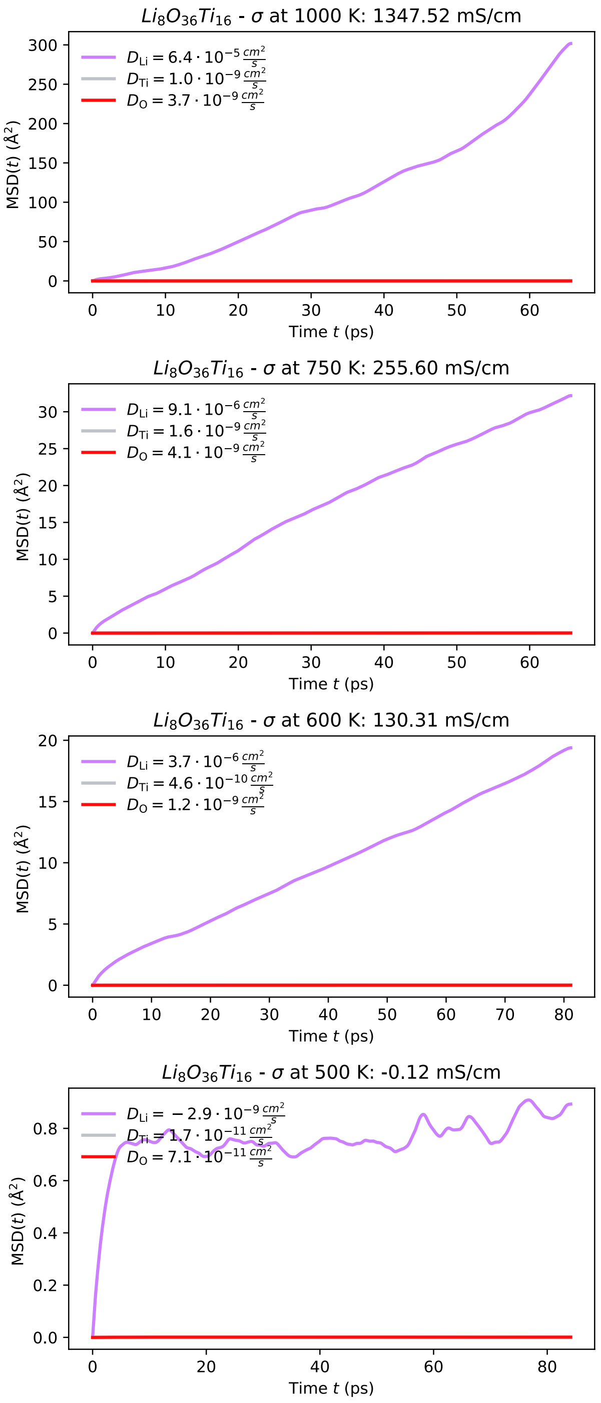

Fig. S18 MSD plot of Li along with host-lattice species of $Li_2Ti_4O_9$ at all temperatures studied with FPMD

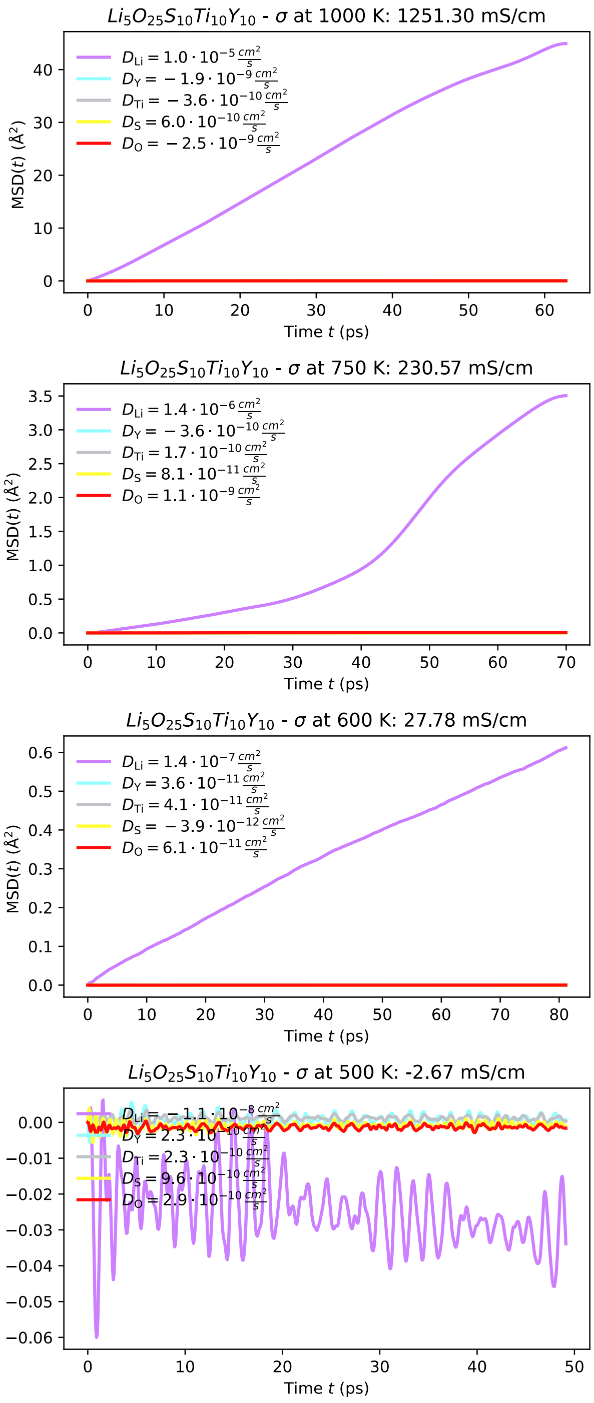

Fig. S19 MSD plot of Li along with host-lattice species of $LiY_2Ti_2S_2O_5$ at all temperatures studied with FPMD

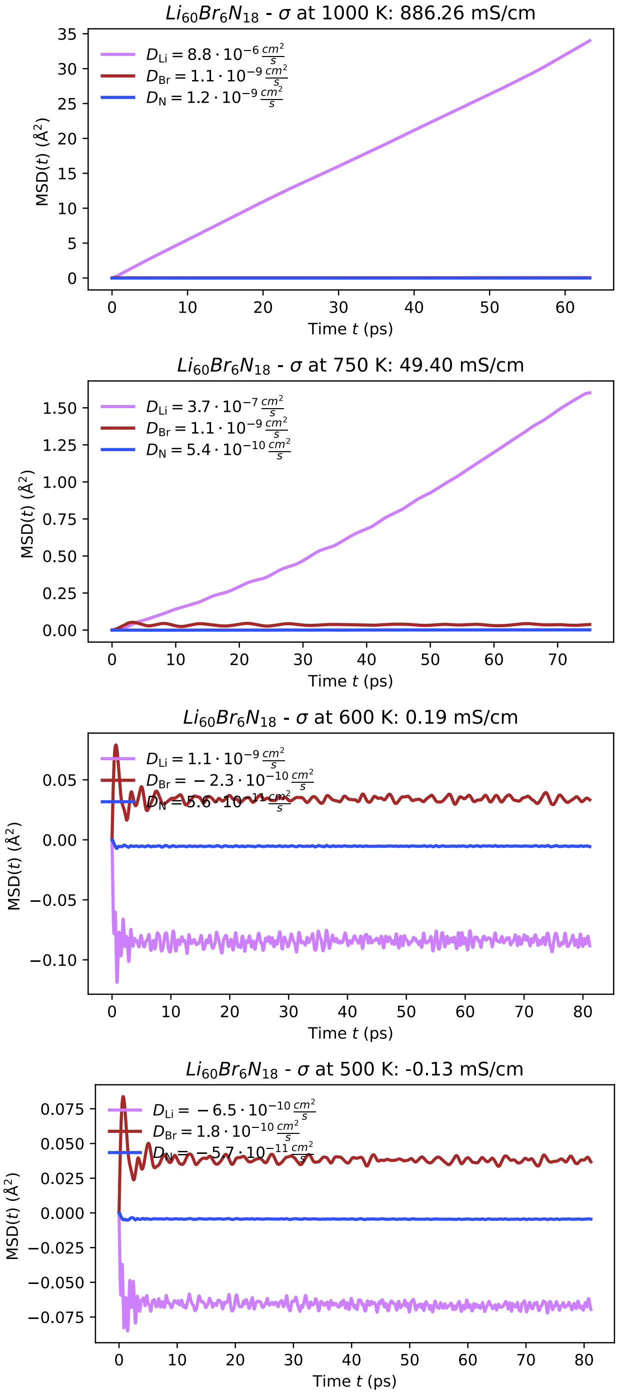

Fig. S20 MSD plot of Li along with host-lattice species of Li_10BrN_3 at all temperatures studied with FPMD

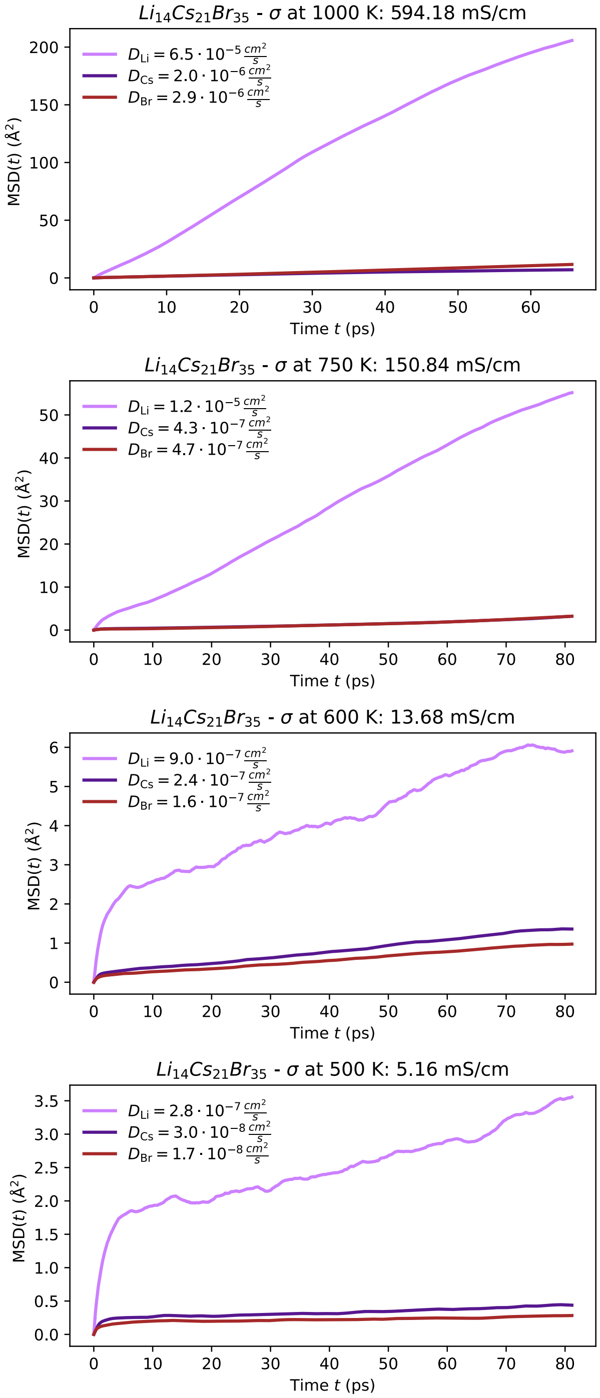

Fig. S21 MSD plot of Li along with host-lattice species of $Li_2Cs_3Br_5$ at all temperatures studied with FPMD

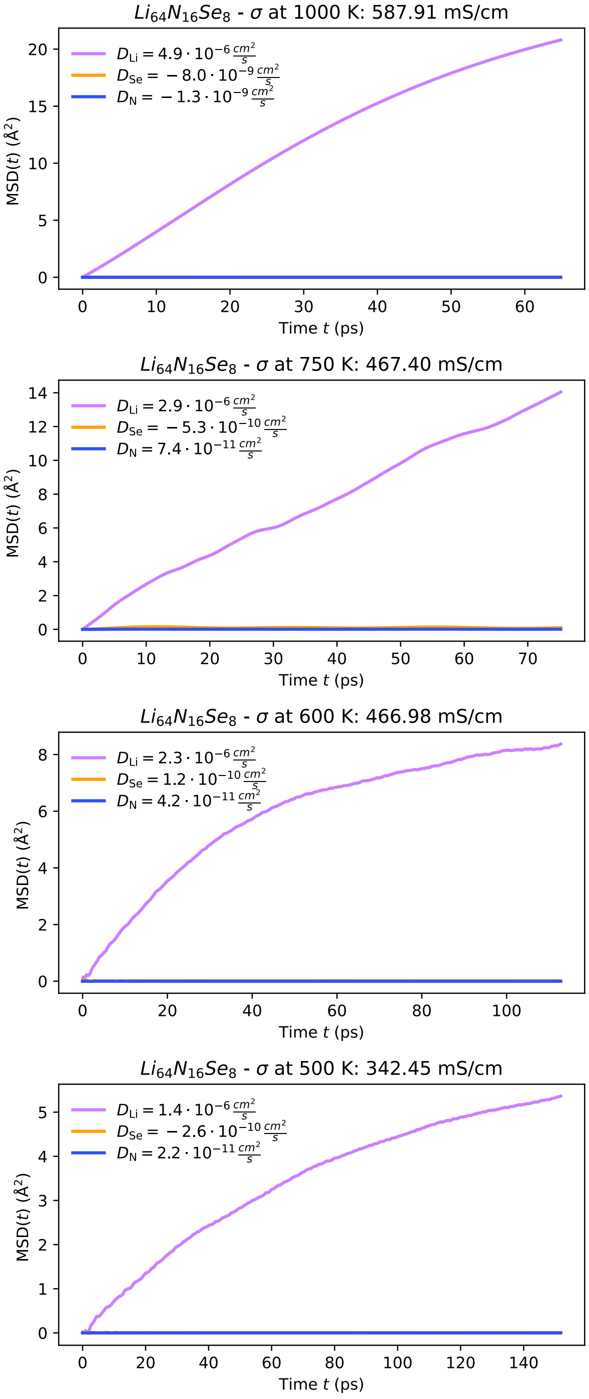

Fig. S22 MSD(t) plot of Li along with host-lattice species of Li_8SeN_2 at all temperatures studied with FPMD.

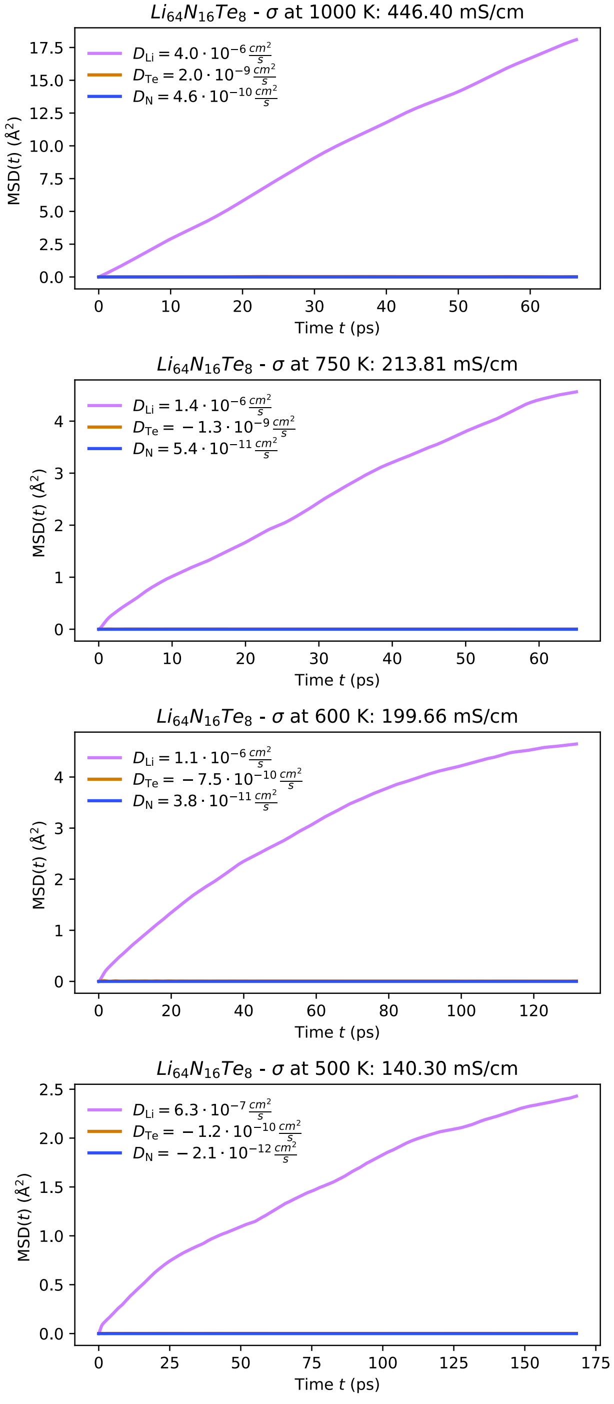

Fig. S23 MSD(t) plot of Li along with host-lattice species of Li_8TeN_2 at all temperatures studied with FPMD.

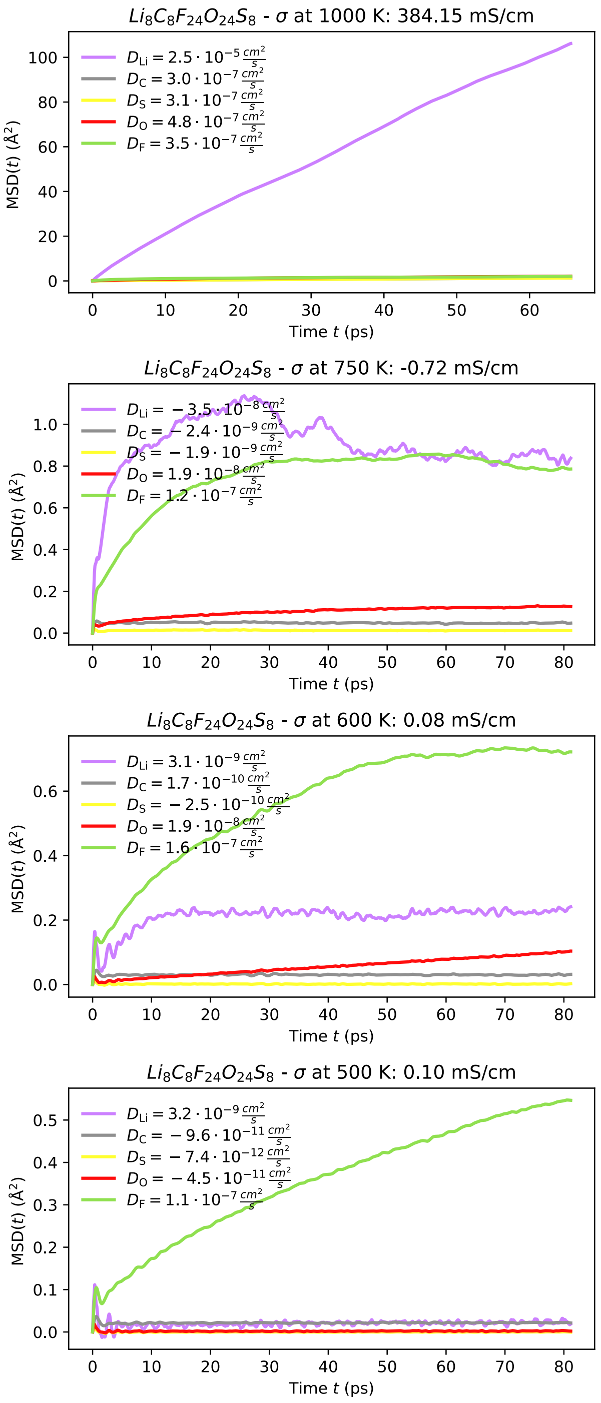

Fig. S24 MSD plot of Li along with host-lattice species of $LiCS(OF)_3$ at all temperatures studied with FPMD

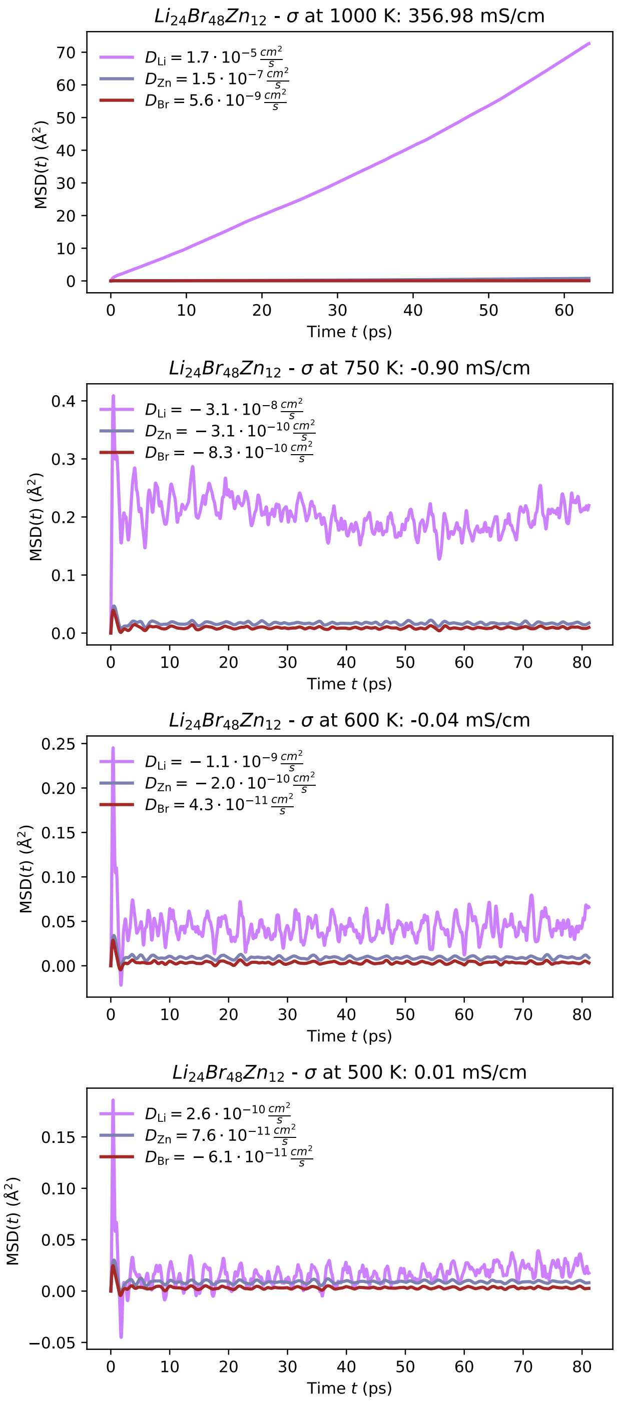

Fig. S25 MSD plot of Li along with host-lattice species of Li_2ZnBr_4 at all temperatures studied with FPMD

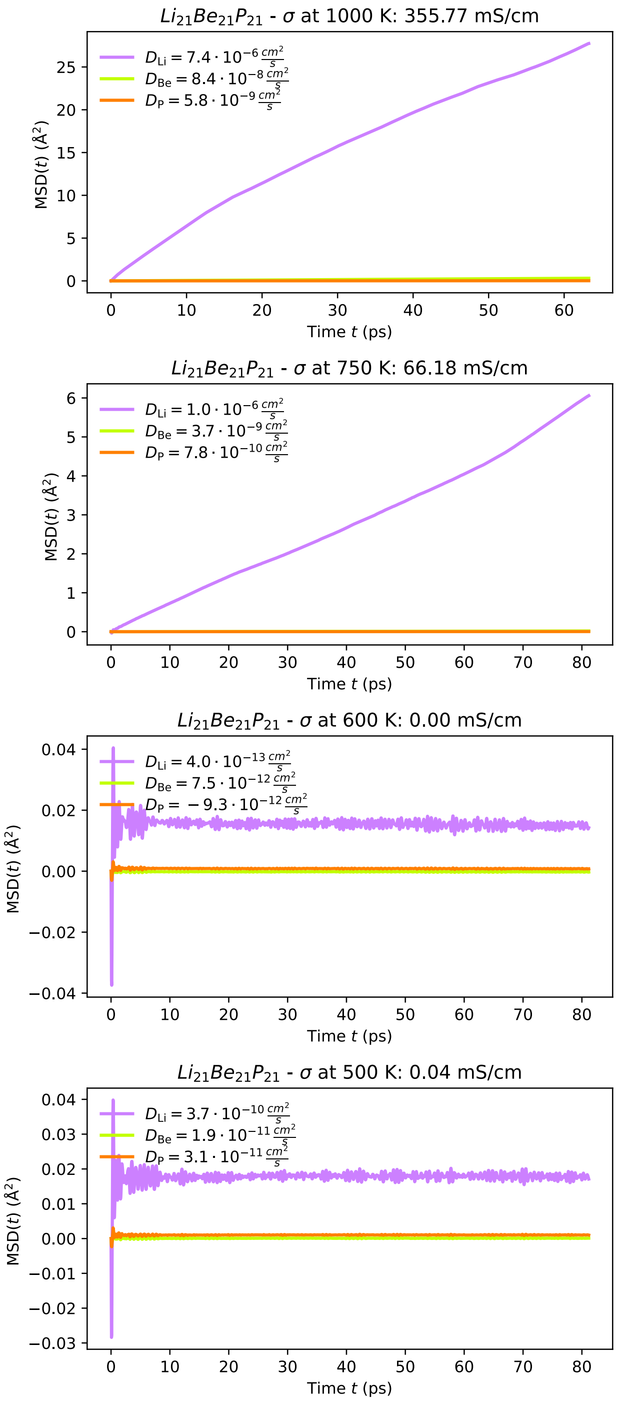

Fig. S26 MSD plot of Li along with host-lattice species of *LiBeP* at all temperatures studied with FPMD

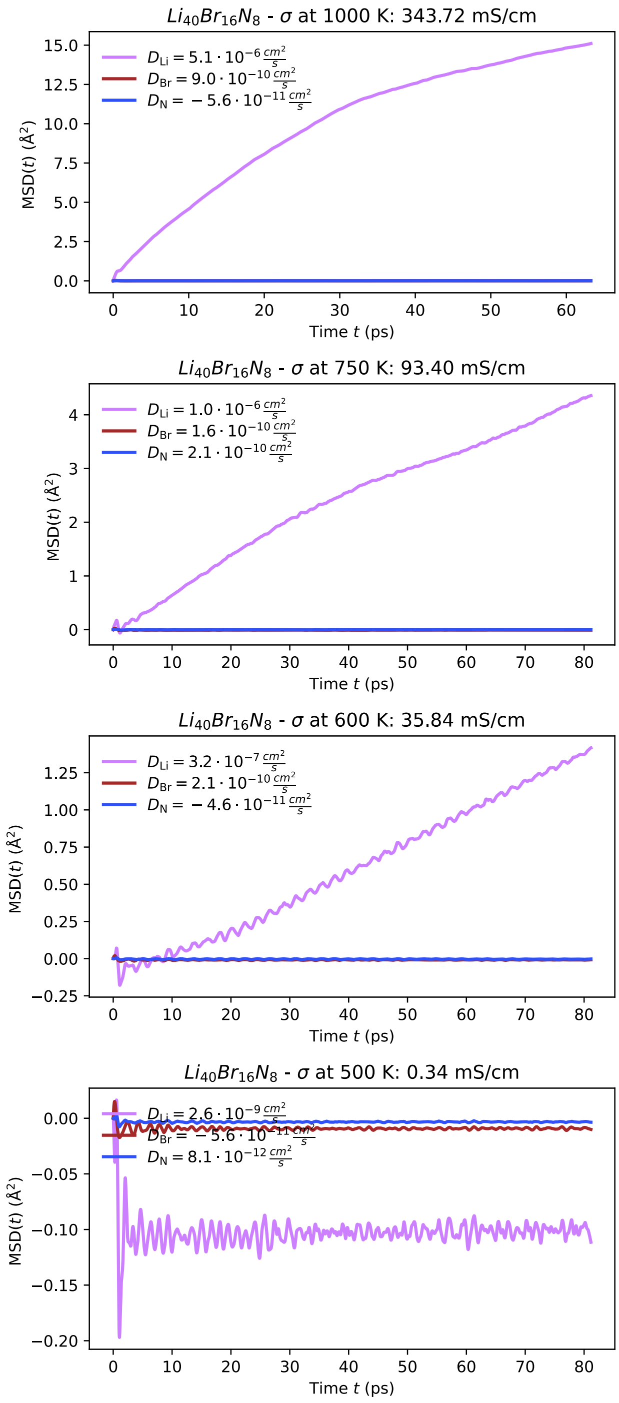

Fig. S27 MSD plot of Li along with host-lattice species of Li_5Br_2N at all temperatures studied with FPMD

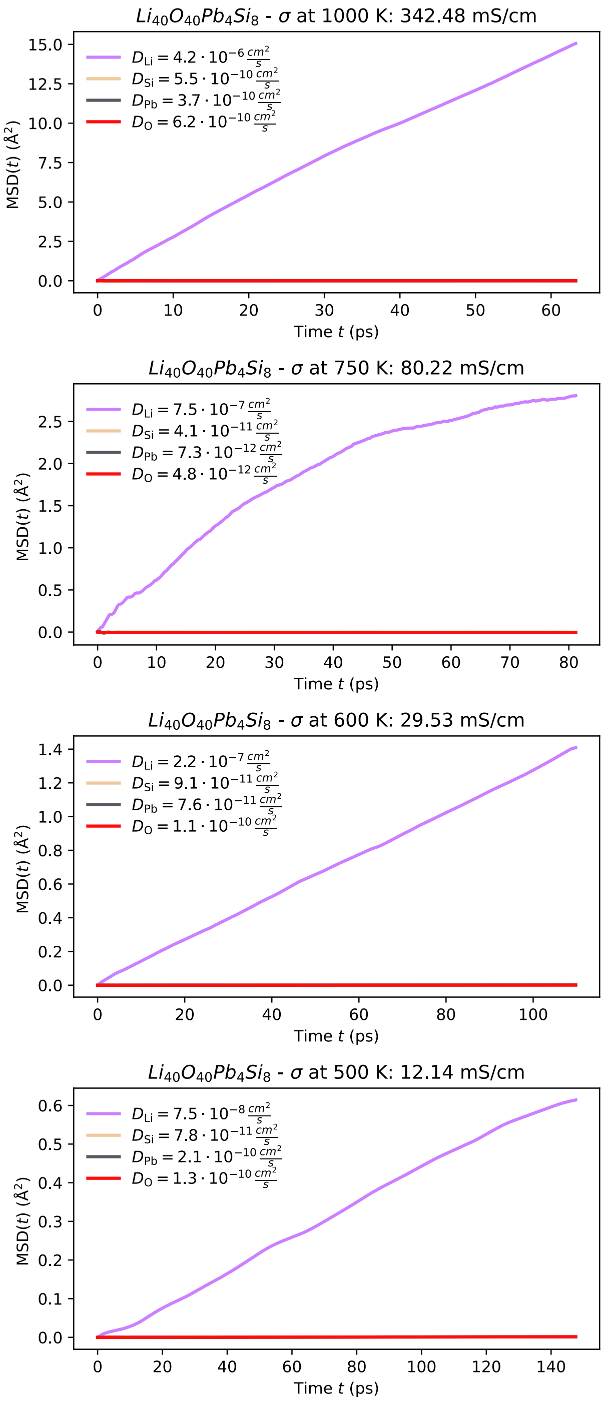

Fig. S28 MSD plot of Li along with host-lattice species of $Li_{10}Si_2PbO_{10}$ at all temperatures studied with FPMD

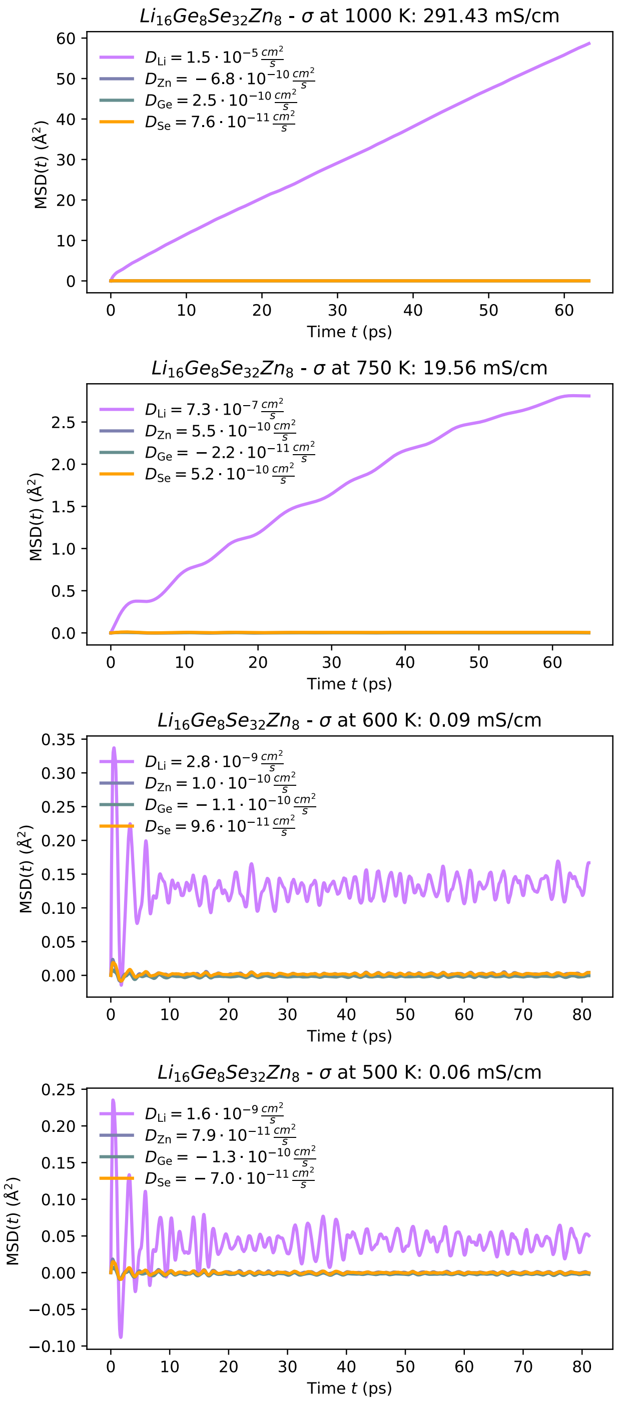

Fig. S29 MSD plot of Li along with host-lattice species of $Li_2ZnGeSe_4$ at all temperatures studied with FPMD

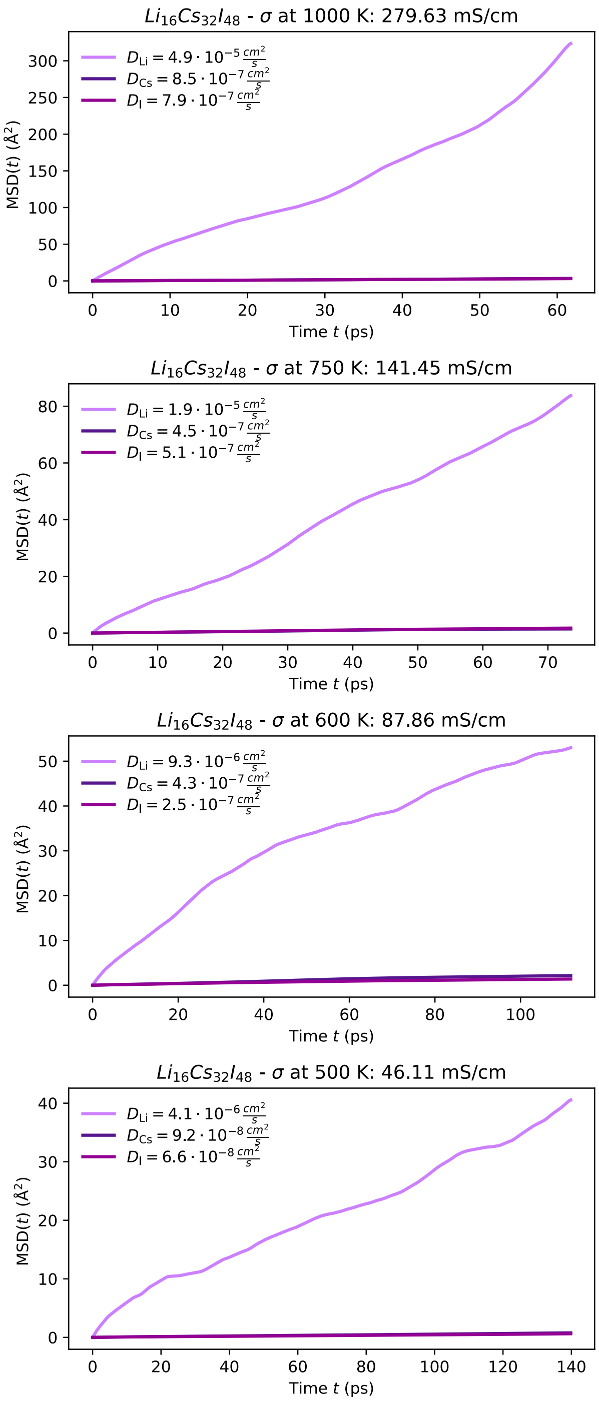

Fig. S30 MSD plot of Li along with host-lattice species of $LiCs_2I_3$ at all temperatures studied with FPMD

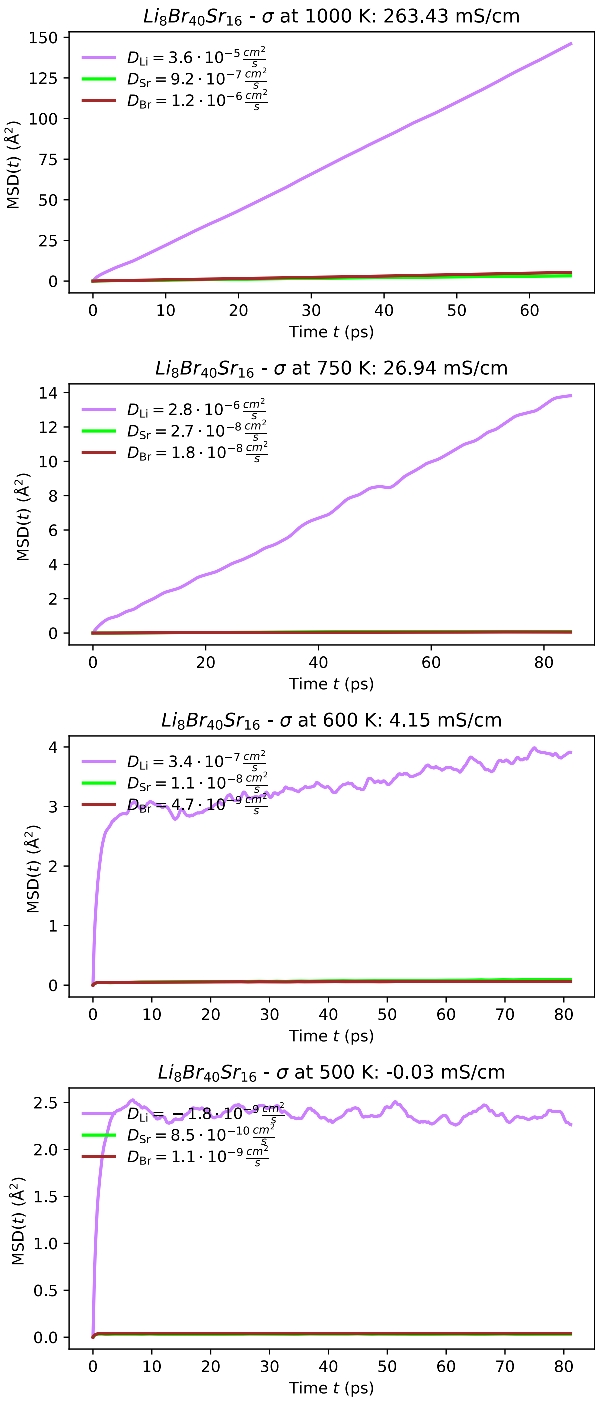

Fig. S31 MSD plot of Li along with host-lattice species of $LiSr_2Br_5$ at all temperatures studied with FPMD

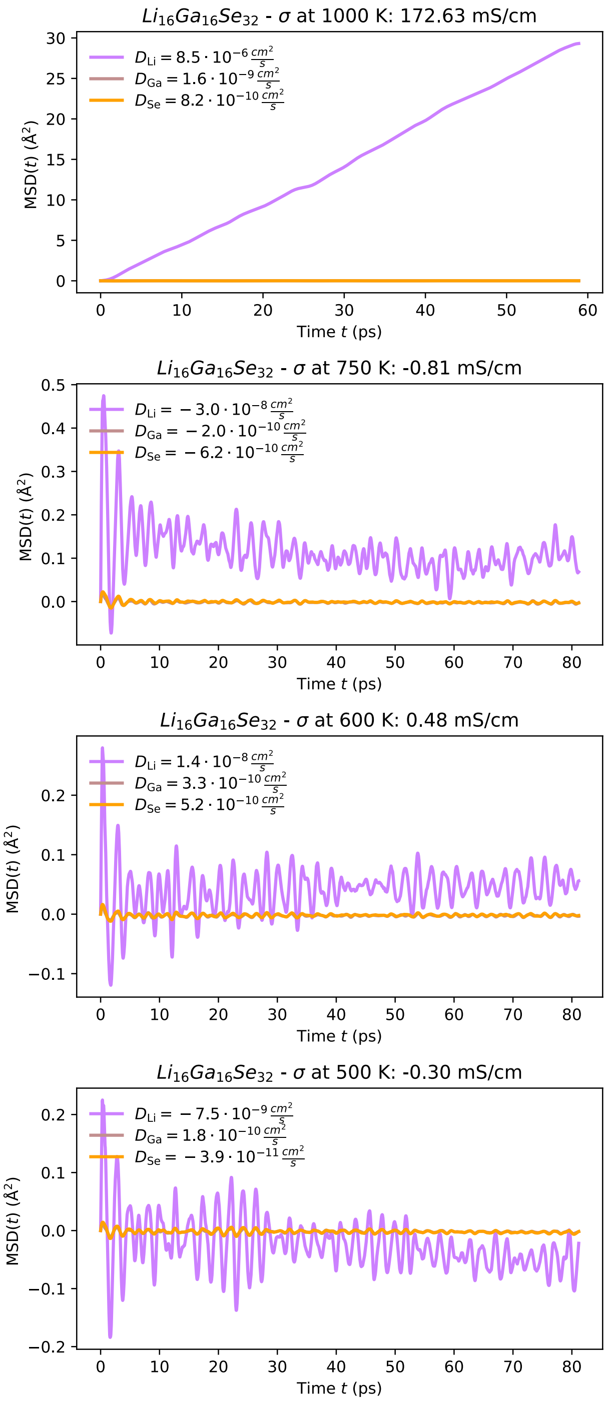

Fig. S32 MSD plot of Li along with host-lattice species of $LiGaSe_2$ at all temperatures studied with FPMD

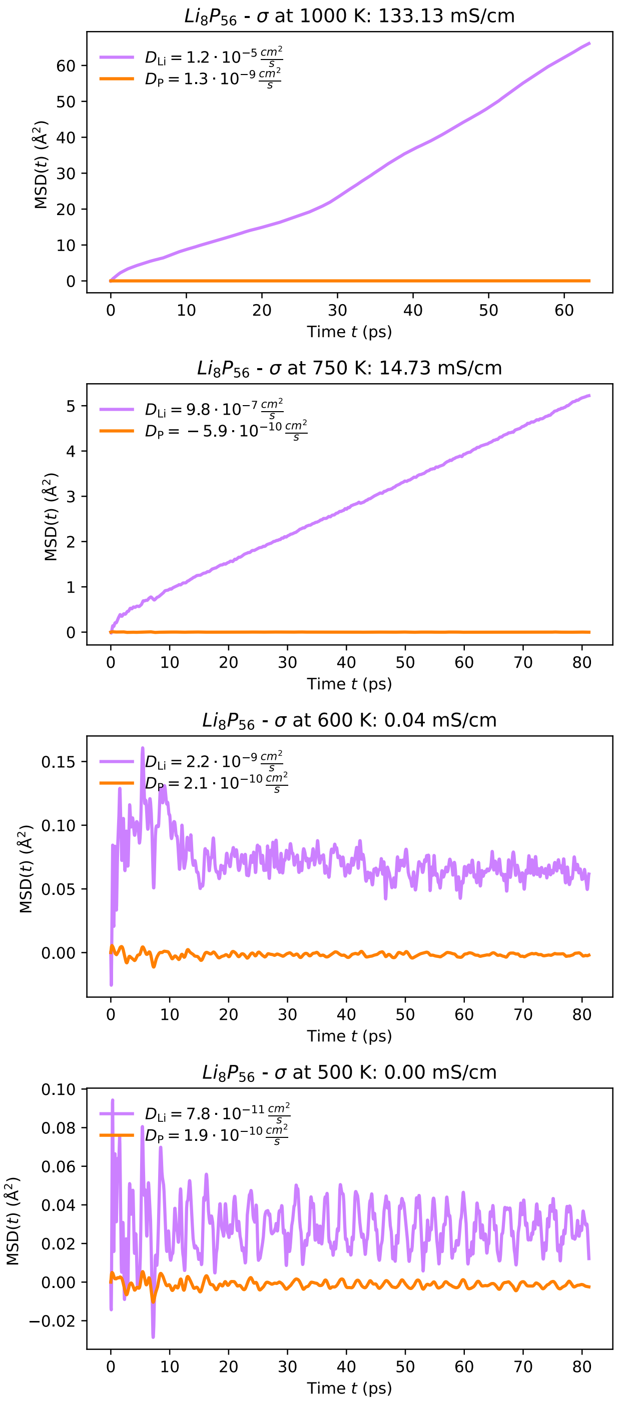

Fig. S33 MSD plot of Li along with host-lattice species of LiP_7 at all temperatures studied with FPMD

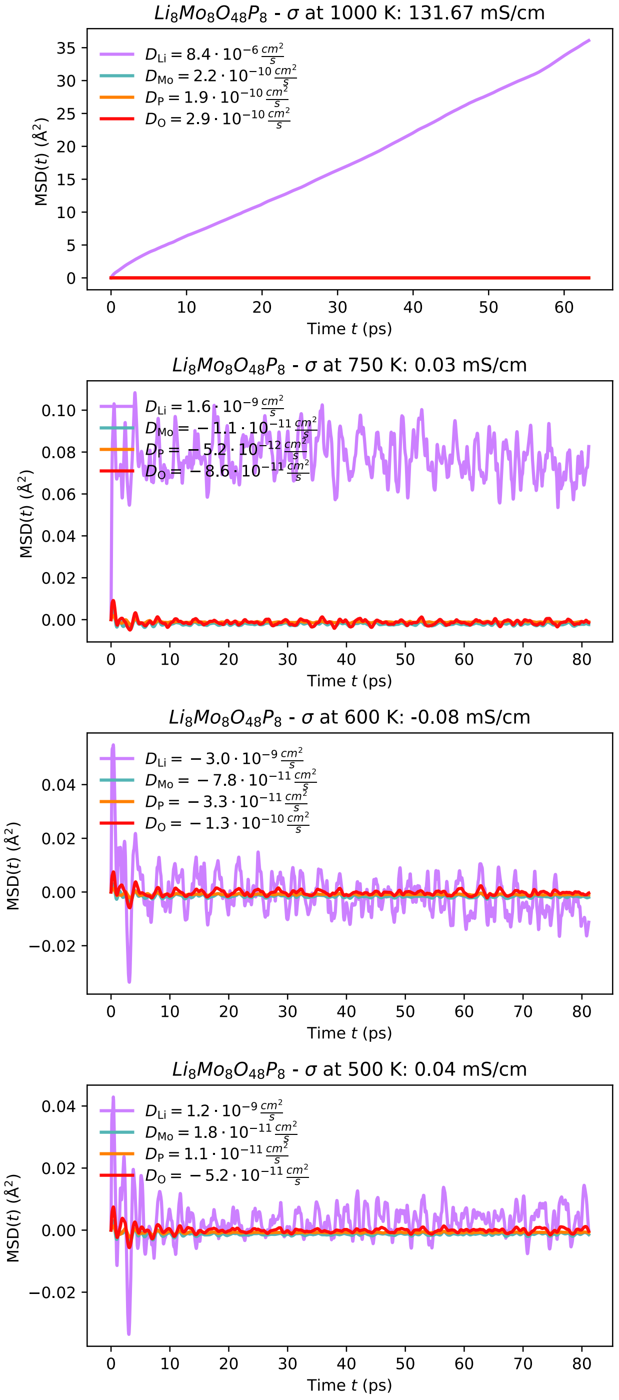

Fig. S34 MSD plot of Li along with host-lattice species of $LiMoPO_6$ at all temperatures studied with FPMD

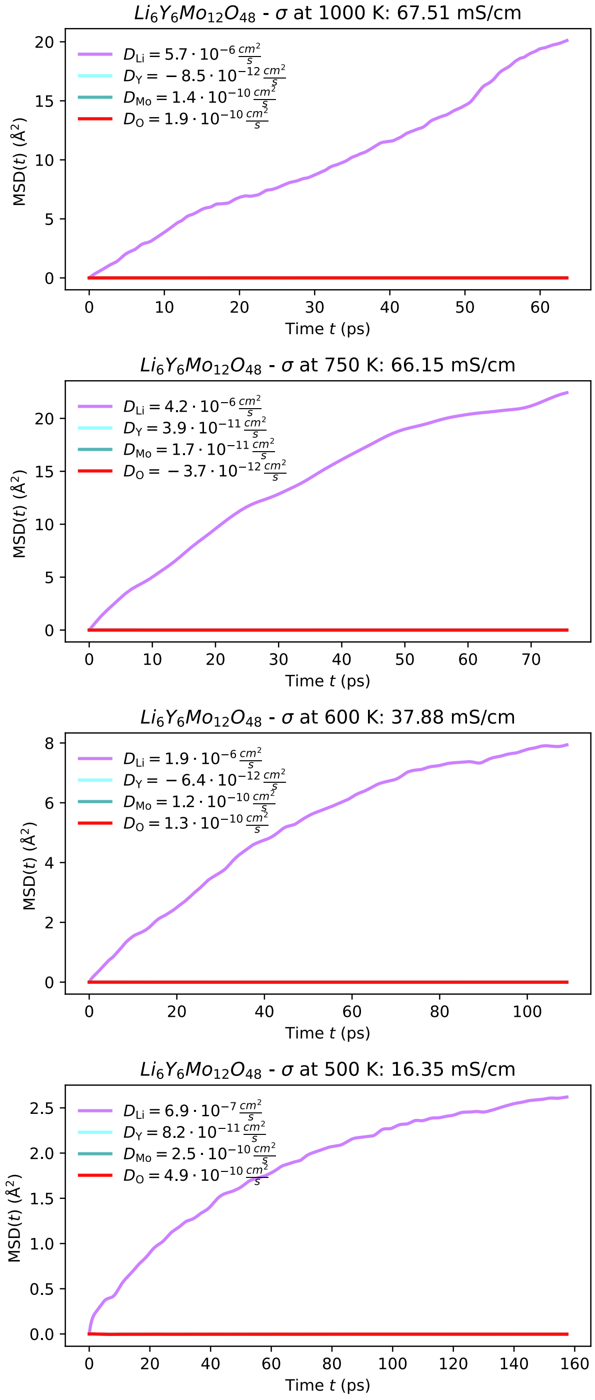

Fig. S35 MSD(t) plot of Li along with host-lattice species of $LiY(MoO_4)_2$ at all temperatures studied with FPMD.

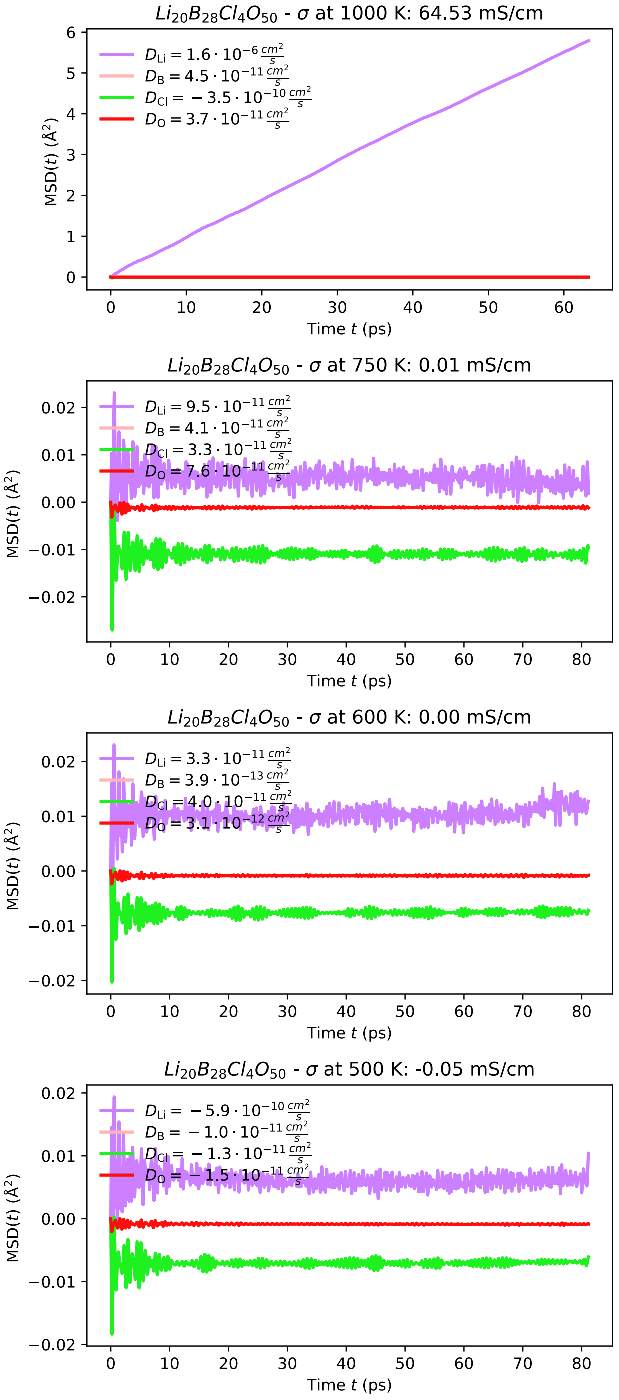

Fig. S36 MSD plot of Li along with host-lattice species of $Li_{10}B_{14}Cl_2O_{25}$ at all temperatures studied with FPMD

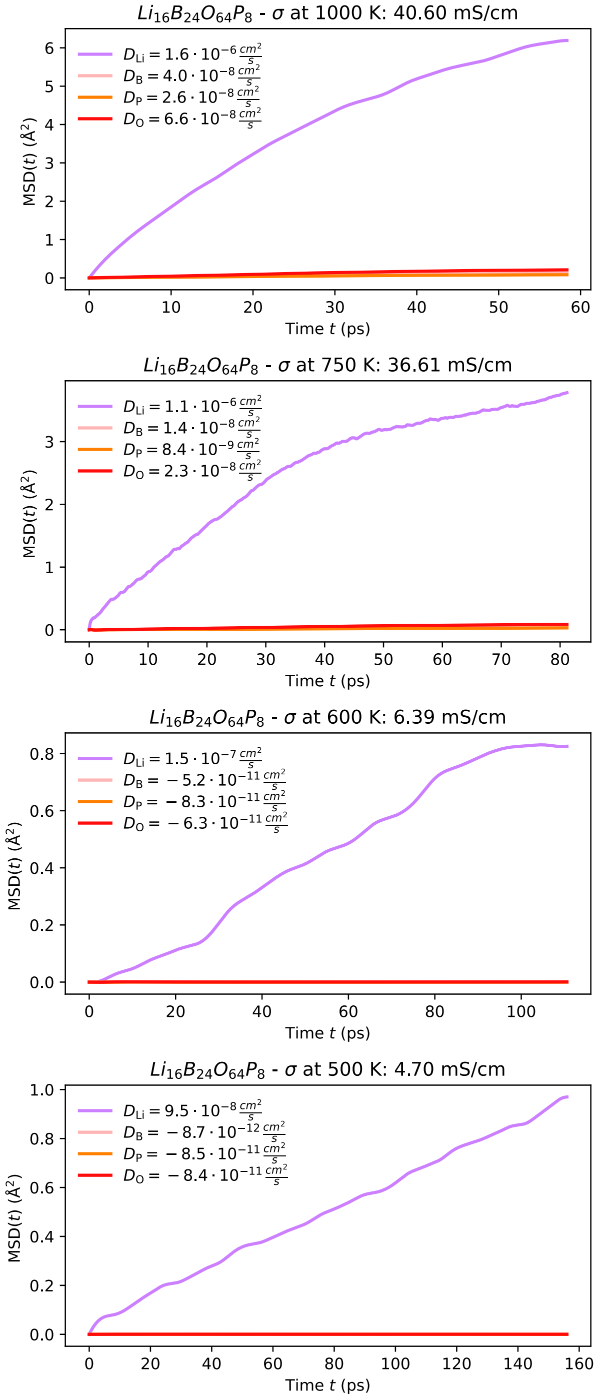

Fig. S37 MSD plot of Li along with host-lattice species of $Li_2B_3PO_8$ at all temperatures studied with FPMD

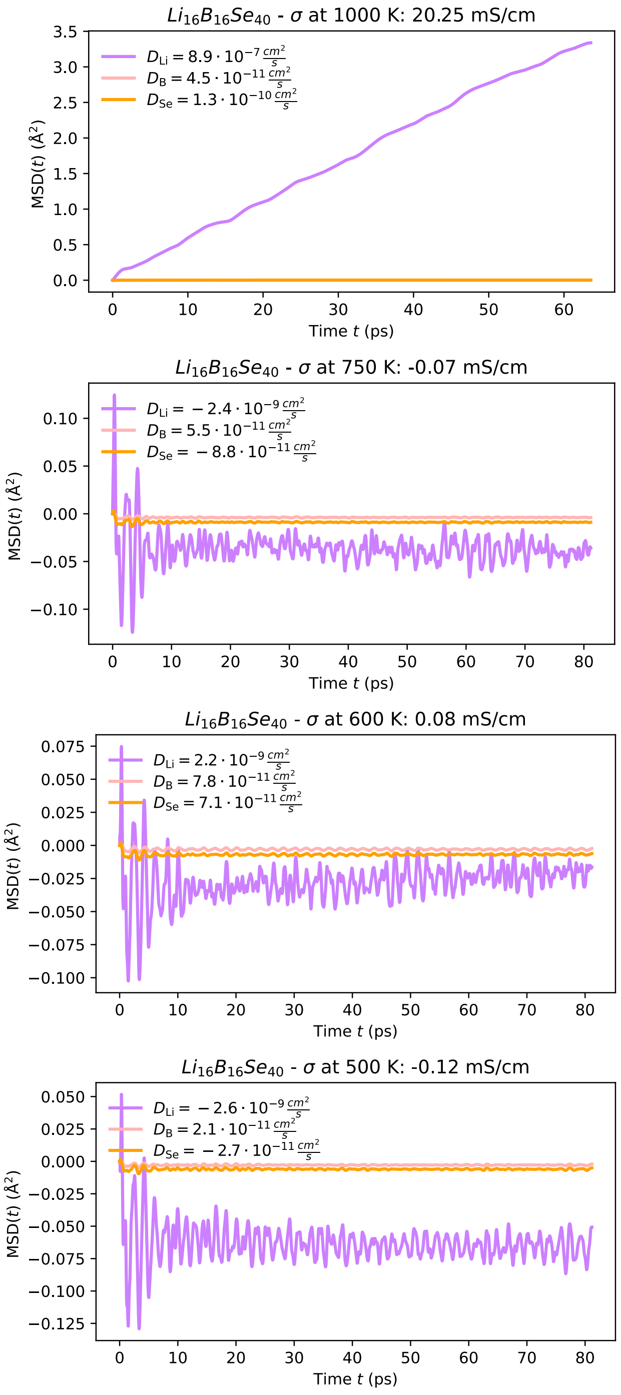

Fig. S38 MSD plot of Li along with host-lattice species of $Li_2B_2Se_5$ at all temperatures studied with FPMD

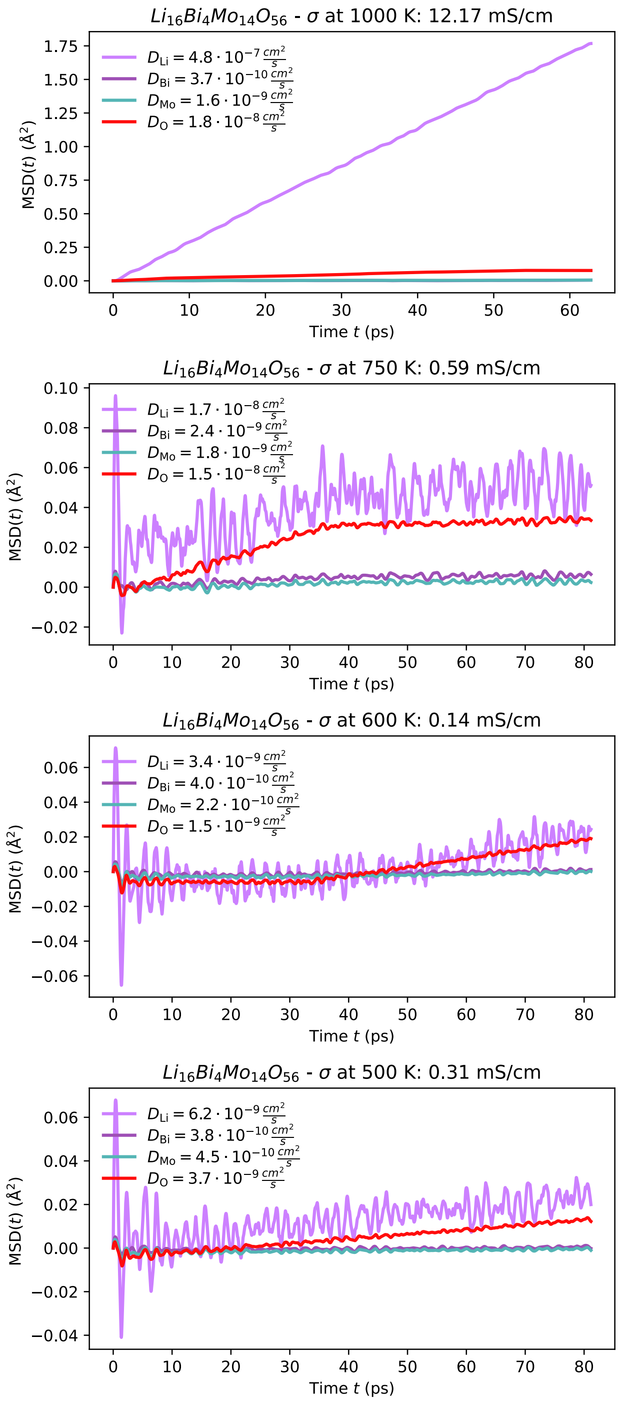

Fig. S39 MSD plot of Li along with host-lattice species of $Li_8Bi_2(MoO_4)_7$ at all temperatures studied with FPMD

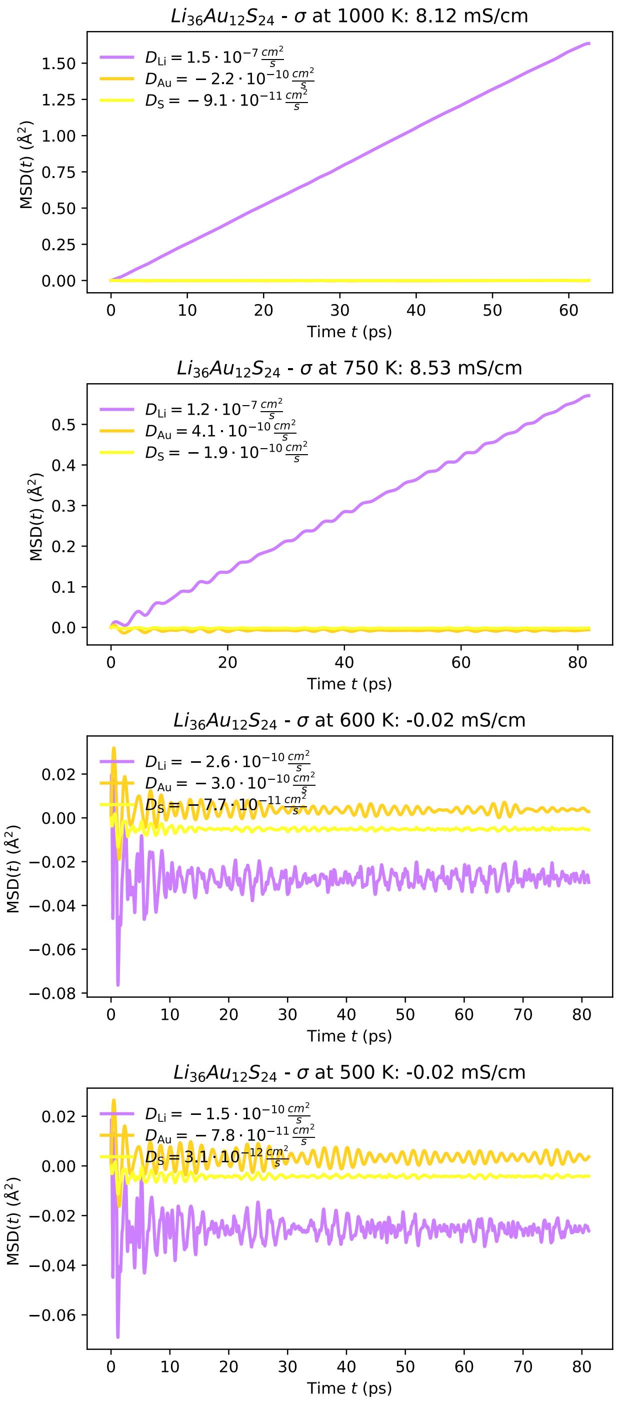

Fig. S40 MSD plot of Li along with host-lattice species of Li_3AuS_2 at all temperatures studied with FPMD

S3 Non diffusive structures

We find 18 materials that do not exhibit Li-ion diffusion in our FPMD simulations at 1000 K. We show the MSD plots only at 1000 K as we did not run FPMD at other temperatures on account of not being able to resolve diffusion at 1000 K.

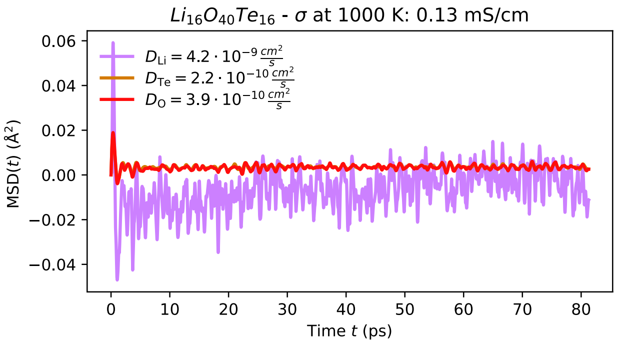

Fig. S41 MSD plot of Li along with host-lattice species of $Li_2Te_2O_5$ at 1000 K studied with FPMD

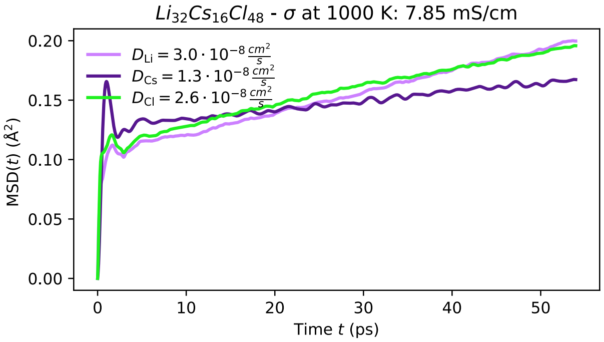

Fig. S42 MSD plot of Li along with host-lattice species of Li_2CsCl_3 at 1000 K studied with FPMD

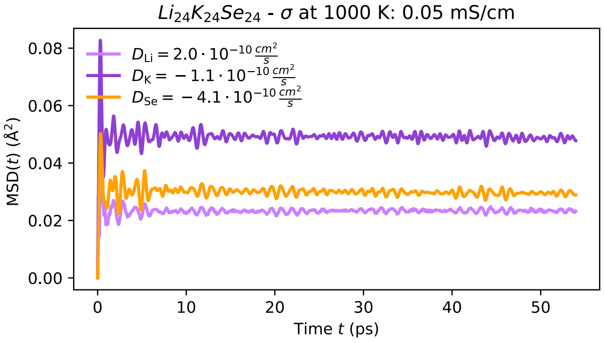

Fig. S43 MSD plot of Li along with host-lattice species of $LiKSe$ at 1000 K studied with FPMD

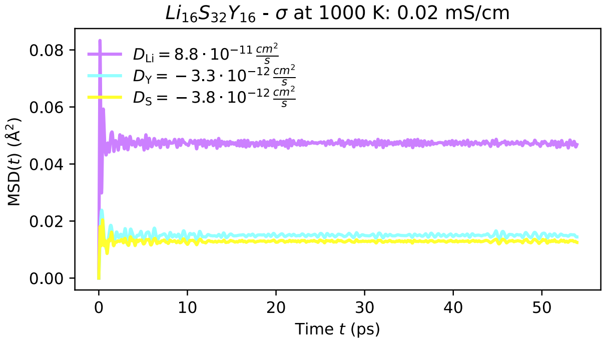

Fig. S44 MSD plot of Li along with host-lattice species of $LiYS_2$ at 1000 K studied with FPMD

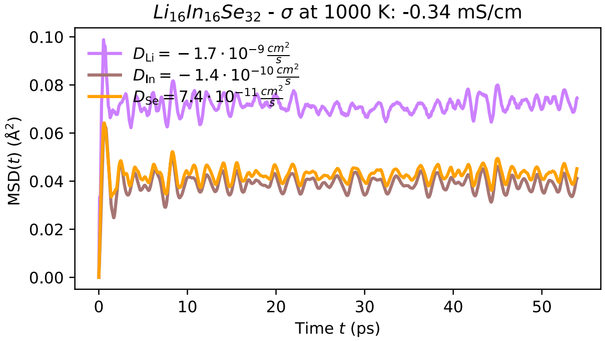

Fig. S45 MSD plot of Li along with host-lattice species of $LiInSe_2$ at 1000 K studied with FPMD

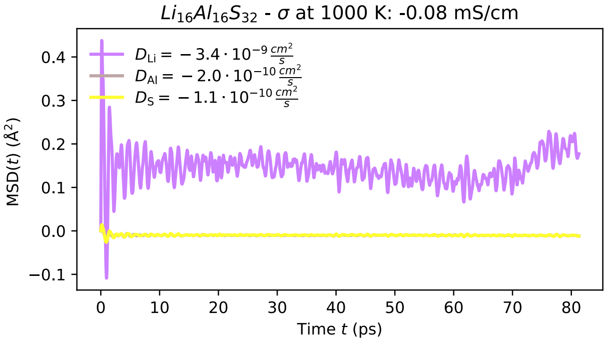

Fig. S46 MSD plot of Li along with host-lattice species of $LiAlS_2$ at 1000 K studied with FPMD

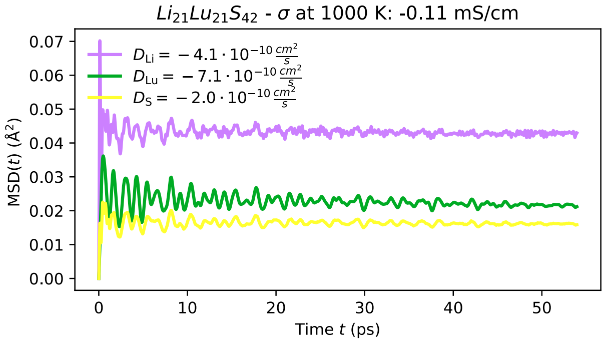

Fig. S47 MSD plot of Li along with host-lattice species of $LiLuS_2$ at 1000 K studied with FPMD

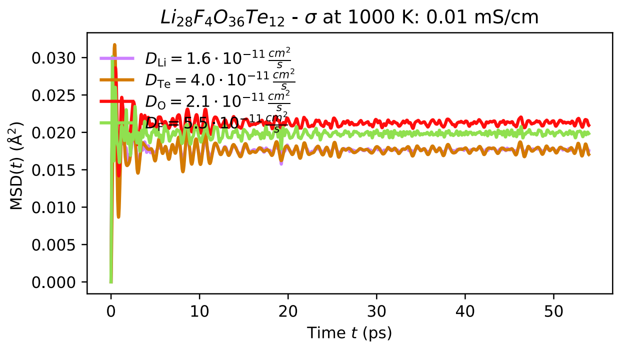

Fig. S48 MSD plot of Li along with host-lattice species of $Li_7Te_3O_9F$ at 1000 K studied with FPMD

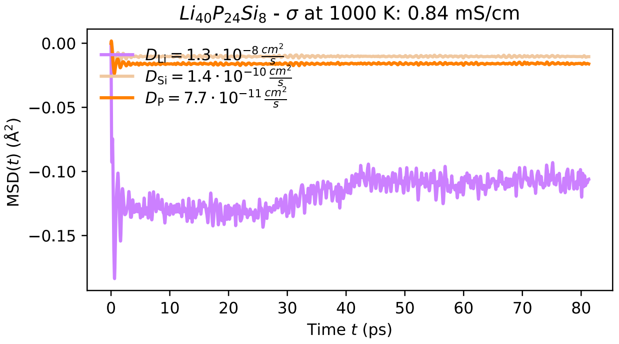

Fig. S49 MSD plot of Li along with host-lattice species of Li_5SiP_3 at 1000 K studied with FPMD

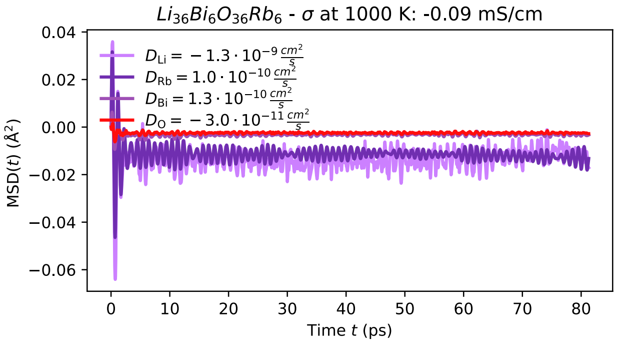

Fig. S50 MSD plot of Li along with host-lattice species of Li_6RbBiO_6 at 1000 K studied with FPMD

Fig. 5

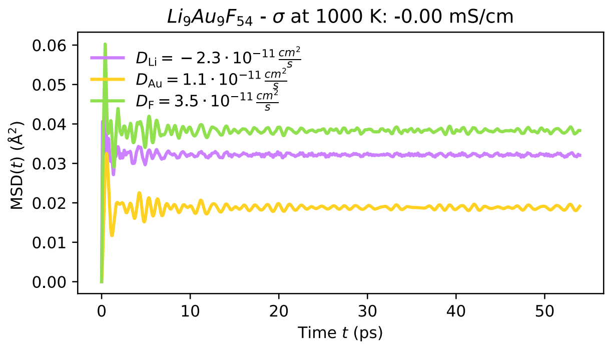

Fig. S51 MSD plot of Li along with host-lattice species of $LiAuF_6$ at 1000 K studied with FPMD

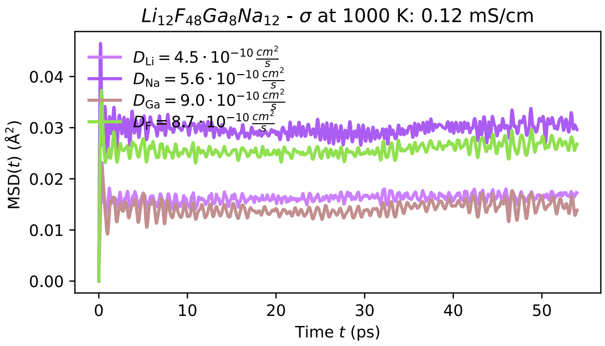

Fig. S52 MSD plot of Li along with host-lattice species of $Li_3Na_3Ga_2F_{12}$ at 1000 K studied with FPMD

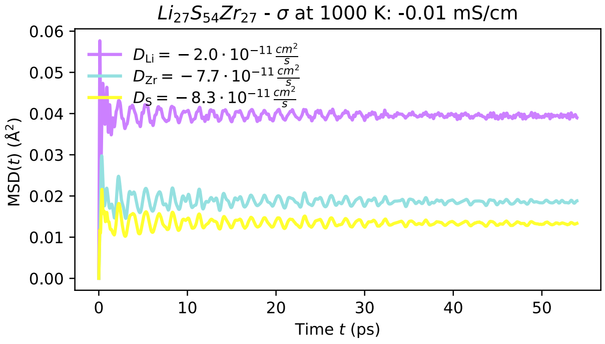

Fig. S53 MSD plot of Li along with host-lattice species of $LiZrS_2$ at 1000 K studied with FPMD

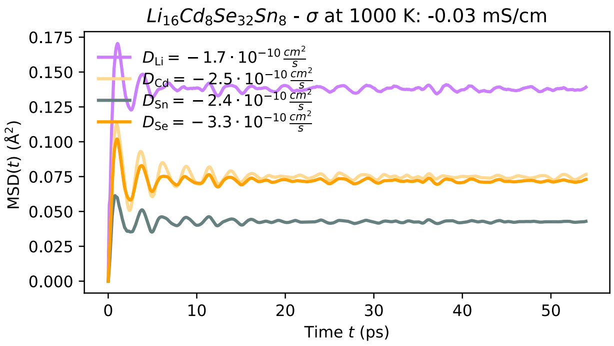

Fig. S54 MSD plot of Li along with host-lattice species of $Li_2CdSnSe_4$ at 1000 K studied with FPMD

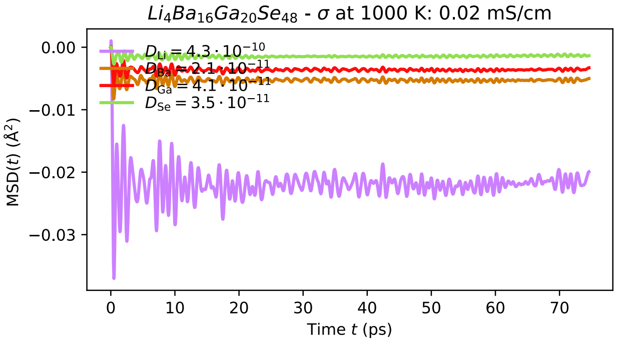

Fig. S55 MSD plot of Li along with host-lattice species of $LiBa_4Ga_5Se_12$ at 1000 K studied with FPMD

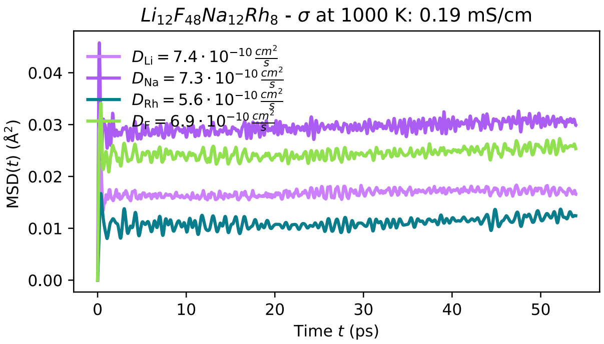

Fig. S56 MSD plot of Li along with host-lattice species of $Li_3Na_3Rh_2F_{12}$ at 1000 K studied with FPMD

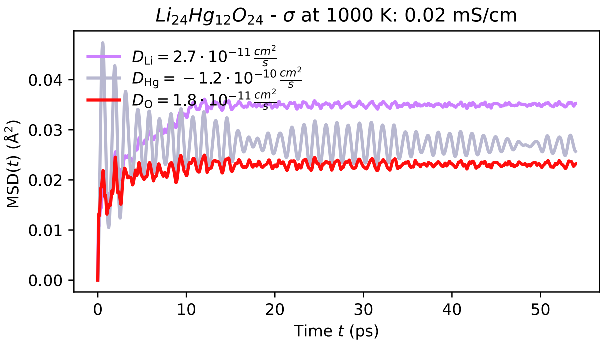

Fig. S57 MSD plot of Li along with host-lattice species of Li_2HgO_2 at 1000 K studied with FPMD

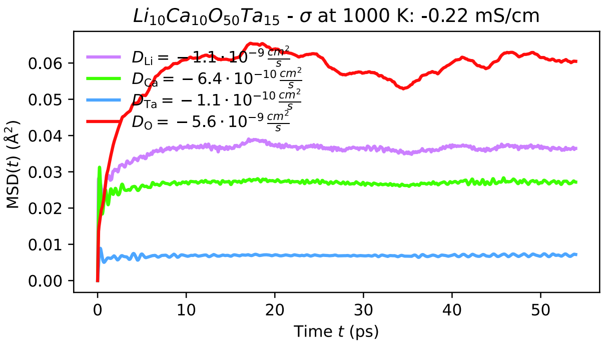

Fig. S58 MSD plot of Li along with host-lattice species of $Li_2Ca_2Ta_3O_{10}$ at 1000 K studied with FPMD
